# Maltotriose-based probes for fluorescence and photoacoustic imaging of bacterial infections

**DOI:** 10.1038/s41467-020-14985-8

**Published:** 2020-03-06

**Authors:** Aimen Zlitni, Gayatri Gowrishankar, Idan Steinberg, Tom Haywood, Sanjiv Sam Gambhir

**Affiliations:** 1 Molecular Imaging Program at Stanford, Stanford University, Stanford, CA 94305 USA; 2 Department of Radiology, Stanford University, Stanford, CA 94305 USA; 3 Department of Bioengineering, Department of Materials Science & Engineering, Stanford University, Stanford, CA 94305 USA

**Keywords:** Molecular imaging, Small molecules, Bacterial infection

## Abstract

Currently, there are no non-invasive tools to accurately diagnose wound and surgical site infections before they become systemic or cause significant anatomical damage. Fluorescence and photoacoustic imaging are cost-effective imaging modalities that can be used to noninvasively diagnose bacterial infections when paired with a molecularly targeted infection imaging agent. Here, we develop a fluorescent derivative of maltotriose (Cy7-1-maltotriose), which is shown to be taken up in a variety of gram-positive and gram-negative bacterial strains in vitro. In vivo fluorescence and photoacoustic imaging studies highlight the ability of this probe to detect infection, assess infection burden, and visualize the effectiveness of antibiotic treatment in *E. coli*-induced myositis and a clinically relevant *S. aureus* wound infection murine model. In addition, we show that maltotriose is an ideal scaffold for infection imaging agents encompassing better pharmacokinetic properties and in vivo stability than other maltodextrins (e.g. maltohexose).

## Abstract (web summary)

Sensitive diagnostic tools for bacterial infections of wounds and surgical sites are necessary to enable early detection and determine optimal means of treatment. Here, the authors develop a fluorescent and optoacoustic probe based on a maltotriose scaffold, which is selectively taken up by gram-positive and gram-negative bacteria.

## Introduction

Bacterial infections are of mounting medical and public concern worldwide. One primary reason for this epidemic is the overuse of antimicrobials, which enhanced the number of drug-resistant bacteria^1,2^. Furthermore, there is an increase in human life expectancy, which contributes to the high number of individuals at risk for infection and the proliferation of necessary medical procedures (i.e. surgery, arthroplasty, fracture fixations, biomedical implantations)^3^. Such procedures and associated implants are susceptible to infection^4–7^.

Wound and surgical infections create a substantial burden on patients’ quality of life and result in delayed healing and can even lead to death^7^. For example, surgical site infections (SSIs) are one of the most common types of healthcare-associated infections and occur in 2–5% of patients undergoing surgery in the United States. This translates to around 400,000 SSIs for an average of 15 million procedures performed annually in the United States. In addition to increasing the duration of hospitalization, SSIs increase treatment cost as well as mortality risk by 2–11-fold^8^. Unfortunately, many of these infections are only diagnosed after becoming systemic or having caused significant damage to key organs, making it harder and more costly to treat due to the high bacterial burden. It is therefore important to develop tools to noninvasively detect bacterial infections at an early stage with high sensitivity and specificity. Such tools will aid clinicians in deciding the optimal route of treatment after surgeries and can be used to monitor the effectiveness of the treatment regimen to ensure proper management of wound and surgical infections. In the clinic, bacterial infections are diagnosed through a combination of clinical, laboratory (detecting signs of inflammation, microbiology, and histopathology), and imaging assessments which are invasive, time-consuming, and/or costly^6,7,9^. Currently, imaging modalities such as X-ray, ultrasound, magnetic resonance imaging (MRI), and computed tomography (CT) provide valuable anatomical information but are only useful in diagnosing delayed and late-stage infections^9^. In order to improve sensitivity, specificity, and earlier detection, a molecular imaging strategy, which necessitates the development of imaging probes that can specifically target sites of bacterial infections, will be required. A variety of radio-imaging agents or positron emission tomography (PET) tracers for whole-body bacterial imaging have been developed and several of these are currently under evaluation in clinical trials^10^. These approaches are however dependent on proximity to cyclotrons and generators (for isotope production or collection, respectively) and experienced radiochemists for tracer production, thus limiting their availability on demand. Their use will therefore likely be restricted to major hospitals for the management of in-patients. In addition, such approaches are not recommended for use with infants, children, and pregnant women, who represent a population at high risk for infections, due to radiation exposure. To date, there are no rapid and reliable diagnostic techniques that can detect implant, wound, and surgical infections at an early stage in outpatient clinics. More specifically, an imaging tool that can aid doctors in emergency rooms (ERs) and field hospitals to quickly diagnose bacterial wound infections and determine the extent of infection can potentially change clinical management.

Fluorescence imaging (FLI) relies on the detection of emission signals from fluorescent probes upon excitation at their appropriate absorbance wavelengths^11,12^. FLI of bacterial infections gained attention due to its many advantages such as high resolution, real-time imaging capabilities, ease of use, and low cost^11^. Constricted by its limited depth and penetration (~1 cm), we believe that FLI can only be implemented in superficial infection imaging (during surgery, superficial implants, or endoscopy) as well as intra-operative applications^11,12^. On the other hand, photoacoustic imaging (PAI) is an emerging imaging technique that relies on detecting ultrasound signals produced upon thermal expansion of tissue when exciting the fluorescent probe at an appropriate wavelength with an external laser^13^. PAI has been shown to be a standalone, portable tool capable of imaging endogenous signals such as melanin, and exogenous chromophores from contrast agents, with deeper imaging capabilities (up to 4 cm) than FLI and comparable resolution to MRI (~250 µm)^13–16^. In addition, PAI has been used to monitor tissue healing by imaging blood vessels as well as utilizing its ultrasound component to provide anatomical information^15–18^. Hence, PAI can be an optimal cost-effective and non-invasive tool to quickly detect bacterial infections and monitor the effectiveness of treatment at local sites (i.e. surgery and injury sites).

A number of FLI and/or PAI probes targeted to bacteria have been developed by using antibiotics (vancomycin^19,20^ or teicoplanin^21^, specific to Gram-positive bacteria), Concanavalin (targeting bacterial cell-surface mannose)^22^, antibodies (targeting the immunodominant staphylococcal antigen A, specific to *S. aureus*)^23^, boronic-acid (targeting bacterial cell-surface glycoproteins, specific to Gram-positive bacteria)^24^, enzyme-activated nanoparticles (targeting gelatinase-expressing Gram-positive bacteria)^20^ or through electrostatic and hydrophobic interactions (specific to Gram-positive bacteria)^25^. Preclinical evaluation of these probes showed promising results in FLI^19,22–24^ or PAI^20,21^ of some bacterial infections. Unfortunately, targeting the bacterial cell wall potentially limits the amount of signaling agent taken up leading to lower sensitivity. In addition, strain-specific probes will have minimal clinical impact in imaging surgery and injury-related bacterial infections since they usually occur from the presence of a variety of pathogenic bacteria. A few other examples rely on genetically encoding bacteria with reporters, such as photo-switchable chromoproteins^26,27^ and violacein^28^, have been reported. While these strategies allow non-invasive imaging of bacteria in vivo using PAI, the application of such platform would be limited to visualizing biochemistry, pathophysiological processes, and gene expression profiles in living subjects as well as imaging tumor homing bacteria^29^ and cannot be used for diagnosing bacterial infections.

A more promising bacterial-imaging strategy relies on the utility of large sugar molecules to deliver the signaling moiety into bacteria. These complex sugars (e.g. maltose, maltotriose, and maltohexose) are major sources of glucose for bacteria and are taken up in millimolar quantities^30^. A considerable advantage of such probes is their specific uptake by bacteria through the maltodextrin transporter which is not present in mammalian cells, thus allowing differentiation of bacterial infections from other diseases such as cancer and inflammation. Murthy and co-workers^30^ developed a fluorescent and an ^18^F-labeled^31^ derivative of maltohexose at the anomeric carbon and showed its effectiveness in fluorescence and PET imaging of bacterial infections in rats, respectively^32^. Recently, Pang and co-workers^33^ developed theranostic nanoparticles loaded with purpurin 18 and targeted to bacteria by surface functionalization to maltohexose. These particles showed great potential in treating bacteria using sonodynamic therapy and assessed the specificity of their particles to the infection site using FLI imaging and showed an example of PAI using their particles. In parallel, our lab has developed an ^18^F-6”-labeled maltose and maltotriose derivatives and showed their effectiveness in imaging bacterial infections through PET imaging^34–36^. While both imaging agents were specifically taken up in bacterial infections, the maltotriose derivative showed superior clearance from organs and better pharmacokinetic properties. Hence, we decided to utilize maltotriose as a scaffold for a photoacoustic and FLI agent of bacterial infections.

In this work, we report the development and evaluation of a derivative of maltotriose for photoacoustic and fluorescent imaging of bacterial infections. The fluorescent dye is attached to the anomeric carbon (reducing end) of maltotriose, which is shown to have a smaller effect on the internalization of the sugar than functionalization at the 6″-position (non-reducing end). In addition, we conduct a face to face comparison study between maltotriose and maltohexose to determine the optimum scaffold for an imaging agent targeting the maltodextrin transporter.

## Results

### Synthesis of the fluorescent probes

**Fig. 1:**
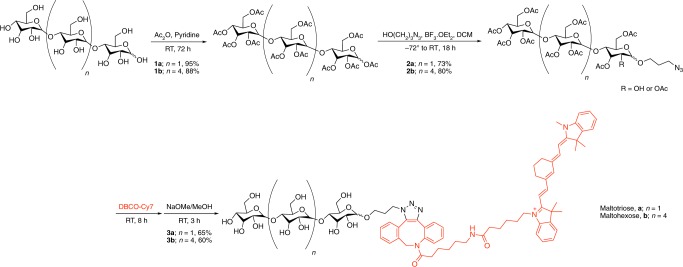
Synthesis of Cy7-1-maltotriose and Cy7-1-maltohexose. *DBCO* dibenzoyl cyclooctyne, *RT* room temperature, *Me* CH_3_, *Et* CH_3_CH_2_, *DCM* dichloro methane.

Detailed synthesis of the fluorescent probes is provided in Supplementary Methods. Briefly, azide-functionalized maltotriose intermediate at the anomeric carbon (compound **2a**) was synthesized to allow ease of functionalization with a variety of signaling agents using copper-free click chemistry. Compound **1a** was synthesized utilizing an adapted procedure^30,37^ and collected as a colorless precipitate in 94% yield. Compound **2a** was then produced from **1a** using an adapted procedure^37^ resulting in a mixture of compound **2a** as well as partially deacetylated **2a** (Fig. 1, Supplementary Figs. 1 and 2) which was also previously reported^37^. Since the glycosylation was successful and the final product was going to be fully deacetylated, the reaction was carried on the mixture. The mixture was then functionalized with a commercially available fluorescent dye coupled to dibenzoyl cyclooctyne (Cy7-DBCO), through strain-promoted azide–alkyne [3+2] cycloaddition reaction. Sodium methoxide was then added to the crude mixture to deprotect the acetate groups before purification by reverse phase high-performance liquid chromatography (HPLC) to afford compound **3a** in 65% yield (Supplementary Fig. 3). In addition, the Cy7 derivative of maltohexose was prepared following the same synthetic route producing compound **3b** in 60% yield (Fig. 1, Supplementary Fig. 4).

### In vitro evaluation

**Fig. 2:**
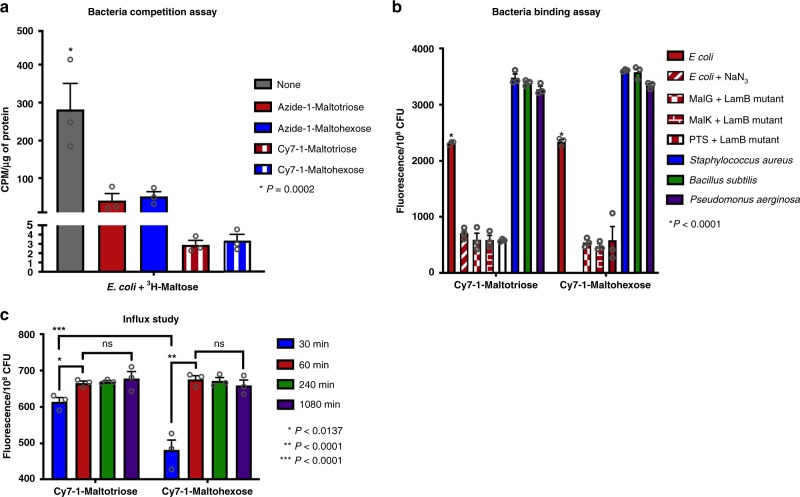
In vitro evaluation of Cy7-1-maltotriose and Cy7-1-maltohexose. **a** Bar plot representation showing the effectiveness of 1-maltotriose and 1-maltohexose derivatives in competing with the uptake of ^3^H-maltose in *E. coli*. Significant reduction in ^3^H-maltose uptake in *E. coli* was observed with prior incubation with any of the 1-maltotriose or 1-maltohexose derivatives compared to *E. coli* incubated with only ^3^H-maltose (*P* = 0.0002, *n* = 3). Data are presented as counts per minute (CPM) in each sample normalized to protein content (µg of protein). **b** Bar plot representation showing quantified fluorescence signal in *E. coli*, *Staphylococcus aureus*, *Bacillus subtilis*, and *Pseudomonas aeruginosa* after incubation with Cy7-1-maltotriose or Cy7-1-maltohexose. As a control, sodium azide-inactivated *E. coli*, and *E.* coli mutants lacking components of the maltodextrin transporter were tested. Significant reduction in Cy7-1-maltotriose uptake in inactivated *E. coli* and *E. coli* mutations was observed (*P* < 0.0001, *n* = 3). Significant reduction in Cy7-1-maltohexose uptake in *E. coli* mutations was also observed (*P* < 0.0001, *n* = 3). No significant difference in uptake between Cy7-1-maltotriose and Cy7-1-maltohexose in all bacteria strains was observed (*P* > 0.05, *n* = 3). **c** Bar plot representation of influx of Cy7-1-maltotriose and Cy7-1-maltohexose in *E. coli* overtime. Quantified fluorescence signal from influx study showcases significant increase in uptake of the probes when incubated for 60 min compared to 30 min incubation for both probes (*P* < 0.0137 and *P* < 0.0001 for Cy7-1-maltotriose and Cy7-1-maltohexose, respectively, *n* = 3). In addition, significantly higher fluorescence uptake of Cy7-1-maltotriose was observed compared to Cy7-1-maltohexose when incubated for 30 min was observed (*P* < 0.0001, *n* = 3). Bar graphs show mean and S.E.M. Statistical analysis was performed using one- and two-way ANOVA. The source data underlying Fig. 2a–c are provided in a Source Data file.

A competition binding assay between the synthesized derivatives and ^3^H-maltose, which is taken up in bacteria through the maltodextrin transporter, was conducted. A significant reduction in ^3^H-maltose uptake in *E. coli* was observed when pre-incubated with azide- or Cy7-functionalized maltotriose or maltohexose (Fig. 2a, *P* = 0.0002, *n* = 3 per study). Furthermore, addition of the azide moiety and Cy7 functional groups on the anomeric carbon of maltotriose or maltohexose did not show any effect on its ability to block the uptake of ^3^H-maltose.

A direct assessment of the ability of Cy7-1-maltotriose (**3a**) and Cy7-1-maltohexose (**3b**) to be specifically taken up by a variety of bacterial strains containing the maltodextrin transporter was also conducted. Figure 2b showcased the ability of this probe to be taken up by *E. coli*, *Staphylococcus aureus, Bacillus subtilis*, and *Pseudomonas aeruginosa*. Control studies, where azide-inactivated *E. coli* or *E. coli* mutations lacking components of the maltodextrin transporter were also evaluated and showed minimal uptake (Fig. 2b maroon, *P* < 0.0001, *n* = 3 per study).

### In vivo evaluation in *E. coli*-induced myositis murine model

**Fig. 3:**
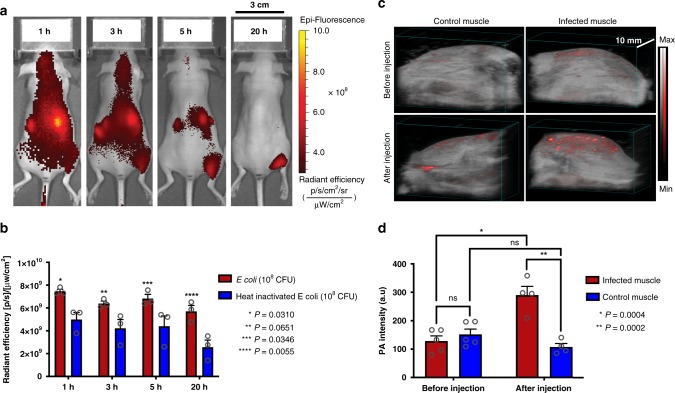
In vivo validation of Cy7-1-maltotriose in an *E. coli*-induced myositis murine model. **a** Fluorescence imaging shows accumulation of Cy7-1-maltotriose in *E. coli*-infected thigh muscle as early as 1 h post systemic injection (right thigh muscle). No evident accumulation of the agent in thigh muscle injected with 10^8^ CFUs of heat-inactivated *E. coli* (left thigh muscle). **b** Bar plot representation of quantified fluorescence signal in right and left thigh muscle overtime. As early as 1 h post-injection, a significantly higher fluorescence signal was found in muscle infected with *E. coli* (right thigh) compared to muscle infected with heat-inactivated *E. coli* (left thigh) (*P* = 0.0310, *n* = 3). **c** 3D rendered photoacoustic image overlaid on ultrasound image of a mouse’s left (control) and right (infected) thigh muscle before and 20 h post-injection of Cy7-1-maltotriose. Qualitatively, the image of the infected thigh muscle (bottom right) shows higher photoacoustic signal relative to image of the same muscle acquired before probe injection (top right) and image of the control thigh muscle (bottom left). **d** Bar plot representation of the quantified photoacoustic signal intensity in the photoacoustic images acquired before and 20 h after injection of Cy7-1-maltotriose. The quantified signal of the infected thigh muscle was significantly higher post-injection of the probe compared to that before injection (*n* = 4 and 5 respectively, *P* = 0.0004). In addition, the post-injection signal in the infected thigh muscle was significantly higher than that of the control muscle (*n* = 4, *P* = 0.0002). Bar graphs show mean and S.E.M. Statistical analysis was performed using two-way ANOVA. ns no statistically significant difference (*P* > 0.05). The source data underlying Fig. 3b and d are provided in a Source Data file.

Cy7-1-maltotriose was evaluated in vivo in an *E. coli*-induced myositis murine model. Fluorescence images of the mice over time showed rapid accumulation of Cy7-1-maltotriose in the right thigh of the mice that was infected with *E. coli*. Such accumulation was not observed in the left thigh of the mice that was injected with heat-inactivated *E. coli* (Fig. 3a). In addition, significantly higher signal intensity in the right thigh compared to the left thigh starting at the 1h imaging time point was observed (*P* < 0.0310, *n* = 3), and the signal difference increased over time (Fig. 3b).

The same animal model was then used to monitor the infection site with PAI. As a control, photoacoustic images of both infected and control thigh muscle were collected before and after probe injection. Qualitatively, a higher photoacoustic signal in the infected thigh was observed when imaged after probe injection compared to that of the control muscle (Fig. 3c, Supplementary Fig. 11). In addition, quantitative analysis of the photoacoustic signal showed ~3-fold higher PA signal in the post-probe injection images of the infected thigh muscle compared to that before injection or to control thigh muscle (*P* = 0.0004 and *P* = 0.0002, respectively) (Fig. 3d).

**Fig. 4:**
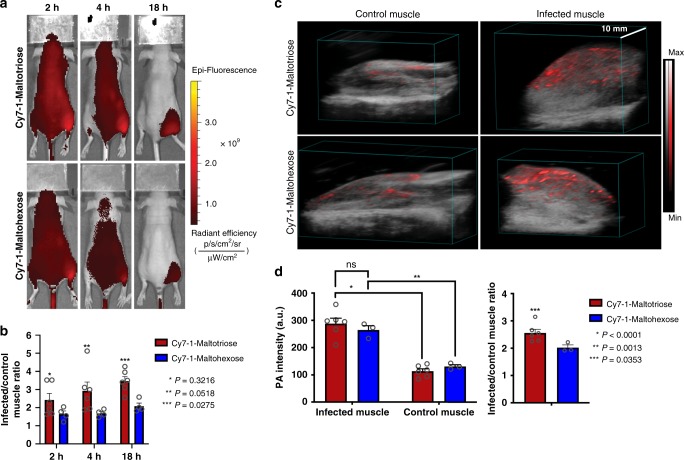
In vivo comparison between Cy7-1-maltotriose and Cy7-1-maltohexose in an *E. coli*-induced myositis murine model. **a** In vivo fluorescence images at 2, 4, and 18 h post-injection of either Cy7-1-maltotriose (top) or Cy7-1-maltohexose (bottom) showing accumulation of both probes in the right thigh muscle (*E. coli*). Qualitatively higher fluorescence signal in the infected muscle (right thigh) when injecting Cy7-1-maltotriose (top) compared to Cy7-1-maltohexose (bottom) is observed. **b** Bar plot representation of ratio of fluorescence in the infected thigh vs control thigh. Higher fluorescence signal in the infected muscle (right thigh) when injecting Cy7-1-maltotriose (*n* = 6) was observed compared to injecting Cy7-1-maltohexose (*n* = 4) and this was significant at 18 h post-injection (*P* < 0.0275). **c** 3D rendered photoacoustic image overlaid on ultrasound image of a mouse 21 h post-injection of Cy7-1-maltotriose (top) and Cy7-1-maltohexose (bottom). Images show infected (right thigh) and control (left thigh) muscle. Evident PA signal is observed in the infected thigh muscle when injecting either compounds while minimal signal is observed in the control thigh muscle. **d** Bar plot representation of the quantified photoacoustic signal intensity in the photoacoustic images acquired 21 h after injection of Cy7-1-maltotriose and Cy7-1-maltohexose. The quantified signal shows significantly higher PA signal in the infected muscle compared to control muscle when injecting either compounds (left). No significant difference in PA signal was observed between the two compounds. Significantly higher infected over the control PA signal ratio was observed when injecting Cy7-1-maltotriose (*n* = 6) compared to Cy7-1-maltohexose (*n* = 3) (*P* < 0.0353) (right). Bar graphs show mean and S.E.M. Statistical analysis was performed using two-way ANOVA. *ns* no statistically significant difference (*P* > 0.05). The source data underlying Fig. 4b and d are provided in a Source Data file.

In the same animal model, a comparison study between the maltotriose and maltohexose derivatives (compound **3a** and **3b**, respectively) was conducted. A higher fluorescence signal in the infected thigh (right) was observed in the mice injected with compound **3a** (Fig. 4a, top). In addition, fluorescence signal in the infected muscle was quantified and normalized to control muscle (Fig. 4b) and showed ~1.5 times higher fluorescence signal in the infected thigh of mice injected with Cy7-1-maltotriose compared to those injected with Cy7-1-maltohexose. In PAI, significantly higher PA intensity in the infected thigh compared to control thigh was observed using either probes (*P* < 0.0013) (Fig. 4c, d, Supplementary Fig. 12). Slightly higher PA intensity in the infected thigh of mice injected with Cy7-1-maltotriose (*n* = 6) was observed compared to ones injected with Cy7-1-maltohexose (*n* = 3), but this difference was not significant (Fig. 4d, left, *P* = 0.7571). However, the PA signal ratio between infected and control muscle was significantly higher for Cy7-1-maltotriose compared to Cy7-1-maltohexose (Fig. 4d, right, *P* = 0.0353).

To further assess the specificity of Cy7-1-maltotriose to bacteria containing the maltodextrin transporter, a similar study where a MalG + LamB mutant of *E. coli* was injected instead in the left thigh. In vivo FLI at 3 and 20 h post-injection also shows a rapid and significantly higher accumulation of Cy7-1-maltotriose in the *E. coli*-infected thigh (right thigh muscle) compared to the left thigh infected with *E. coli* mutation (*n* = 5, Supplementary Fig. 13, *P* < 0.0001).

### In vitro imaging of biomaterial infection

**Fig. 5:**
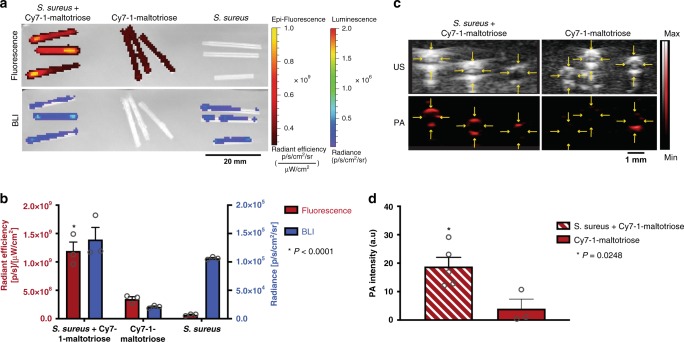
In vitro evaluation of Cy7-1-maltotriose in a *S. aureus*-infected biomaterial model. **a** FLI and BLI images showcasing the presence and lack of *S. aureus* on catheters. High FLI and BLI signals were observed in catheters that were incubated with *S. aureus* followed by Cy7-1-maltotriose (left) compared to sterile catheters that were only incubated with Cy7-1-maltotriose (middle). Similarly, no FLI signal and only BLI signal was observed on catheters that were incubated with *S. aureus* only (right). **b** Total FLI and BLI signals in catheters infected with bioluminescent *S. aureus* and incubated with Cy7-1-maltotriose. Fluorescence quantification was plotted to the left *y*-axes (red) while BLI signal plotted to the right *y*-axes (blue) and showed significantly higher FLI signal in infected catheters compared to sterile catheters post incubation with a solution of Cy7-1-maltotriose (*P* < 0.0001, *n* = 3). **c** Axial US and PA imaging showcasing the presence and lack of *S. aureus* on catheters upon incubation with Cy7-1-maltotriose. An evident PA signal was observed in axial images of catheters incubated with *S. aureus* followed by Cy7-1-maltotriose (bottom left), compared to sterile catheters that were only incubated with Cy7-1-maltotriose (bottom right). Yellow arrows mark the catheter’s outline. **d** Bar plot representation of the quantified photoacoustic signal intensity in the axial photoacoustic images of infected and sterile catheters post incubation with Cy7-1-maltotriose. The infected catheters (*n* = 5) had significantly higher PA signal compared to sterile catheters (*n* = 3) post incubation with Cy7-1-maltotriose (*P* = 0.0248). Bar graphs show mean and S.E.M. Statistical analysis was performed using two-way ANOVA. The source data underlying Fig. 5b and d are provided in a Source Data file.

To examine the ability of our probe to detect bacteria on biomaterials, sterilized catheters were incubated in a solution of 10^6^ CFU of *S. aureus* before incubation in a solution of Cy7-1-maltotriose. Evident FLI and BLI signals were observed on the catheters which were incubated with *S. aureus* solution followed by Cy7-1-maltotriose (Fig. 5a, left). In addition, minimal fluorescence signal in the control catheters which were not incubated with *S. aureus* was observed highlighting the low levels of nonspecific binding of Cy7-1-maltotriose to the catheter (Fig. 5a, b; *P* < 0.0001).

In addition, the catheters which were only incubated with *S. aureus* solution, only showed the BLI signal and no fluorescence signal (Fig. 5a, right). The axial photoacoustic images showed noticeable photoacoustic signals on the surface of catheters incubated with both the bacteria and Cy7-1-maltotriose (Fig. 5c, left). While significantly lower photoacoustic signal was quantified on sterile catheters incubated only with Cy7-1-maltotriose (Fig. 5d; *P* < 0.0248).

### In vivo evaluation in *S. aureus*-infected wound murine model

**Fig. 6:**
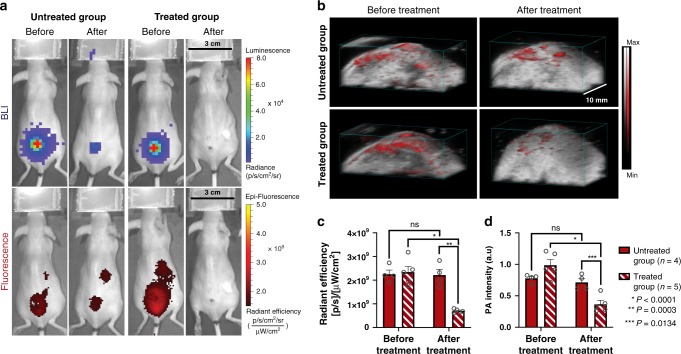
In vivo evaluation of Cy7-1-maltotriose in a *S. aureus* wound infection murine model. **a** BLI and FLI images of mice with a wound infected with 10^6^ CFUs of bioluminescent *S. aureus* 19 h post-injection of Cy7-1-maltotriose without (Untreated Group) and with (Treated Group) treatment with vancomycin for 7 days and before and after treatment. FLI (bottom-Before) shows accumulation of the probe in the *S. aureus* located by BLI (top—Before). In the untreated group (left panel) both BLI and FLI showcases presence of *S. aureus* infection in the wound after 7 days (Untreated Group—After). While in the treated group (right panel), complete disappearance of *S. aureus* infection was observed in the FLI image and confirmed by BLI (Treated Group—After). **b** 3D rendered PA image overlaid on US image of a mouse from Untreated (Top row) and Treated (Bottom row) groups before and after treatment. Images were acquired 20 h post-injection of Cy7-1-maltotriose. Similar to observations in FLI, the Treated group showed lower PA signal post-treatment (bottom right) than that before treatment (bottom left). **c** Total in vivo fluorescence signal in wound infected with 10^6^ CFUs of bioluminescent *S. aureus* 19 h after tail vein injection of Cy7-1-maltotriose. Significant reduction of FLI signal after treating the mice with vancomycin for 7 days in the treated group (*n* = 5, *P* < 0.0001) was observed, while no difference was observed in the untreated group before and after 7 days (*n* = 4). **d** Bar plot representation of the quantified average PA signal intensity in the PA images acquired 20 h after injection of Cy7-1-maltotriose before and after antibiotic treatment. PA quantification data showed a similar trend to that obtained from FLI images. Bar graphs show mean and S.E.M. Statistical analysis was performed using two-way ANOVA. ns no statistically significant difference (*P* > 0.05). The source data underlying Fig. 6c and d are provided in a Source Data file.

In order to mimic a clinically relevant wound infection model, a bioluminescent strain of *S. aureus* (Xen 36) was inoculated into a superficial wound made on the back of the mouse. After confirmation of the presence of bacteria through BLI imaging, FLI and PAI were conducted (Fig. 6, before treatment). Mice were then divided into two groups where one was administered subcutaneously a therapeutic dose of vancomycin twice daily (Treated group, *n* = 5), while the other group was not treated with vancomycin (untreated group, *n* = 4). After 7 days of antibiotic treatment, Cy7-1-maltotriose was administered and imaging completed 20 h post-injection. No BLI nor FLI signal and minimal PAI signal in the wound were observed in the treated group post-treatment (Fig. 6a, b, Treated group—After). While the untreated group showed evident BLI, FLI, and PAI signal in the wound. In addition, a significant decrease in the fluorescence and photoacoustic signal (4.33 ± 0.96 × 10^8^ vs 1.48 ± 0.15 × 10^8^ radiance efficiency and 0.99 ± 0.09 vs 0.37 ± 0.06 a.u. respectively, *P* < 0.0001, *n* = 4) was observed in the images collected post-treatment compared to before treatment (Fig. 6c, d).

**Fig. 7:**
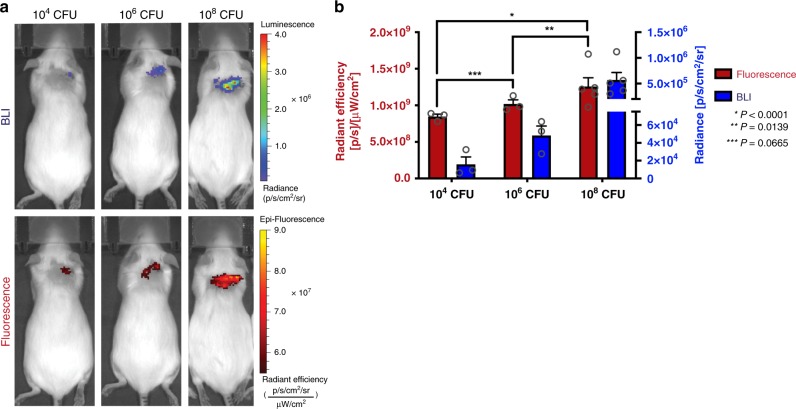
In vivo evaluation of Cy7-1-maltotriose in a *S. aureus* wound infection murine model. **a** FLI and BLI of mice with a wound infected with different amounts of bioluminescent *S. aureus* 22 h post-injection of Cy7-1-maltotriose. FLI images (bottom) show accumulation of the probe in the *S. aureus* in the same area as indicated by BLI (top). **b** Total in vivo FLI and BLI signals in wound infected with 10^4^, 10^6^, and 10^8^ CFUs of bioluminescent *S. aureus* (*n* = 3, 3, and 5, respectively) and 22 h after tail vein injection of Cy7-1-maltotriose. Fluorescence quantification was plotted to the left *y*-axes (red) while BLI signal plotted to the right *y*-axes (blue) and showed increase in FLI signal with increase in BLI signal (*r* = 0.928; *r*^2^ = 0.8612). Bar graphs show mean and S.E.M. Statistical analysis was performed using two-way ANOVA. The source data underlying Fig. 7b are provided in a Source Data file.

In a similar animal model, different amounts of the bioluminescent Xen 36 strain *Staphylococcus aureus* (10^4^, 10^6^, or 10^8^ CFU; *n* = 3, 3, and 5 respectively) were inoculated subcutaneously in a small wound formed on the back of the mice. FLI images 18 h post-injection showed accumulation of Cy7-1-maltotriose in the wound in all three mice groups where the location of the FLI signal directly correlated to that of the BLI signal (Fig. 7a). In addition, a significant increase in the quantified fluorescence signal was observed with an increase in quantified BLI signal (i.e. increase in CFU in the wound) (Fig. 7b).

## Discussion

After evaluating ^18^F-labeled analogs of maltose and maltotriose, our group demonstrated that an ^18^F-6″-labeled maltotriose showed the most optimal pharmacokinetic and pharmacodynamic properties to specifically image bacterial infections^36^. The radio-label, in this case, was at the non-reducing end of the molecule and the addition of ^18^F did not interfere with its internalization through the maltodextrin transporter^36^. While the PET tracer would have extensive applications in a hospital setting, an optical imaging approach would have advantages in an outpatient setting or in the ER for the local evaluation of suspected sites of infection. PAI has the added advantage of increased depth penetration, translation into ultrasound-based settings and the ability to provide valuable real-time anatomical information. We therefore set out to develop a photoacoustic version of the previously reported maltotriose imaging agent. Unfortunately, the ability of the azide-functionalized maltotriose to block ^3^H-maltose uptake in *E. coli* dropped when the azide was functionalized at the 6″ position (Azide-6″-Maltotriose) compared to the 1 position (Azide-1-Maltotriose) (Supplementary Fig. 5). These results confirm previous reports stating the adverse effects of functionalization of the non-reducing end of the maltodextrin on its binding affinity to maltose-binding protein (MBP) which can severely compromise internalization through the maltodextrin transporter^38^. Ning and co-workers^30^ previously reported the development of a fluorescent derivative of maltohexose which was functionalized at the reducing end and showed promising results in optical imaging of bacterial infections. However, Axer et al.^38^ reported that maltotriose could potentially provide a more optimal scaffold in bacterial infection imaging agents compared to other maltodextrins (e.g. maltohexose). This led us to believe that a fluorescent derivative of maltotriose can potentially be a superior bacterial infection imaging agent when functionalized at the reducing end. Accordingly, we sought to functionalize the dye at the anomeric side (reducing end) of maltotriose and decided to also synthesize the maltohexose derivative to properly identify the optimal scaffold for our bacterial infection imaging agent.

We have established a modified synthetic route that produces an azide-1-functionalized maltotriose or maltohexose using an adapted glycosylation procedure^37^ in two high yielding steps (Fig. 1). Developing such an intermediate simplifies the functionalization of the maltotriose and maltohexose scaffold to a variety of signaling agents using copper-free click chemistry or copper catalyzed azide–alkyne reaction. In this work, Cy7 dye was chosen due to its great photo and chemical stability and linked to maltotriose (*n* = 1) or maltohexose (*n* = 4) through azide-DBCO copper-free click chemistry (Fig. 1).

Following synthesis of the probes, it was essential to assess the effects of the azide and Cy7 motifs on the ability of maltotriose and maltohexose to internalize into bacteria. This was assessed by running a competition assay between the tested derivatives and ^3^H-maltose as well as uptake studies (Fig. 2a and b, respectively). Both probes were taken up in a wide variety of Gram-positive and Gram-negative bacterial strains (Fig. 2b). The specificity of the probes for imaging live bacteria that contain the maltodextrin transporter was demonstrated in uptake studies with azide-inactivated *E. coli* and *E. coli* mutants that lack components of the maltodextrin transporter (*P* < 0.0001, *n* = 3) (Fig. 2b, maroon).

Preclinical evaluation of Cy7-1-maltotriose in *E. coli*-induced myositis murine model using FLI and PAI was conducted. FLI was used as the primary tool to assess the specificity and uptake kinetics of the probe in bacterial infections because it allows whole-mouse imaging and provides information that can be compared to previous optical imaging agents for infection. The kinetics of accumulation of the probe were examined and illustrate rapid clearance of Cy7-1-maltotriose through the kidneys. Notably, specific signal accumulation at the site of the infected muscle occurred very rapidly (as early as 1 h), again illustrating the potential utility in field hospitals where quick decisions need to be made (Fig. 3a, b). In addition, photoacoustic images were also able to show a significant difference between the infected and control muscle (Fig. 3c, d, Supplementary Fig. 11). We believe PAI will have several advantages over FLI, as we have discussed in the introduction particularly the enhanced imaging penetration depth. Another potential advantage lies with the instrumentation rapidly being developed^39,40^, and the ease with which these handheld scanners can be integrated into existing ultrasound systems^41^.

It was then important to identify the optimum maltodextrin scaffold to utilize in our bacterial infection imaging agent. A copious amount of work was conducted to assess the difference in internalization kinetics, stability, and retention between maltodextrins in a variety of bacterial strains^38,42–48^. We conducted a comparison between the maltotriose and maltohexose analogs of our imaging agent. Fluorescent maltohexose was previously used by Ning and co-workers^30^^,^^32^ as a fluorescent imaging agent for bacterial infection and showed specificity to bacterial infections in rat models. In vitro competitive and uptake studies looked similar to that of Cy7-1-malotriose (Fig. 2a, b, respectively). A comparison study was then conducted in the *E. coli*-induced myositis murine model. These studies demonstrated the pharmacokinetic advantages of maltotriose over maltohexose in vivo. As shown in Fig. 4, both Cy7-1-maltotriose and Cy7-1-maltohexose showed specific uptake in the infected muscle (right thigh) which is distinguishable from the control muscle (left thigh) (Fig. 4a, b). Eighteen hours post-injection, Cy7-1-maltotriose had 2.6-fold higher fluorescence compared to that of Cy7-1-maltohexose (15.5 ± 5.0 × 10^9^ and 6.02 ± 0.5 × 10^9^ radiance efficiency, respectively; *P* < 0.0275, *n* = 6 and 4, respectively). This can be attributed to the faster clearance of the Cy7-1-maltohexose as compared to Cy7-1-maltotriose from circulation due to higher hydrophilicity (CLogP = −5.8 vs −12.3 for maltotriose and maltohexose, respectively). The in vivo studies match the observations made in our in vitro influx studies where a much faster uptake was observed for the maltotriose derivative (Fig. 2c, *P* < 0.0001).

Stability studies on both compounds in murine, rat, and human plasma as well as phosphate-buffered saline (PBS) over time showed substantial differences in stability between the maltotriose and maltohexose derivatives. Specifically, Cy7-1-maltohexose is shown to rapidly break down into what we hypothesize to be smaller sugar forms in mere minutes in plasma (<2% intact by 2 h in rat and murine and around 10% in humans, Supplementary Fig. 6a, right, Supplementary Fig. 7b). While around 70% of Cy7-1-maltotriose was intact in murine and rat after 2 h and no degradation of maltotriose in human plasma was observed (Supplementary Fig. 6a, left, Supplementary Fig. 7a). In addition, both maltotriose and maltohexose were stable in PBS for up to 24 h (Supplementary Figs. 6a and  7, right). These results directly correlate with previous reports by Axer et al.^38^. In the PA imaging study, slightly higher PA intensity in the infected thigh using Cy7-1-maltotriose (*n* = 6) was observed compared to Cy7-1-maltohexose (*n* = 3) (288.2 vs 264.6 a.u., respectively) yet this difference was not significant (*P* = 0.139). But when looking at the PA intensity ratio of infected over control muscle, a significantly higher ratio using the maltotriose derivative vs maltohexose was observed (2.5 vs 2, respectively, *P* = 0.0353). This further resembles the data shown in the FLI study and is most likely due to the lower sensitivity of PA in detecting Cy7 which is more geared for FLI rather than photoacoustic detection (Supplementary Figs. 8–10).

Evidently, the slower bacterial uptake of the maltohexose derivative, its faster clearance due to its hydrophilicity, and its lower plasma stability provide proof that Cy7-1-maltotriose is the superior bacterial-imaging probe. Further investigation into the exact mechanism of uptake of the two compounds, their metabolism and their ability to be taken up by metabolically inactive bacteria is necessary. In addition, there is some evidence in Enterococcus that maltotriose and maltohexose could be taken up by different transporters at different rates^46^. More experiments are underway such as using CRISPR-based knockouts of individual subunits of the two different transport systems to address this question.

We believe one potential application of Cy7-1-maltotriose is to detect and monitor the treatment of bacterial infections in susceptible sites (i.e. wounds, surgical sites, and medical implants). It is shown that device associated infections account for around 25.6% of all healthcare-associated infections in the United States^6^. For example, it is projected that the total number of knee arthroplasties (TKA) performed per year in the United States will be around three million by 2030 (refs. ^49,50^). One of the leading reasons for TKA failure is due to periprosthetic joint infection (PJI) which can occur in 1–2% of the cases. Unfortunately, current PJI diagnostic tools necessitate sample collection from the prosthetic site and are divided into culture-based tools (ex. peri-implant tissue culture, synovial culture, and histology)^51^ and culture-independent tools (ex. Ibis PLEX-ID technology^52^, MALDI-TOF mass spectroscopy^53^, next-generation sequencing^54^). The latter tools have not yet been adopted in the clinic due to high cost and lack of sufficient validation^55,56^. Thus, a non-invasive tool for PJI diagnostic which does not rely on sampling from the site will be very valuable and allow differentiation from inflammation which is often confused with infections in these situations as they present with similar symptoms. Since *S. aureus* is the most prevalent pathogen in medical device infections and accounts for ~32% of medical device infections^6^, we decided to assess *S. aureus* infections of biomaterials using PAI and FLI upon incubation with Cy7-1-maltotriose. As shown in Fig. 5, Cy7-1-maltotriose is able to differentiate infected from uninfected catheters both using FLI and PAI. This provides the possibility of using PAI to image implants particularly in the joints as well as fracture fixtures to screen for infections.

We then evaluated the ability of our imaging platform in assessing wound infections as well as determining the effectiveness of antibiotic treatment in vivo. *S. aureus* is used in this study since it is the most common cause of SSIs and results in a 5% increase in mortality^8^. Antibiotic treatment reduced the bacterial burden and the probe uptake reflected this decrease and showed significant differences in signal between treated and untreated groups in both FLI and PAI (Fig. 6). In addition, both FLI and PAI were able to distinguish between the treated and untreated groups (Fig. 6c, d, *P* = 0.0003 and 0.0134, respectively) illustrating the utility of this platform in assessing the effectiveness of treatment.

Further FLI evaluation showed an increase in FLI signal (i.e. Cy7-1-maltotriose accumulation) with an increase in CFU of *S. aureus* in the wound (i.e. increase in BLI signal). Wounds infected with as low as 10^4^ CFU were detectable by FLI of Cy7-1-maltotriose and showed a strong correlation with the location of the BLI signal (Fig. 7a, b). In addition, similar imaging done with the *S. aureus* wound model showed that once injected, the probe remains at the infected wound for up to 144 h post-injection, which allows serial imaging without having to administer the probe repeatedly (Supplementary Fig. 14).

In summary, a maltotriose-based infection imaging agent functionalized with an optical dye at the anomeric carbon using copper-free click chemistry was developed. In vitro evaluation showcases the efficacy and specificity of the probe to be taken up by a variety of metabolically active Gram-positive and Gram-negative bacteria. In vivo assessments and in vitro stability studies showed superior performance of the maltotriose-based probe compared to its maltohexose analog. Molecular fluorescence and PAI of bacterial infection was demonstrated and showed the ability to specifically detect bacterial infections in myositis and wound infection models and on biomaterial. Evaluations in *S. aureus*-infected wound model showcased the ability of the probe to detect and differentiate between wounds infected with different amounts of CFUs as well as determine the effectiveness of vancomycin treatment. While the Cy7 dye used in this imaging agent possesses many great characteristics such as in vivo stability and optimum pharmacokinetics when bound to maltotriose, this dye is not approved for clinical use and is more suitable for FLI than PAI. This could explain the higher sensitivity in detecting this probe using fluorescence imaging compared to PAI (Supplementary Figs. 8–10). Hence, we are investigating attachment of a variety of clinically approved dyes as well as dyes more geared for PAI to further enhance its performance. More specifically, the dye needs to have great plasma stability, high photostability, low toxicity and ease of synthesis in a GMP facility. Furthermore, possessing more red-shifted as well as distinctive absorbance characteristics from intrinsic signals (such as hemoglobin and melanin) will further improve imaging depth and signal quantification, respectively. The substitution of the dye may affect the distribution of the imaging agent and a thorough in vivo evaluation of the new probes will be necessary. In addition, while whole-body PAI systems in humans do not exist, small-animal imaging systems have been reported and could provide better means to evaluate the novel agents in a preclinical setting^57,58^. We believe that we have identified the ideal scaffold for an infection imaging agent and with our established synthetic scheme, we can quickly synthesize and clinically translate these new probes.

While PAI is growing to be a promising diagnostic tool, it still possesses several limitations that are being addressed. Namely, the limited field of view and imaging depth constricts its utility in humans to localized imaging (no whole-body imaging). Similar to ultrasound imaging, this tool is highly dependent on the operator’s experience and means to automate this technique will help overcome this issue and allow better and more accurate reproducibility of the images (e.g. Robotic handles and software tools to precisely delineate imaging plane for accurate reproducibility)^16^. Alternatively, three-dimensional (3D) volume scanning should also assist in reducing the dependence of the operator’s experience. Furthermore, several hurdles related to accurate signal quantification (such as differentiating intrinsic signals from imaging agent, reduced accuracy with increased depth and effects of disease progression or tissue healing on PA signal intensity) need to be addressed to conduct molecular PAI of disease^16^. Such limitations can be somewhat mitigated by proper choice of the photoacoustic dye (as mentioned above), choosing the best excitation wavelengths that can assist in differentiating the signal (e.g. background subtraction), the use of models for fluence correction^16^ and other AI tools which can take into account any PA signal intensity changes occurring during structural changes caused by disease progression, inflammation, and healing.

Nevertheless, ultrasound imaging has been widely used to diagnose late-stage infections by visualizing the anatomical changes caused by the spread of infection^9^. Compact and easily transportable PAI systems are being produced with state of the art technologies by many research groups including our own^16,41,59–61^. This makes us believe that the translation of PA for infection imaging can be readily adapted with the pairing of the proper system and imaging agent^16,41,59,60^. The development of a maltotriose-based PA agent accompanied by rapid advances in PAI provides a readily available, non-invasive, cost-effective, real-time imaging tool that can enable early detection of bacterial infections during surgery and injury with high resolution.

## Methods

### Plasma stability studies

Compound **3a** or **3b** (100 µM) was incubated in either PBS (1×), murine, human, or rat plasma at 37 ˚C for up to 24 h. At each time point (0, 2, 4, 10, and 24 h), a 100 µL aliquot of the mixture was taken and added to an ice-cold acetonitrile solution (200 µL) and vortexed for 10 s. Samples were then centrifuged at max speed and the supernatant analyzed on the analytical HPLC column (HPLC method). Compounds **3a** and **3b** had a retention time of 15.25 and 14.65 min, respectively. Monitoring was at 750 nm and the area of any observed peak was assessed in each HPLC chromatogram. Bar plot representation of the compound stability is shown as %intact (Eq. (1)), where

1 $${\mathrm{\% }}\,{\mathrm{{intact}}} = \frac{{{\rm{peak}}\,{\rm{area}}\,{\rm{of}}\,{\rm{compound}}}\,3}{{{\sum }{{\rm{Areas}}\,{\rm{of}}\,{\rm{all}}\,{\rm{observed}}\,{\rm{peaks}}}}} \times 100.$$

### In vitro fluorescence and photoacoustic characterization

Mouse whole blood was collected through cardiac puncture and stored in 1.5 mL heparin–lithium-coated tubes (Eppendorf, cat no.: 022379208) on ice and used within 2 h of collection. A serial dilution of Cy7-1-maltotriose was prepared in either PBS (fluorescence and PAI) or murine whole blood (PAI). In the IVIS spectrum instrument, fluorescence images of 50, 25, 10, 1, 0.5, 0 µM solution of Cy7-1-maltotriose in PBS were collected in a Corning^®^ black clear bottom 96-well plate. While in PAI, the samples were measured in a Vevo PHANTOM imaging chamber, where the samples were injected inside the tubes and imaging conducted using the Vevo3100 LAZR imaging system. Similarly, photoacoustic measurements of 100, 50, 25, 10, 1, and 0 µM solution of Cy7-1-maltotriose in whole blood were collected after the tubes were flushed with heparin sodium solution (1000 USP/mL, NDC 63739-931-14) to avoid coagulation of blood inside the tube.

### Bacterial cultures

*E. coli* was obtained from American Type Culture Collections (ATCC 33456). *E. coli* mutants JW3992-1 (LamB and MalG deficient), JW3995 (LamB and Mal K deficient), and JW1613 (LamB and Mal X-PTS permease deficient) were obtained from *E. coli* Genetic Resources at Yale (Yale University, New Haven, USA). Bioluminescent strain of *Pseudomonas aeruginosa* (Xen 5), *Bacillus subtilis*, and *Staphylococcus aureus* (Xen 36) were obtained from Perkin Elmer.

### Overnight culture conditions

*E. coli* overnight (O/N) culture was prepared by inoculating a colony in Luria-Bertani (LB) broth (3 mL) in an incubator shaker at 37 ˚C. The mutant *E. coli* strains and Xen 36, the bioluminescent strain of *S. aureus*, were grown in LB with 50 µg/mL of Kanamycin. After 16 h, 600 µL of the O/N culture was added to 30 mL of LB in a 200 mL flask and placed in an incubator shaker at 37 ˚C until the bacterial culture reached the log phase (OD_600_ = 0.5). Metabolically inactive *E. coli* was prepared by either treating O/N culture (OD_600_ = 0.5) with sodium azide (10 mM) and incubating for 1 h in an incubator shaker at 37 °C or by heating the culture to 90 °C for 30 min. All cultures were harvested by centrifugation and pellets washed three times with HBSS (1×) before suspending at the concentration of interest.

### Bacteria competition assay

Aliquots of 10^8^ CFU of *E. coli* were first incubated with test compounds (1 mM) for 1 h at 37 °C. After the first incubation, the bacteria culture was centrifuged at 9400 × *g* for 5 min, and the pellet washed with HBSS (1×) three times. Pellets were then resuspended in a solution of ^3^H-maltose (1 µCi in 200 µL HBSS (1×); American Radiolabeled Chemicals, Inc., St. Louis, MO, USA) and incubated for 30 min at 37 °C. Aliquots were then centrifuged and washed with HBSS (1×) three times before lysing in a bacterial lysis buffer (BugBuster, EMD, Billerica MA USA). Activity in bacteria lysates was then counted in a gamma-counter and protein concentration determined using a bicinchoninic acid (BCA) assay (Pierce, Thermo Fisher Scientific). Test compounds included maltose as a positive control, azide-1-maltotriose and Cy7-1-maltotriose. In addition, aliquots incubated with only ^3^H-maltose were also included to assess normal uptake. Results are shown as counts per minute (CPM) normalized to protein content (µg of protein) per sample (*n* = 3 per study).

### Bacteria uptake studies

Aliquots of 10^8^ CFU of *E. coli*, *E. coli* mutants, azide-inactivated *E. coli*, *Pseudomonas aeruginosa*, *Bacillus subtilis,* and *Staphylococcus aureus* (Xen 36) were incubated with the same amount of compound **3a** or **3b** for 1 h at 37 °C. Aliquots were then centrifuged (9400 × *g* for 5 min) and washed with HBSS (1×) three times before lysing in a bacteria lysis buffer (BugBuster for *E. coli* and mutants, EMD; B-PER™ Complete Bacterial Protein Extraction Reagent for the rest of strains, Thermo Scientific™). The fluorescence intensity in bacteria lysates was measured in a SpectraMax GEMINI EM fluorescent plate reader (Molecular Devices, San Jose, CA, USA).

### In vitro influx studies

Aliquots of 10^8^ CFU of *E. coli* were incubated with the same amount of compound **3a** or **3b** (50 µM) at 37 °C. After each time point (30, 60, 240, and 1080 min), aliquots were then centrifuged (9400 × *g* for 5 min) and washed with HBSS (1×) three times before lysing in a bacteria lysis buffer (BugBuster for *E. coli* and mutants, EMD; B-PER™ Complete Bacterial Protein Extraction Reagent for the rest of strains, Thermo Scientific™). The fluorescence intensity in bacteria lysates was measured in a SpectraMax GEMINI EM fluorescent plate reader (Molecular Devices, San Jose, CA, USA).

### Animals and infection models

All animal models and in vivo experiments were approved by the Stanford University Institutional Animal Care and Use Committee.

### *E. coli*-induced myositis murine model

Female *nu/nu* mice 6–7 weeks old were anesthetized by isoflurane inhalation (2–3%). 10^8^ CFU of *E. coli* in 50 µL of HBSS was injected intramuscularly into the right thigh muscle of the mice. As a control, 10^8^ CFU of heat-inactivated *E. coli* or *E. coli* MalG+LamB mutant in 50 µL of HBSS (1×) was injected intramuscularly in the left thigh.

### *Staphylococcus aureus* wound infection murine model

Female CD1 or SKH1-elite mice, 6–8 weeks old were anesthetized by isoflurane inhalation (2–3%). A small wound on the upper back or lower back for the CD1 or SKH1 mice, respectively, was formed using a sharp pair of scissors. *Staphylococcus aureus* in 20 µL saline was inoculated into a small pocket subcutaneously before sealing the wound with Vetbond adhesive (1469SB; 3M, St. Paul, MN, USA).

### Fluorescence imaging in *E. coli*-induced myositis model

Immediately after *E. coli*-induced murine myositis (*n* = 3), Cy7-1-maltotriose (5 nmol in 2% DMSO/saline, 100 µL) was injected via the tail vein. Fluorescence images were captured at 1, 3, 5, and 20 h post-injection of Cy7-1-maltotriose. The fluorescence intensity in the right thigh muscle (*E. coli*) and left thigh muscle (heat-inactivated *E. coli*) were then quantified and analyzed.

### PAI in *E. coli*-induced myositis model

Upon inducing myositis by intramuscular injection of 10^8^ CFU of *E. coli* in the right thigh muscle of nu/nu mice, mice were anesthetized and fixed in the prone position for PAI. PA and US images were then collected using a Vevo2100 LAZR imaging system (Vevo^®^LAZR, VisualSonics, Inc., Canada) with PA-mode and Ultrasound B-mode, respectively. The PA system was connected to an LZ-250 transducer that delivered the nanosecond laser pulses with a wavelength of 700 nm. A cross-sectional two-dimensional (2D) and 3D images of the right and left thigh muscle were acquired, and 3D rendered images presented as colored photoacoustic layer overlaid on top of the ultrasound images were produced using VevoLAB software (VisualSonics, Inc., Canada). Using MATLAB, the total PA signal intensity of the 3D volume was produced and quantified using Fiji software and presented as a.u. PA and US images were collected before (*n* = 5) and 24 h after (*n* = 4) tail vein injection of Cy7-1-maltotriose (5 nmol in 2% DMSO/saline, 100 µL injection).

### Fluorescence imaging comparison between compounds **3a** and **3b**

Immediately after *E. coli*-induced murine myositis, Cy7-1-maltotriose (compound **3a**, *n* = 6) or Cy7-1-maltohexose (compound **3b**, *n* = 4) (5 nmol in 2% DMSO/saline, 100 µL injection) were injected via the tail vein. Fluorescence images were captured at 2, 4, and 18 h post-injection of the probes. The fluorescence intensity in the right thigh muscle (*E. coli*) and left thigh muscle (control) were quantified and data analyzed. The data are presented as the ratio of fluorescence intensity in the right thigh muscle (infected) vs left thigh muscle (control).

### PAI comparison between compounds **3a** and **3b**

After FLI, PA, and US images were collected post-injection of either **3a** (*n* = 6) or **3b** (*n* = 3) using a Vevo2100 LAZR imaging system (Vevo^®^LAZR, VisualSonics, Inc., Canada) with PA-mode and Ultrasound B-mode, respectively. The PA system was connected to an LZ-250 transducer that delivered the nanosecond laser pulses with a wavelength of 700 nm. A cross-sectional 2D and 3D images of the right and left thigh muscle were acquired, and 3D rendered images presented as colored photoacoustic layer overlaid on top of the ultrasound images were produced using VevoLAB software (VisualSonics, Inc., Canada). Using MATLAB, the total PA signal intensity of the 3D volume was produced and quantified using Fiji software and presented as a.u.

### In vivo specificity study to maltodextrin transporter

Myositis in mouse was induced by injecting 10^8^ CFU of *E. coli* and 10^**8**^ CFU of *E. coli* MalG+LamB mutant in the right and left thigh, respectively (*n* = 5). Cy7-1-maltotriose (10 nmol in 2% DMSO/Saline, 200 µL injection) was then injected via the tail vein. Fluorescence images were acquired 3 and 20 h post-injection. After imaging, mice were sacrificed, both right and left thigh muscle collected, and ex vivo fluorescence images acquired.

### In vitro imaging of *S. aureus*-infected biomaterial

Sterile catheters were collected from a BD Insyte™ Autoguard™ BC Shielded IV Catheter (ref: 382544, BD, Franklin Lakes, NJ, USA) and placed in a 10^6^ CFU of *S. aureus* per mL solution in an incubator shaker at 37 °C for 2 h. Catheters were then placed in a Cy7-1-maltotriose solution (50 nmol/mL) and incubated for 37 °C for 1 h before washing by gently dipping in PBS (1×) solution. As a control, sterile catheters that were not exposed to infection or infected catheters that were not incubated with the probe were assessed. BLI and fluorescence images of the catheters were then collected and analyzed. In addition, the catheters were placed inside a 4% agarose phantom and axial ultrasound and photoacoustic images collected using a Vevo2100 LAZR imaging system (Vevo^®^LAZR, VisualSonics, Inc., Toronto, Canada) and irradiation at 700 nm. PA and US images were analyzed using VevoLAB software (VisualSonics, Inc., Toronto, Canada).

### Fluorescence and bioluminescence imaging of treatment study

10^6^ CFU (*n* = 9) of kanamycin-resistant *S. aureus* were inoculated subcutaneously in the wound in SKH1-elite mice. Mice were then administered kanamycin (800 mg/kg) intramuscularly once daily to ensure no other bacterial infections occur. Two days post-surgery, Cy7-1-maltotriose (10 nmol in 2% DMSO/saline, 200 µL injection) was injected via the tail vein and bioluminescence and fluorescence imaging were performed at 19, 45, 69, 93, and 144 h post-injection. Following imaging at 19 h, the mice were divided into untreated (*n* = 4) and treated (*n* = 5) groups where the first group only received kanamycin and the latter was administered vancomycin (110 mg/kg) subcutaneously twice daily in addition to kanamycin. After 7 days of antibiotic treatment, mice were injected again with Cy7-1-maltotriose (10 nmol in 2% DMSO/saline, 200 µL injection) and bioluminescence and fluorescence imaging performed 24 h post-injection.

### PAI of treatment study

Twenty and 25 h post-injection of Cy7-1-maltotriose (before and after treatment respectively), mice were anesthetized and fixed in the prone position for PAI. PA and US images were then collected using a Vevo3100 LAZR imaging system (Vevo^®^LAZR, VisualSonics, Inc., Canada) with PA-mode and B-mode, respectively. The PA system connected to an MX-250 transducer that delivered the nanosecond laser pulses with a wavelength of 700 nm. A cross-sectional 2D and 3D images of the right and left thigh muscle were acquired, and 3D rendered images presented as colored photoacoustic layer overlaid on top of the ultrasound images were produced using VevoLAB software (VisualSonics, Inc., Canada). The whole region of the wound was highlighted using VevoLAB software and average PA signal intensity quantified and presented as a.u.

### Bacterial burden differentiation

10^8^ CFU (*n* = 5), 10^6^ CFU (*n* = 3), and 10^4^ CFU (*n* = 3) of *S. aureus* were inoculated subcutaneously in the wound in CD1 mice. Cy7-1-maltotriose (5 nmol in 2% DMSO/saline, 100 µL injection) was then injected via the tail vein. To ensure no other infections occur, a daily dose of kanamycin (800 mg/kg) was given to the mice intramuscularly. Fluorescence and bioluminescence images were then captured at 5 and 20 h post-injection of the probe. Image analysis was conducted using Living Image^®^ software.

### Reporting summary

Further information on research design is available in the Nature Research Reporting Summary linked to this article.

## Supplementary information

Supplementary Information

Peer Review

Reporting Summary

## Data Availability

Data supporting the findings of this work are available within the paper and its Supplementary Information files. A reporting summary for this article is available as a Supplementary Information file. The datasets generated and analyzed in the current study are available from the corresponding author on request. The source data underlying Figs. 2a–c, 3b, d, 4b, d, 5b, d, 6c, d, and 7b are provided as a Source Data file.

## Source data

Source Data File

## Code availability

The codes that support the findings of this study are available from https://github.com/idanstei/Maltotriose-based-probes-for-fluorescence-and-photoacoustic-imaging-of-bacterial-infections.
