# Supplementary material for: Maltotriose-based probes for fluorescence and photoacoustic imaging of bacterial infections
Source: Nat Commun. 2020 Mar 6;11:1250. doi: 10.1038/s41467-020-14985-8 (PMC7060353; doi:10.1038/s41467-020-14985-8)
Supplement: Supplementary file 1 — Supplementary Information [file 41467_2020_14985_MOESM1_ESM.pdf]

Maltotriose-based probes for fluorescence and photoacoustic imaging of bacterial infections

Zlitni *et al*.

Supplementary Methods

General

Chemicals were purchased from Sigma Aldrich (St. Louis, MO, USA), Biosynth chemistry and biology (Rietlistrasse, Staad, Switzerland), Thermo Fisher Scientific (Waltham, MA, USA) and LumiProbe (Hunt Valley, Maryland, USA) with no further purification. HPLC purification was performed on a Dionex HPLC system (Dionex Corporation, Sunnyvale, CA) equipped with an Ultimate 3000 Pump and Ultimate 3000 RS Variable Wavelength Detector monitoring at 280 and 750 nm wavelengths. Semipreparative HPLC reverse phase column (Phenomenex, Gemini, Hesperia, CA, C_{18}, 5 µm, 10 × 250 mm) eluted at a flow rate of 3 mL/ min. Analytical HPLC was performed on an Agilent system equipped with 1260 Infinity II quaternary pump and 1260 Infinity II diode array detector HS monitoring at 750nm wavelength. Analytical HPLC reverse phase column (Phenomenex, Gemini, Hesperia, CA, C_{18}, 5 µm, 250 × 4.6 mm) eluted at a flow rate of 1 mL/min.

HPLC method

Solvent A = 0.1% trifluoroacetic acid (TFA) in water; Solvent B = 0.1% TFA in acetonitrile: gradient elution, 10% B (0-2 min), 10-100% B (2-20 min), 100% B (20-23 min), 10% B (23-24 min), 10% B (24-26 min). Flash chromatography was conducted on a CombiFlash® Rf+ Lumen system (Teledyne ISCO Inc., Lincoln, NE, USA) equipped with an Evaporative Light Scattering Detector (ELSD detector) and a RediSep Rf Normal-phase Silica gel column (4 gm and 20 gm).

CombiFlash method

Solvent A= Hexane, Solvent B= Ethyl Acetate; 0-40%B (0-5min), 40-45%B (5-27min), 70%B (27-35min). ^{1}H and ^{13}C NMR spectra were performed on an Agilent 400-MR NMR Spectrometer. Electron spray ionization (ESI) mass spectrometry was performed on a Micromass ZQ single quadrupole LC-MS system. Absorption and emission spectra collected on a TECAN SPARK plater reader. Absorbance chromatogram developed from scans collected from 500 to 1000 nm with 1 nm step size while emission chromatogram produced from scanning from 755 to 850 nm after excitation at 750 nm.

In vivo bioluminescence imaging (BLI) was performed using the IVIS Spectrum Imaging System (PerkinElmer, Waltham, MA, USA). The mice were positioned in the instrument after being anesthetized with isoflurane and imaged under medium binning conditions for a suitable exposure time (up to 5 min). Images produced and analyzed using Living Image® software and data expressed as average radiance (p/s/cm^2/sr). *In vivo* fluorescence imaging was performed using the IVIS Spectrum Imaging System (PerkinElmer, Waltham, MA, USA). The mice were anesthetized with isoflurane and imaged in prone position under medium binning conditions for a suitable exposure time (up to 2 min). Images produced and analyzed using Living Image® software and data expressed as average Radiance Efficiency ([p/s]/[µW/cm^2]).

Synthesis (3*R*,4*R*,5*R*,6*R*)-6-(acetoxymethyl)-5-(((3*R*,4*R*,5*R*,6*R*)-3,4-diacetoxy-6-(acetoxymethyl)-5-(((3*R*,4*R*,5*R*,6*R*)-3,4,5-triacetoxy-6-(acetoxymethyl)tetrahydro-2*H*-pyran-2-yl)oxy)tetrahydro-2*H*-pyran-2-yl)oxy)tetrahydro-2*H*-pyran-2,3,4-triyl triacetate (1a) and (2S,3R,4R,5R,6R)-6-(acetoxymethyl)-5-(((3R,4R,5R,6R)-3,4-diacetoxy-6-(acetoxymethyl)-5-(((3R,4R,5R,6R)-3,4-diacetoxy-6-(acetoxymethyl)-5-(((3R,4R,5R,6R)-3,4-diacetoxy-6-(acetoxymethyl)-5-(((3R,4R,5R,6R)-3,4-diacetoxy-6-(acetoxymethyl)-5-(((3R,4R,5R,6R)-3,4,5-triacetoxy-6-(acetoxymethyl)tetrahydro-2H-pyran-2-yl)oxy)tetrahydro-2H-pyran-2-yl)oxy)tetrahydro-2H-pyran-2-yl)oxy)tetrahydro-2H-pyran-2-yl)oxy)tetrahydro-2H-pyran-2-yl)oxy)tetrahydro-2H-pyran-2,3,4-triyl triacetate (1b)

Reaction was modified from previously reported synthesis procedure. [1] Maltotriose (257.2mg, 0.51mmol) or maltohexose (500mg, 0.51 mmol) were dissolved in pyridine (10mL) at room temperature and purged with N_2 gas. When fully dissolved, Ac_2O (5mL) was added and solution was mixed under inert conditions at room temperature for 72hrs. Solvent was then evaporated under vacuum and precipitate was dissolved in EtOAc (100mL). Crude mixture was then washed in Na_2CO_3 1M aq. solution (10mL) × 3, HCl 0.1M aq. solution (10mL) × 3 and brine (10mL) × 3. Organic layer was then collected and solvent dried under vacuum. Off-white precipitate was then dissolved in DCM (2mL), loaded on a silica gel column and purified by flash column chromatography (CombiFlash method) to afford **1a** and **1b** in 95% and 88% yield respectively.

1a ($C_{40}H_{54}O_{27}$): ^{1}H NMR (400 MHz, $CDCl_3$): *δ (ppm)* 6.09 (d, 1H, *J* = 4.0 Hz), 5.62 (d, 1H, *J* = 8.0 Hz), 5.37 (t, 1H, *J*= 8 Hz), 5.30-5.12 (m, 4H), 4.93 (t, 1H, *J* = 8 Hz), 4.90-4.83 (m, 1H), 4.70 (dd, 1H, *J* = 4 Hz and 12 Hz), 4.62-4.57 (m, 1H), 4.35-4.28 (m, 2H), 4.18-4.01 (m, 4H), 3.92-3.73 (m, 6H), 2.09 (s, 1H), 2.03-2.01 (m, 5H), 1.96-1.84 (m, 24H). ESI+ MS m/z 989.37 for [**1a** + Na]; ESI- MS m/z 1011.33 for [**1a** + FA];

1b ($C_{76}H_{102}O_{51}$): ^{1}H NMR (400 MHz, $CDCl_3$): *δ (ppm)* 6.16 (d, 1H, *J* = 4.0 Hz), 5.67 (d, 1H, *J* = 8.0 Hz), 5.45-5.20 (m, 10H), 4.98 (t, 2H, *J* = 12 Hz), 4.90-4.84 (m, 1H), 4.76 (dd, 1H, *J* = 4 Hz and 8 Hz), 4.67-4.63 (m, 4H), 4.41 (d, 4H, *J* = 12 Hz), 4.32-4.10 (m, 9H), 3.97-3.79 (m, 11H), 2.15-1.90 (m, 60H). ESI+ MS m/z 1853.54 for [**1b** + Na]

Synthesis (2*R*,3*R*,4*R*,5*R*)-2-(acetoxymethyl)-6-(((2*R*,3*R*,4*R*,5*R*)-4,5-diacetoxy-2-(acetoxymethyl)-6-(((2*R*,3*R*,4*R*,5*R*)-4,5-diacetoxy-2-(acetoxymethyl)-6-(3-azidopropoxy)tetrahydro-2*H*-pyran-3-yl)oxy)tetrahydro-2*H*-pyran-3-yl)oxy)tetrahydro-2*H*-pyran-3,4,5-triyl triacetate (2a) and (2R,3R,4R,5R)-2-(acetoxymethyl)-6-(((2R,3R,4R,5R)-4,5-diacetoxy-2-(acetoxymethyl)-6-(((2R,3R,4R,5R)-4,5-diacetoxy-2-(acetoxymethyl)-6-(((2R,3R,4R,5R)-4,5-diacetoxy-2-(acetoxymethyl)-6-(((2R,3R,4R,5R)-4,5-diacetoxy-2-(acetoxymethyl)-6-(((2R,3R,4R,5R,6R)-4,5-diacetoxy-2-(acetoxymethyl)-6-(3-azidopropoxy)tetrahydro-2H-pyran-3-yl)oxy)tetrahydro-2H-pyran-3-yl)oxy)tetrahydro-2H-pyran-3-yl)oxy)tetrahydro-2H-pyran-3-yl)oxy)tetrahydro-2H-pyran-3-yl)oxy)tetrahydro-2H-pyran-3,4,5-triyl triacetate (2b)

Compound **1a** (389mg, 0.402mmol) or **1b** (431.5mg, 0.236mmol) were placed in a round bottom flask and purged with N_2 for 10min. The flask was then placed on dry ice and cooled down before adding 3-azido-1-propanol (3eq) (112.09µL, 1.206mmol) and (67.8µL, 0.708mmol) respectively. The mixture was stirred on dry ice and under N_2 for 15min before adding BF_3 (5eq) (257.9µL, 2.01mmol) and (144.9µL, 1.18mmol) respectively. The reaction was stirred for another 2hrs on dry ice and left to warm to room temperature and stirred overnight. Mixture was then quenched by adding TEA (5eq) (282.1µL, 2.01mmol) and (165.6µL, 1.18mmol) respectively and solvent removed under vacuum. Precipitate was then dissolved in EtOAc,

washed with brine three times and purified by flash chromatography (Method 2). Compounds **2a** and **2b** were achieved in 73% and 80% yield as an off-white precipitate. It is worth noting that the product contained some deacetylated product which was also previously reported [2] and the mixture was used in the following step.

2a ($C_{41}H_{57}N_3O_{26}$) ESI+ MS m/z 1030.57 for [**2a** + Na]; ESI- MS m/z 1052.51 for [**2a** + FA]

2b ($C_{77}H_{105}N_3O_{50}$) ESI+ m/z 1894.98 for [**2b** + Na]; ESI- MS m/z 1907.72 for [**2a** + Cl]

Synthesis 1-(6-((6-(1-(3-(((3*R*,4*S*,5*S*,6*R*)-5-(((3*R*,4*S*,5*S*,6*R*)-3,4-dihydroxy-6-(hydroxymethyl)-5-(((3*R*,4*R*,5*S*,6*R*)-3,4,5-trihydroxy-6-(hydroxymethyl)tetrahydro-2*H*-pyran-2-yl)oxy)tetrahydro-2*H*-pyran-2-yl)oxy)-3,4-dihydroxy-6-(hydroxymethyl)tetrahydro-2*H*-pyran-2-yl)oxy)propyl)-1,9-dihydro-8*H*-dibenzo[*b*,*f*][1,2,3]triazolo[4,5-*d*]azocin-8-yl)-6-oxohexyl)amino)-6-oxohexyl)-3,3-dimethyl-2-((*E*)-2-((*E*)-3-(2-((*E*)-1,3,3-trimethylindolin-2-ylidene)ethylidene)cyclohex-1-en-1-yl)vinyl)-3*H*-indol-1-ium (3a) and 1-(6-((6-(1-(3-(((2R,3R,4S,5S,6R)-5-(((3R,4S,5S,6R)-5-(((3R,4S,5S,6R)-5-(((3R,4S,5S,6R)-5-(((3R,4S,5S,6R)-3,4-dihydroxy-6-(hydroxymethyl)-5-(((3R,4R,5S,6R)-3,4,5-trihydroxy-6-(hydroxymethyl)tetrahydro-2H-pyran-2-yl)oxy)tetrahydro-2H-pyran-2-yl)oxy)-3,4-dihydroxy-6-(hydroxymethyl)tetrahydro-2H-pyran-2-yl)oxy)-3,4-dihydroxy-6-(hydroxymethyl)tetrahydro-2H-pyran-2-yl)oxy)-3,4-dihydroxy-6-(hydroxymethyl)tetrahydro-2H-pyran-2-yl)oxy)-3,4-dihydroxy-6-(hydroxymethyl)tetrahydro-2H-pyran-2-yl)oxy)propyl)-1,9-dihydro-8H-dibenzo[b,f][1,2,3]triazolo[4,5-d]azocin-8-yl)-6-oxohexyl)amino)-6-oxohexyl)-3,3-dimethyl-2-((E)-2-((E)-3-(2-((E)-1,3,3-trimethylindolin-2-ylidene)ethylidene)cyclohex-1-en-1-yl)vinyl)-3H-indol-1-ium (3b)

Compound **2a** (30mg, 0.030mmol) or **2b** (60mg, 0.032mmol) and Cy7-DBCO (25mg, 0.028mmol) were dissolved in a 1:1 mixture of DCM:MeOH (6mL) and mixture stirred at room temperature overnight. 25 wt. % in methanol of NaMeOH (2mL) was then added to the crude mixture and stirred at room temperature for 3 hrs before quenching the reaction by adding AcOH (200µL). Solvent was evaporated under vacuum and crude product dissolved in MeOH and

purified by reverse-phase HPLC (HPLC Method) resulting compound **3a** and **3b** in 65% and 60% overall yield respectively.

3a ($C_{79}H_{102}N_7O_{18}$): ^{1}H NMR (400 MHz, $(CD_3)_2SO:D_2O$ (3:1)): δ *(ppm)* 7.61-7.14 (m, 16H), 5.88 (d, 1H, J = 16 Hz), 5.80 (d, 1H, J = 20 Hz), 5.47 (d, 1H, J = 20 Hz), 5.00 (s, 2H), 4.89 (d, 1H, J = 20 Hz), 4.55 (m, 1H), 4.42-4.26 (m, 2H), 4.23-3.98 (m, 19H), 3.76-3.21 (m, 18H), 3.08 (t, 2H, J = 8 Hz), 3.04-3.00 (m, 1H), 2.97-2.90 (m, 1H), 2.88-2.77 (m, 2H), 2.42-2.11 (m, 4H), 2.02-1.98 (m, 2H), 1.94-1.40 (m, 18H), 1.37-1.07 (m, 8H), 0.95-0.71 (m, 3H). MALDI-TOF MS m/z found: 1436.7065, calculated: 1436.7276

3b ($C_{97}H_{132}N_7O_{33}$): ^{1}H NMR (400 MHz, $(CD_3)_2SO$): δ *(ppm)* 7.73-7.19 (m, 19H), 7.09 (s, 1H), 6.67 (s, 1H), 6.17-6.13 (d, 1H, J = 16 Hz), 6.16-6.12 (d, 1H, J = 16 Hz), 5.56 (d, 1H, J = 20 Hz), 5.46-4.70 (m, 25H), 4.65-4.58 (m, 3H), 4.48-4.31 (m, 4H), 4.31-4.20 (m, 1H), 4.17-4.02 (m, 3H), 3.93 (m, 2H), 3.67-3.22 (m, 29H), 3.07 (t, 2H, J = 8 Hz), 2.99-2.84 (m, 3H), 2.38 (m, 2H), 2.28-2.10 (m, 2H), 2.03 (t, 2H, J = 8 Hz), 1.98-1.88 (m, 1H), 1.87-1.6 (m, 14H), 1.59-1.49 (m, 3H), 1.42-1.10 (m, 8H), 0.98-0.85 (m, 3H). MALDI-TOF MS m/z found: 1922.8394, calculated: 1922.8861.

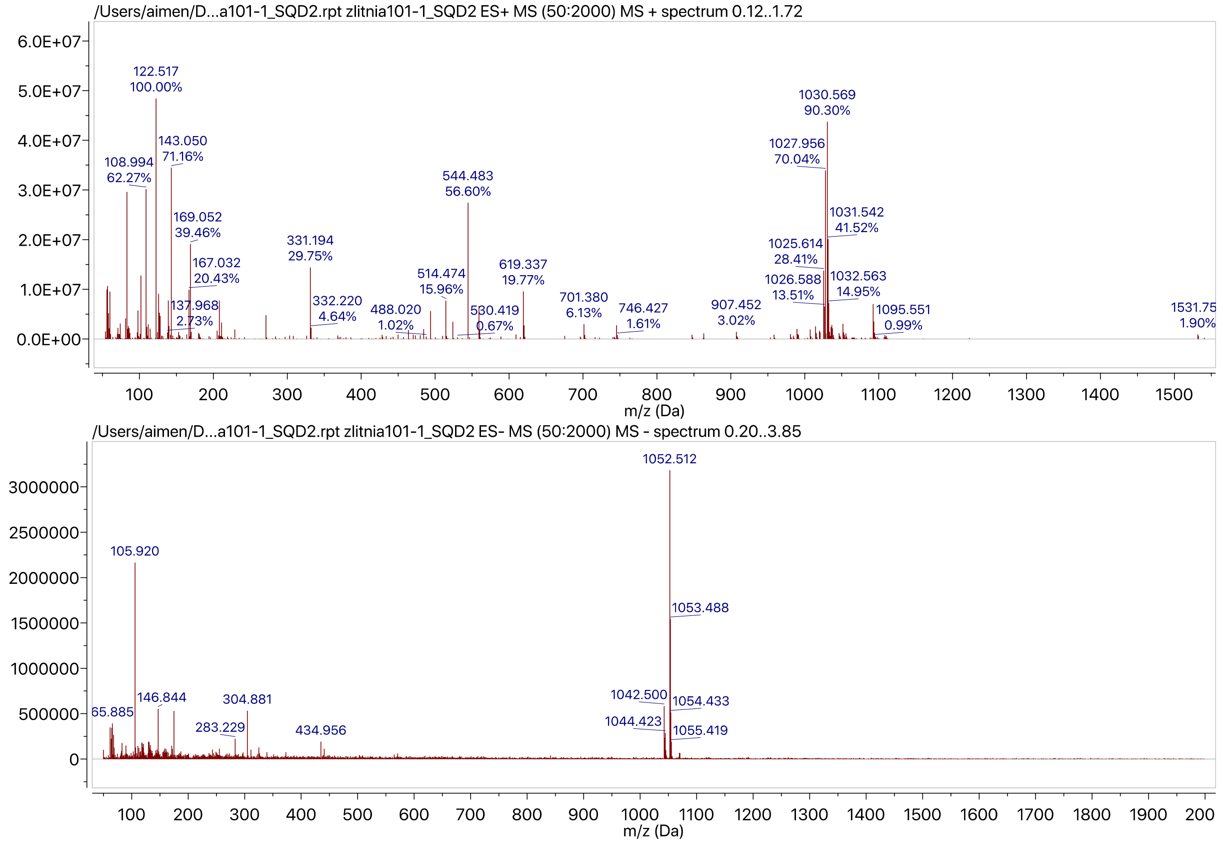

Supplementary Figure 1. ESI-MS of compound 2a.

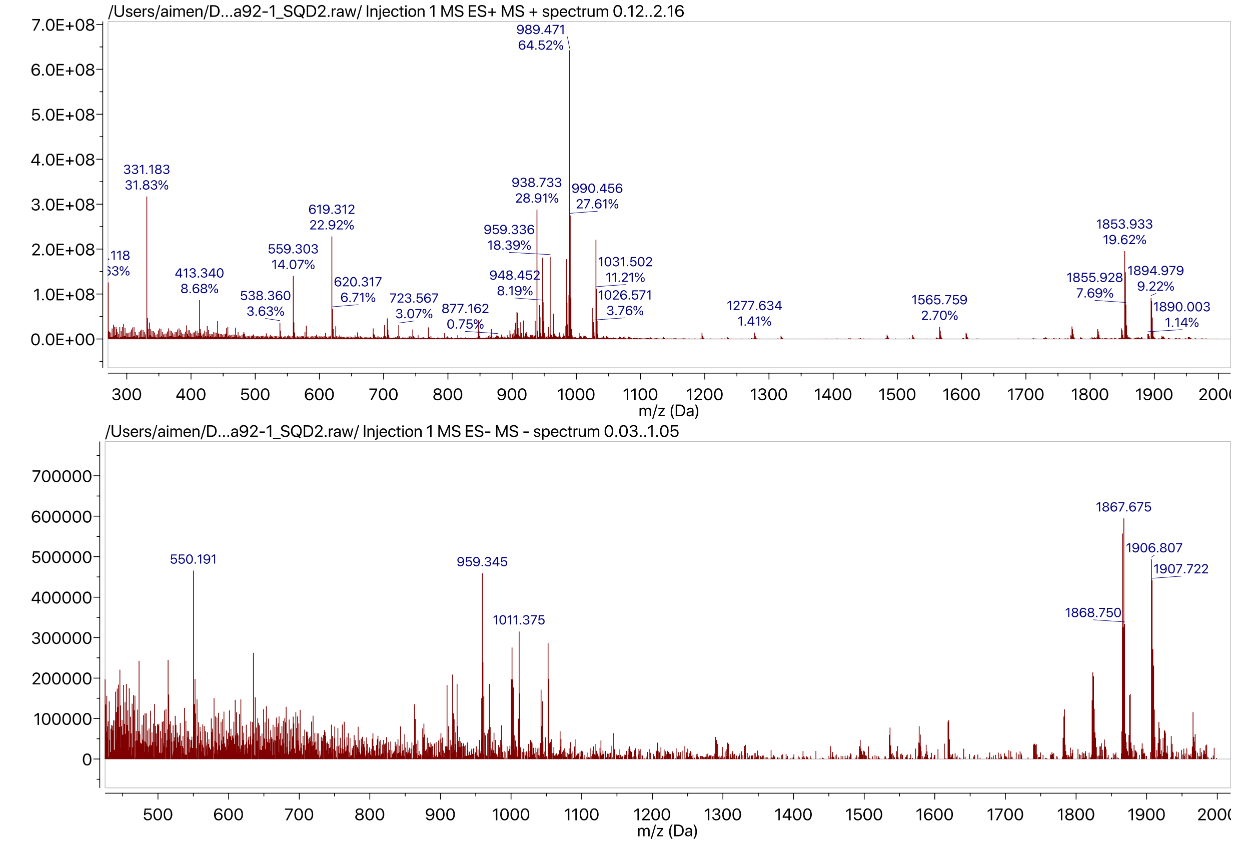

Supplementary Figure 2. ESI-MS of compound 2b.

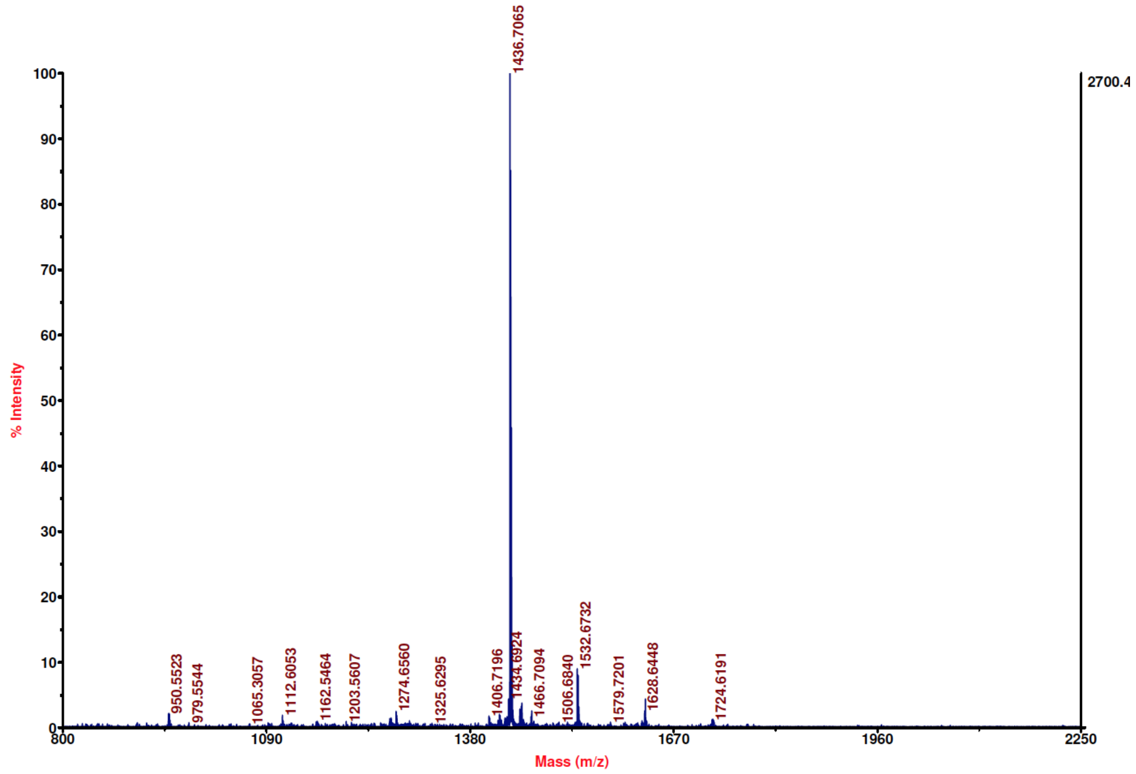

Supplementary Figure 3. MALDI-TOF MS of compound 3a.

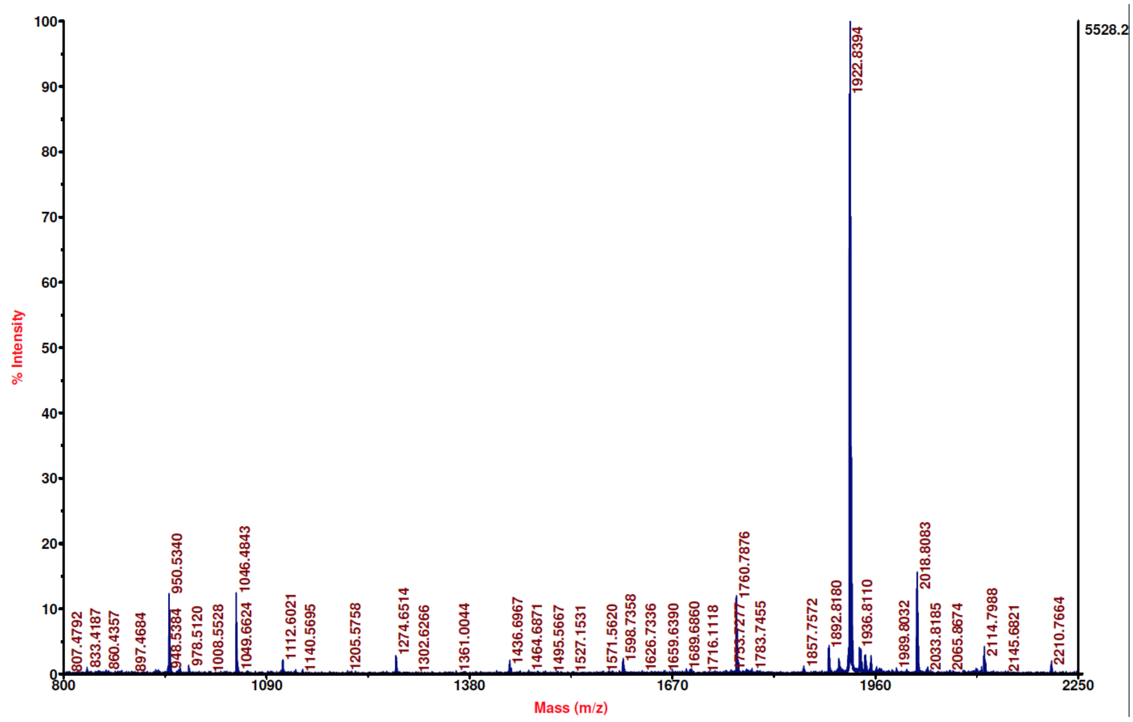

Supplementary Figure 4. MALDI-TOF MS of compound 3b.

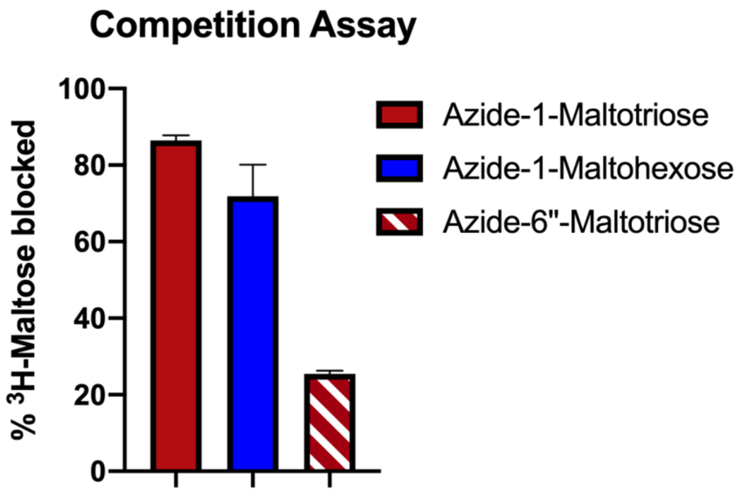

Supplementary Figure 5. Competition binding assay results of Azide functionalized maltotriose at 1 and 6’’ site as well as maltohexose functionalized at 1 position. Bar graphs show mean and S.E.M (n= 3 per compound).

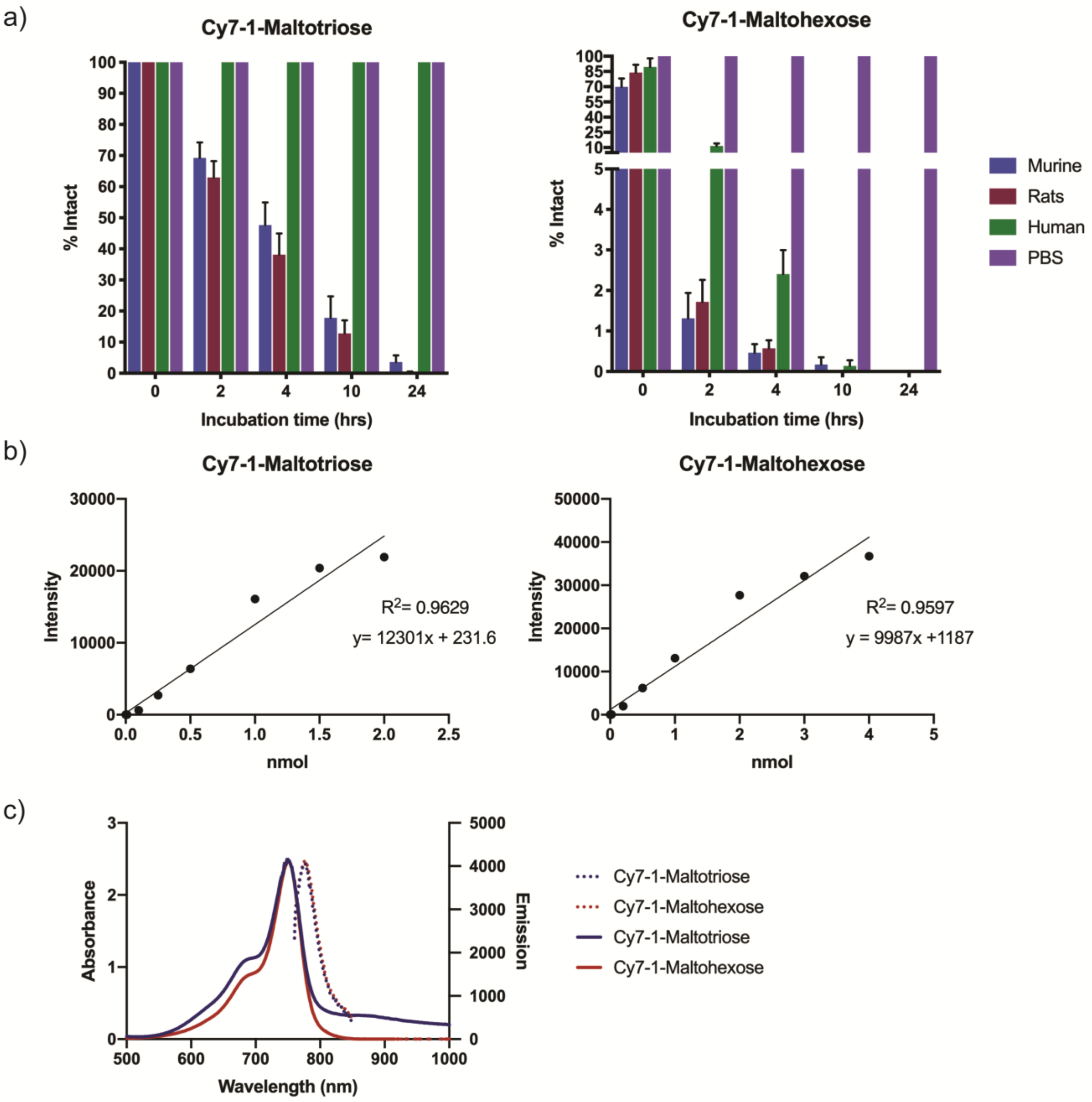

Supplementary Figure 6. *In vitro* characterization of Cy7-1-maltotriose and Cy7-1-maltohexose. a) Murine (n=6), rats (n=4), and human (n=6) plasma and PBS (n=4) stability assessment after incubation at 37ºC for 0, 2, 4, 10 and 24 hrs. At the different time points samples were analyzed on analytical HPLC. Data presented as area under the peak representing the compound of interest over area of all peaks observed in the HPLC trace when monitoring at 750nm. **b)** HPLC detection limit of both Cy7-1-maltotriose (left) and Cy7-1-maltohexose (right). **c)** Absorption (solid line) and emission (dotted line) spectra of Cy7-1-maltotriose (navy) and Cy7-1-maltohexose (maroon). Bar graphs show mean and S.E.M.

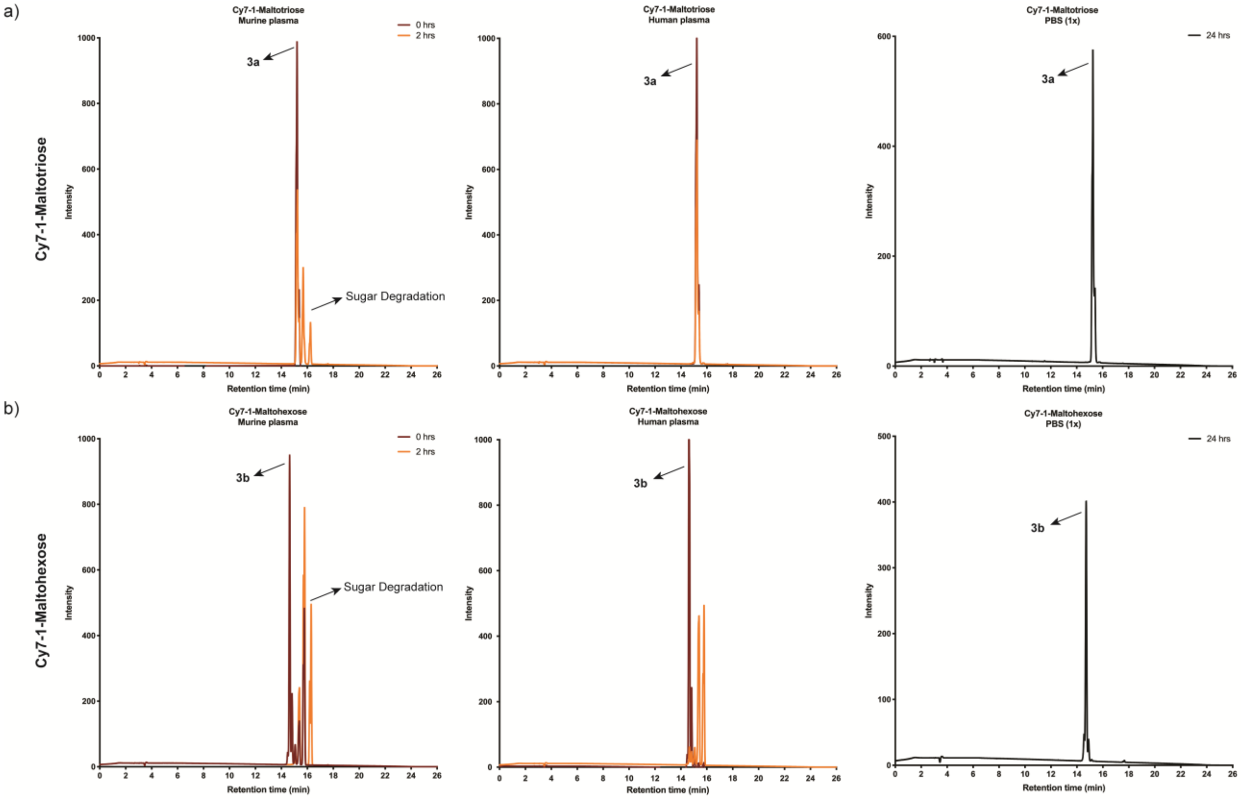

Supplementary Figure 7. HPLC traces of imaging probes after incubation in murine (left) and human (middle) plasma for 0 (maroon) and 2 hrs (orange) as well as after 24 hr incubation in PBS (black). a) Cy7-1-maltotriose. **b)** Cy7-1-maltohexose.

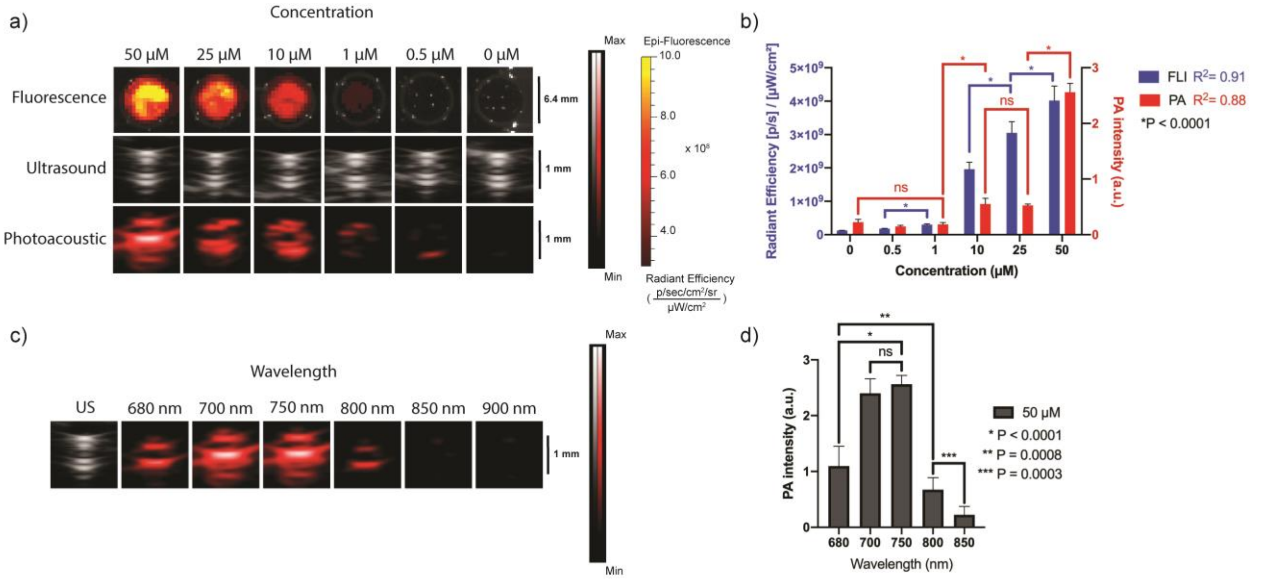

Supplementary Figure 8. *In vitro* characterization of Cy7-1-maltotriose in PBS. a) Fluorescence, ultrasound and photoacoustic images of a phantom containing different concentrations of Cy7-1-maltotriose in PBS. Evident reduction in Fluorescence and PA signal was observed with decrease in concentration. **b)** Plot showing linear correlation between Fluorescence (**blue**) or Photoacoustic (**red**) signal and the concentration of the agent. A detection limit of 1 µM and 10 µM of probe in PBS was determined using Fluorescence and PA imaging respectively. **c)** Photoacoustic imaging of a tube phantom containing 50µM solution of Cy7-1-maltotriose at different excitation wavelengths. **d)** Bar plot representation of the quantified PA signal when exciting at different wavelengths (n=4). Bar graphs show mean and S.E.M. Statistical analysis was performed using two-way ANOVA.

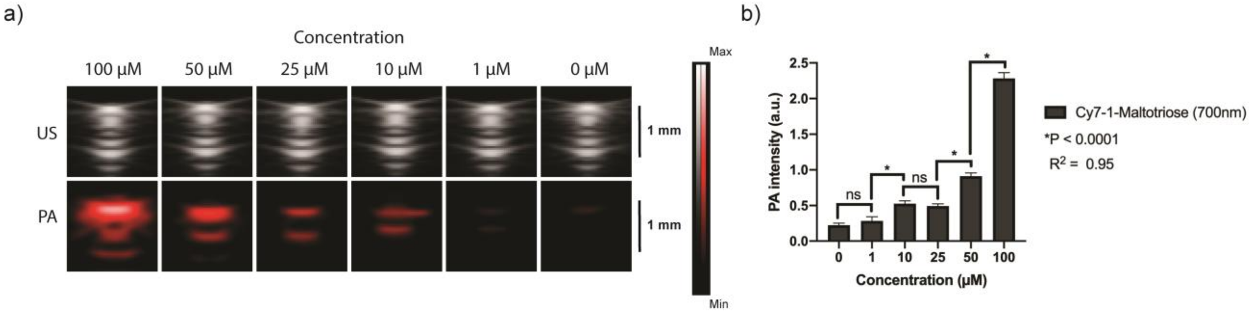

Supplementary Figure 9. *In vitro* PA characterization of Cy7-1-Maltotriose in murine whole blood. a) Ultrasound and photoacoustic images of a phantom containing different concentrations of Cy7-1-maltotriose upon excitation at 700 nm. **b)** Quantified PA intensity of different concentrations of Cy7-1-maltotriose in fresh mouse whole blood (n= 6) showing a 10 μM detection limit in whole blood. ns= no statistically significant difference (P > 0.05). Bar graphs show mean and S.E.M. Statistical analysis was performed using two-way ANOVA.

a)

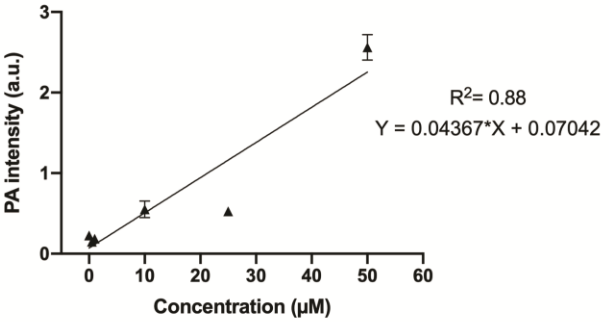

Goodness of Fit	
R square	0.8765
Sy.x	0.3646
Is slope significantly non-zero?	
F	28.39
DFn, DFd	1, 4
P value	0.0060
Deviation from zero?	Significant
Equation	Y = 0.04367*X + 0.07042

b)

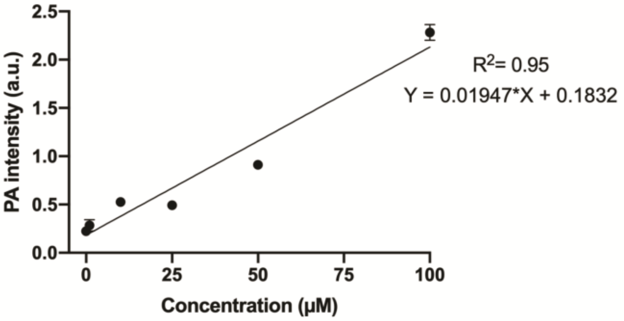

Goodness of Fit	
R square	*0.9472*
Sy.x	*0.1668*
Is slope significantly non-zero?	
F	*610.1*
DFn, DFd	*1, 34*
P value	*<0.0001*
Deviation from zero?	*Significant*
Equation	*Y = 0.01947*X + 0.1832*

Supplementary Figure 10. Linear regression fitting of PAI signal intensity of different concentrations of Cy7-1-maltotriose in a) PBS and b) murine whole blood.

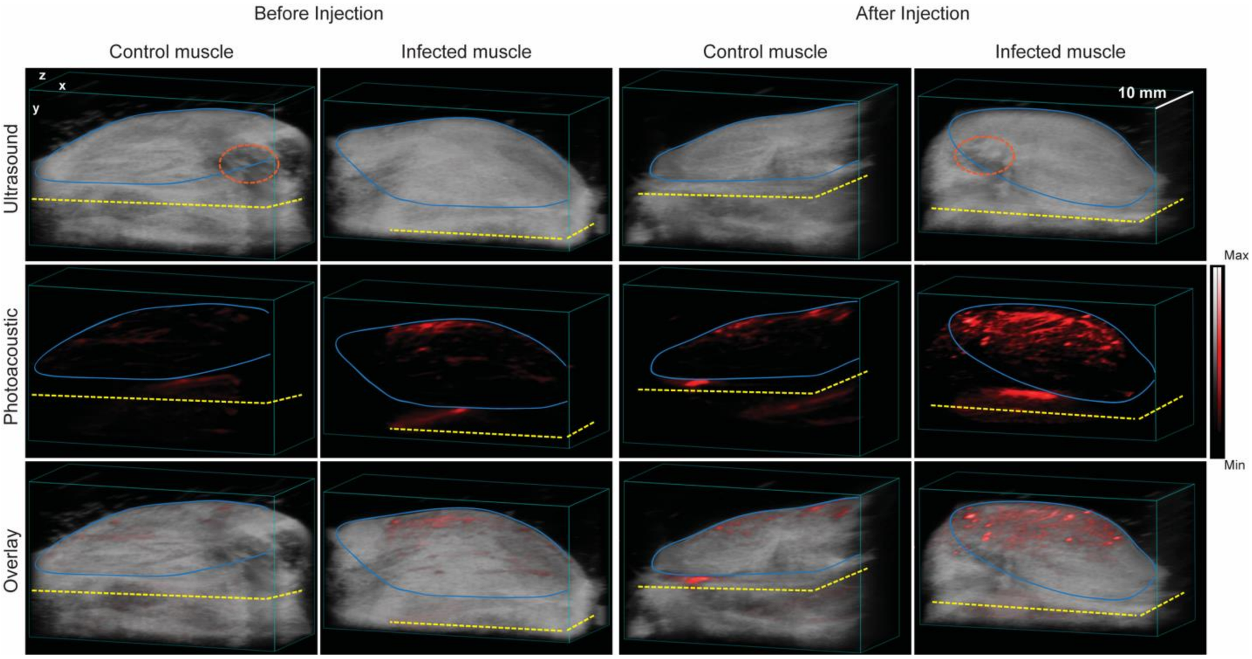

Supplementary Figure 11. 3D rendered ultrasound (top), photoacoustic (middle) and overlay (bottom) image of a mouse's left (control) and right (infected) thigh muscle before and 20 hrs post injection of Cy7-1-maltotriose. Orange: delineates the mouse patella (in xy plane); Blue: thigh muscle (Region of interest (ROI) in xy plane); Yellow: rubber padding placed under the thigh.

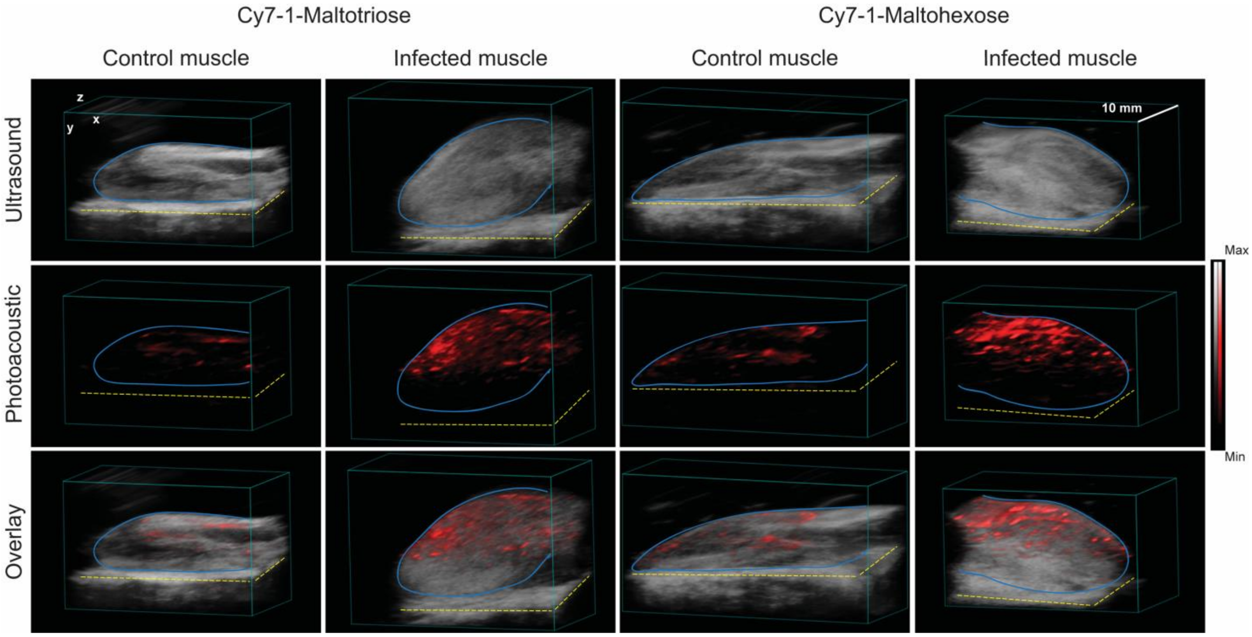

Supplementary Figure 12. 3D rendered ultrasound (top), photoacoustic (middle) and overlay (bottom) image of a mouse's left (control) and right (infected) thigh muscle 21 hrs post injection of Cy7-1-maltotriose (left panel) and Cy7-1-maltohexose (right panel). Blue: thigh muscle (Region of interest (ROI) in xy plane); Yellow: rubber padding placed under the thigh.

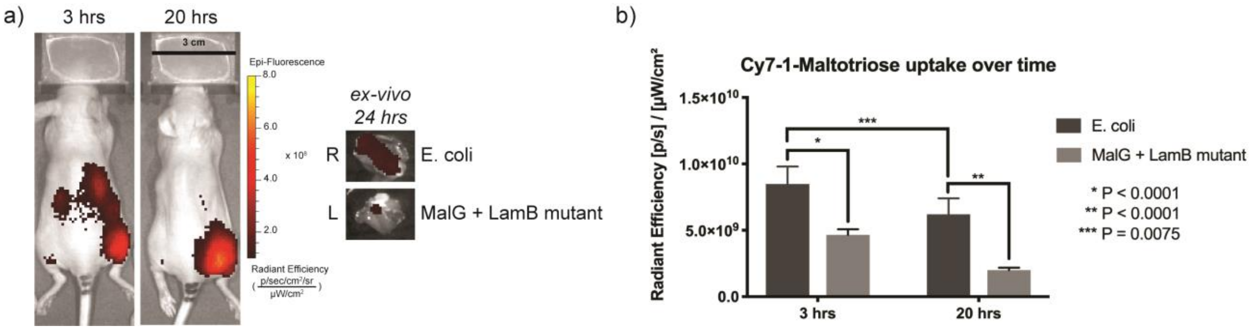

Supplementary Figure 13. *In vivo* validation of Cy7-1-maltotriose in an *E. coli*- and *E. coli* mutation-induced myositis murine model. a) Left: FLI shows accumulation of Cy7-1-maltotriose in *E. coli*-infected thigh muscle at 3 and 20 hrs post systemic injection (right thigh muscle). No evident accumulation of the agent in thigh muscle injected with 10^8 CFUs of *E. coli* MalG + LamB mutant (left thigh muscle). Right: *Ex-vivo* FLI of right and thigh muscle post excision. Image shows higher FLI signal in thigh muscle infected with E.coli compared to *E. coli* mutant. **b)** Bar-plot representation of quantified FLI signal in right and left thigh muscle at 3 and 20 hrs post probe injection. Significantly higher fluorescence signal was found in muscle infected with *E. coli* (right thigh) compared to muscle infected with *E. coli* mutant (left thigh) at both timepoints (n=5, P < 0.0001). Bar graphs show mean and S.E.M. Statistical analysis was performed using two-way ANOVA.

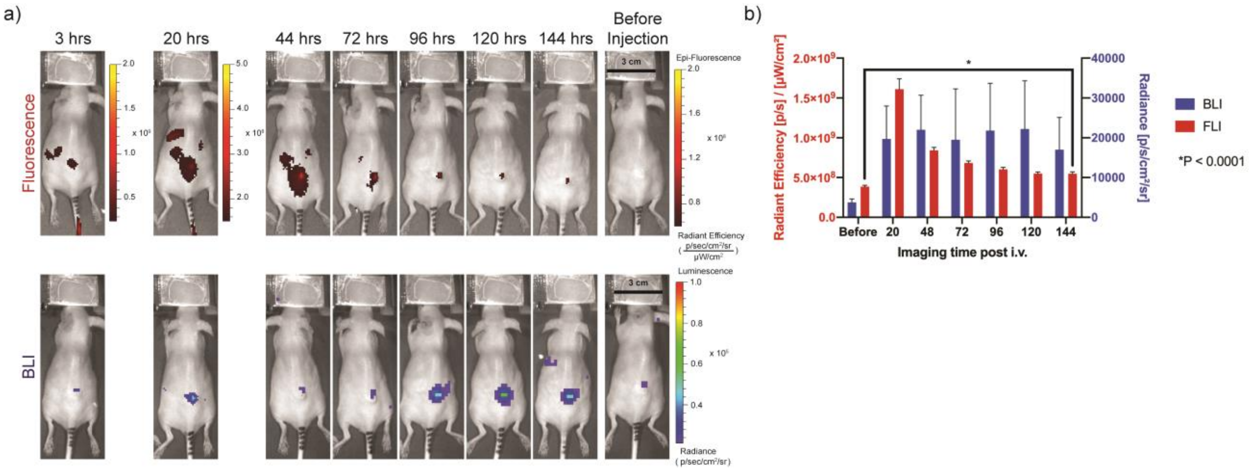

Supplementary Figure 14. *In vivo* evaluation of Cy7-1-maltotriose in a *S. aureus*-infected wound in murine model. a) FLI and BLI images of mice with wound infected with 10^5 CFUs of S. *aureus* collected before and at 3, 20, 44, 72, 96, 120 and 144 hrs post injection of Cy7-1-maltotriose (10 nmol, 200µL injection). Cy7-1-maltotriose was taken up and retained in *S. aureus* for up to 144 hrs. **b)** Total *in vivo* BLI (blue, right y-axis) and FLI signal (red, left y-axis) in wound infected with 10^5 CFUs of *S. aureus* when imaged before and at 20, 44, 72, 96, 120 and 144 hrs post injection of Cy7-1-maltotriose (n=5). Even at 144hrs, significantly higher FLI signal was observed compared to signal before injecting the probe ($P < 0.0001$). The FLI image at 3 and 20 hrs are shown under a different scale (shown beside the image), while the rest of the BLI and FLI images are shown under the same scale (shown on the far right). Bar graphs show mean and S.E.M. Statistical analysis was performed using two-way ANOVA.

Supplementary References

1. Ning, X. *et al.* Maltodextrin-based imaging probes detect bacteria in vivo with high sensitivity and specificity. *Nat Mater* **10,** 602–607 (2011).
2. Khamsi, J., Ashmus, R. A., Schocker, N. S. & Michael, K. A high-yielding synthesis of allyl glycosides from peracetylated glycosyl donors. *Carbohydrate Research* **357,** 147–150 (2012).
