# Supplementary material for: Maltotriose-based probes for fluorescence and photoacoustic imaging of bacterial infections
Source: Nat Commun. 2020 Mar 6;11:1250. doi: 10.1038/s41467-020-14985-8 (PMC7060353; doi:10.1038/s41467-020-14985-8)
Supplement: Supplementary file 2 — Peer Review [file 41467_2020_14985_MOESM2_ESM.pdf]

Reviewers' comments:

Reviewer #1 (Remarks to the Author):

This manuscript focusses on imaging bacteria with maltotriose conjugated to Cy7.1 . The synthesis of maltotriose-Cy7.1 is presented and in vivo studies are performed demonstrating that it can image bacterial infections via fluorescence imaging and photoacoustic imaging. Two animal models of infection are investigated, (1) a myositis infection model with E.coli and a wound infection model with S Aureus. The main finding of this manuscript is that photoacoustic imaging can be used to image bacterial infections. Maltodextrin targeting of bacteria is now well established, and there are about 7 publications demonstrating that maltodextrin targeting is effective at imaging bacterial infections. Consequently, this manuscript does not make a significant contribution to the maltodextrin targeting literature and is below the caliber of papers published in Nature Communications.
Bacteria have been imaged in vivo by fluorescence, PET and SPECT via either maltohexaose or maltotriose based ligands, and demonstrating that maltodextrin targeting can image bacteria by photoacoustic imaging is an incremental advance over the previous literature. In addition, the fluorophores used for photoacoustic imaging, in this manuscript, are almost identical to the previously published fluorescent maltodextrin targeting ligands. For example, Cy7 conjugated to maltohexose has been used in two previous papers to image bacterial infections (Ning et al 2011 Nature Materials and Takemiya et al 2018 JACC Cardiovascular Imaging). The maltotriose targeting ligand has also already been disclosed in at least two previous publications, one for PET and the other for SPECT (Axer et al ChemMedChem 2018, and others published by the authors).
The animal work presented here is also not new enough to warrant publication in Nature Communications. E.coli muscle infections have already been imaged in vivo, via targeting of the maltodextrin transporter, using maltohexose conjugated to Cy7 (Ning et al 2011 Nature Materials), and S Aureus implant infections have also been imaged by maltohexose conjugated to Cy7 (Takemiya et al 2018 JACC Cardiovascular Imaging). The Takemiya paper is not referenced in this paper, and needs to be discussed, given its overlap with the work presented here. On a similar note, the introduction is misleading, and does not appropriately discuss the prior literature on bacterial targeting. In particular, previous work with the maltodextrin targeting ligands maltohexose and maltotriose need to be fully discussed in the introduction, so that readers can appropriately understand the contribution of this work.

Reviewer #2 (Remarks to the Author):

The authors reported a new fluorescent derivative of maltotriose as a bacterial infection imaging agent. They demonstrated its performance using both fluorescence and photoacoustic imaging. Some technical issues regarding photoacoustic imaging should be addressed.
1. It would be helpful if the authors could provide the absorption spectrum of the developed imaging agent. What is the estimated molar concentration of the imaging agent in the infected tissues in vivo?
2. Although hemoglobin has a smaller molar absorption coefficient in the near-infrared light region than visible light region, its high concentration in living tissues can still lead to strong photoacoustic signals. Have the authors compared the photoacoustic signals from the imaging agents (at a similar concentration for in vivo cases) with whole blood at the imaging wavelength of 700 nm?
3. It seems that the optoacoustic signals of blood (or other endogenous chromophores) are not negligible (Fig. 3c, before injection). The detected optoacoustic signals (post-injection) are the integrated signals from both imaging agents and the endogenous chromophores. It is better to add at least one more imaging wavelength for this experiment; then the authors can quantitatively analyze the contributions from the imaging agents and blood.
4. It will be more readable if the authors can add scale bars to both the fluorescence and

photoacoustic images.

Reviewer #3 (Remarks to the Author):

Drug-resistance to antibiotics is a rapidly growing problem, and early detection of this problem is fundamentally important. With this in mind, this paper is highly relevant, and the topic could be of interest to many readers.

The authors report a fluorescent derivative of maltodextrin, Cy7-1-maltotriose for fluorescent and optoacoustic imaging and illustrate its application in the imaging of bacterial infections. The authors present good data, testing models included bacterial cells, tissue culture and mouse models. The aim is for a more local evaluation of infected sites. 18F and fluorescent analogs of maltose and maltotriose were previously shown to specifically image bacterial infections. Optoacoustic imaging presents an attractive feature over optical imaging modalities. The manuscript is well written and constructed thoughtfully. The design of dual modality sensor based on sugars for specifically imaging bacteria should usher new developments in this arena.

However, the overall results are in part underdeveloped with respect to the optoacoustic bioimaging. Furthermore, the review of the literature is somewhat incomplete, since there are already a few optoacoustic imaging technologies for bacterial infections in vivo that exist, none of which were discussed or cited.

Specific comments include:
1. "To the best of our knowledge, this is the first demonstration of molecular PAI of bacterial infections in living mice. " –Please see the examples of previous work listed below. The following works should have been acknowledged. Strong arguments for the advantages of Cy-7-1-maltotriose over the already published agents should be discussed.
- Violacein as a genetically-controlled, enzymatically amplified and photobleaching-resistant chromophore for optoacoustic bacterial imaging", Scientific Reports 5,11048 (2015).
- Pathological-Condition-Driven Construction of Supramolecular Nanoassemblies for Bacterial Infection Detection, Advanced Materials, 2015, 28, issue 2, 254-262
- "A purpurin-peptide derivative for selective killing of Gram-positive bacteria via insertion into cell membrane", J. Mater. Chem. B, 2016,4, 4855-4861
- "A Gold/Silver Hybrid Nanoparticle for Treatment and Photoacoustic Imaging of Bacterial Infection", ACS Nano, 2018, 12 (6), pp 5615–5625
- "In vivo photoacoustic difference-spectra imaging of bacteria using photoswitchable chromoproteins", J. of Biomedical Optics, 23(10), 106006 (2018).
- https://www.visualsonics.com/resource/featured-images/imaging-bacterial-infection

2. The authors set out to synthesize a probe (Line 232) for optoacoustic imaging at greater depths. However, in Fig 3c. the scale bar is missing. At which depths were the authors able to image and still see differences in the signal? It is a challenge to see the signal differences in Fig. 3c between the control muscle and the infected muscle. Would a 3D volume reconstruction best present the data?

3. Optoacoustic imaging shown were carried out 20 h post injection of Cy7-1-maltotriose. Stability studies in serum and 2% DMSO/saline is highly recommended.

4. "Fluorescence and Photoacoustic imaging…" is the title and features arguments for optoacoustic imaging throughout the entire manuscript (line 233, 344 etc.). The paper requires more optoacoustic validation of the probe. So far, in vivo data are only shown once in Fig. 3c. Fluorescence data shown in Fig. 4. is great, but parallel acquisition should also be performed in the optoacoustic system.

5. While the specificity of both agents was tested in vitro, this could also be carried out in vivo. Blocking studies are highly recommended in this case as a negative control. This would also further validate Fig. 3c. Does the fluorescence and optoacoustic data validate each other? It would be useful to repeat Fig 2b in an optoacoustic imaging setting.

6. "Wounds infected with as low as 104 CFU were detectable by fluorescence imaging of Cy7-1-maltotriose." Which is great but what is the detectability in optoacoustic imaging system?

7. Line 37/38: "This will likely greatly enhance early detection and diagnosis of bacterial infections"- the authors should carefully reconsider or further discuss limitations of the sensor. Agents such as maltodextrins are metabolite based, its accumulation is very much dependent on the metabolic state of the bacteria. How close does the in vivo model of 108 bacteria injected mimic the real scenario for detectability?

8. Fig. 5c is missing a scale bar. It is unclear as to what amount/concentration/volume of Cy7-1-maltotriose was used for the experiment. 5 nmol? If that's the case, that's roughly 1 μM (assuming 200 μL i.v.) which does not seem very much in order to acquire decent optoacoustic imaging results. This would explain fig. 3c.

9. The authors compared the fluorescence of Cy7-1-maltotriose and Cy7-1-maltohexose, the added value would be to show the corresponding optoacoustic data.

10. Citation 21 and 22 need to be fixed.

Point-by-Point Response to Reviewer Comments

Manuscript ID: NCOMMS-18-34556

Title: Fluorescence and Photoacoustic Imaging of Bacterial Infections, Towards a New Generation of Maltotriose-based Probes

Summary of Response:

We would like to thank the editor and all the reviewers for their excellent comments and suggestions. In response, we have conducted multiple new experiments and believe that these have significantly improved the quality of our manuscript. Below is a summary of the key new data we have added to the paper.

- Present new data comparing stability of Cy7-1-maltotriose and Cy7-1-maltohexose in murine, rat and human plasma and in PBS over time
- *In vivo* specificity assessment of Cy7-1-maltotriose in a murine myositis model infected with *E. coli* versus mutated *E. coli*
- Provide substantial new photoacoustic imaging data
    - Comparison between Cy7-1-maltotriose and Cy7-1-maltohexose
    - Wound infection PA imaging and antibiotic treatment effectiveness monitoring
- New *in vitro* characterizations of the imaging agent

Reviewer #1

Reviewer #1 (Remarks to the Author):

This manuscript focusses on imaging bacteria with maltotriose conjugated to Cy7.1 . The synthesis of maltotriose-Cy7.1 is presented and in vivo studies are performed demonstrating that it can image bacterial infections via fluorescence imaging and photoacoustic imaging. Two animal models of infection are investigated, (1) a myositis infection model with E.coli and a wound infection model with S Aureus. The main finding of this manuscript is that photoacoustic imaging can be used to image bacterial infections. Maltodextrin targeting of bacteria is now well established, and there are about 7 publications demonstrating that maltodextrin targeting is effective at imaging bacterial infections. Consequently, this manuscript does not make a significant contribution to the maltodextrin targeting literature and is below the caliber of papers published in Nature Communications.

Bacteria have been imaged in vivo by fluorescence, PET and SPECT via either maltohexaose or maltotriose based ligands, and demonstrating that maltodextrin targeting can image bacteria by photoacoustic imaging is an incremental advance over the previous literature. In addition, the fluorophores used for photoacoustic imaging, in this manuscript, are almost identical to the previously published fluorescent maltodextrin targeting ligands. For example, Cy7 conjugated to maltohexose has been used in two previous papers to image bacterial infections (Ning et al 2011 Nature Materials and Takemiya et al 2018 JACC Cardiovascular Imaging). The maltotriose targeting ligand has also already been disclosed in at least two previous publications, one for PET and the other for SPECT (Axer et al ChemMedChem 2018, and others published by the authors).

The animal work presented here is also not new enough to warrant publication in Nature Communications. E.coli muscle infections have already been imaged in vivo, via targeting of the maltodextrin transporter, using maltohexose conjugated to Cy7 (Ning et al 2011 Nature Materials), and S Aureus implant infections have also been imaged by maltohexose conjugated to Cy7 (Takemiya et al 2018 JACC Cardiovascular Imaging). The Takemiya paper is not referenced in this paper, and needs to be discussed, given its overlap with the work presented here. On a similar note, the introduction is misleading, and does not appropriately discuss the prior literature on bacterial targeting. In particular, previous work with the maltodextrin targeting

ligands maltohexose and maltotriose need to be fully discussed in the introduction, so that readers can appropriately understand the contribution of this work.

Summary of response:

We respectfully disagree with the points stated by the reviewer. Although it would appear that our work may be incremental, a deeper dive into the literature and the key differences help to elucidate why our work is significant and novel and an important contribution to the literature.

To simplify the discussion please see below a table summarizing the structures of the reported maltodextrin-based small molecule imaging agents (Table1*). Firstly, as seen in Figure 1*, Cy7 dye differs in structure, name, absorbance and site of functionalization to IR786 dye used by other groups[1,2] and claiming the dyes to be the same is not accurate.

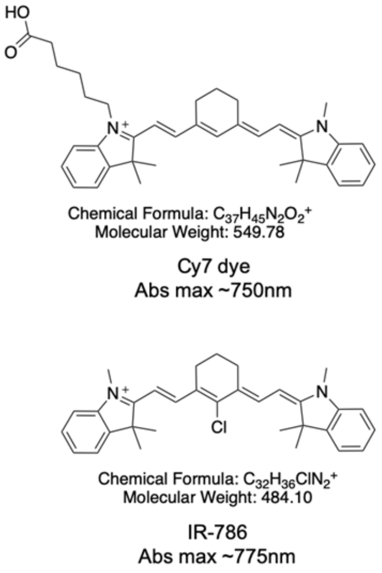

Figure 1*. Structure, formula and reported maximum absorbance of Cy7 dye used in our study and IR-786 dye used by Ning and coworkers.

As to the previous reports on maltotriose, other than the work reported by our lab [3], no *in vitro* nor *in vivo* evaluation of maltotriose in bacterial imaging was conducted. The work by Axer and coworkers, which is referred to by the reviewer, not only discusses a completely different imaging application (single photon emission computed tomography (SPECT) imaging), but used a **maltohexose**-based SPECT imaging agent in their *in vitro* and *in vivo* evaluations (Table 1* - $[^{99m}Tc]$MB1143). In addition, their reported **maltotriose** derivative (Table 1* - $[^{18}F]$AAX21) is functionalized on **the non-reducing end** and was only used in their ***in vitro*** plasma stability studies (with **no *in vitro* nor *in vivo* binding studies performed**)[4]. We need to further reiterate that although Axer and coworkers are one of the first to report the potential advantages of

maltotriose over maltohexose, their plasma stability studies were conducted on derivatives that do not bind to bacteria (due to functionalization at the non-reducing end). However, our work is the first in which a direct comparison between the maltotriose-based and maltohexose-based bacterial imaging agent was conducted *in vitro* (competition and binding assays, influx assay, plasma stability studies) and *in vivo* (FLI and PAI murine studies).

Table 1*. Table summary of name and structure of relevant maltodextrin-based imaging agents

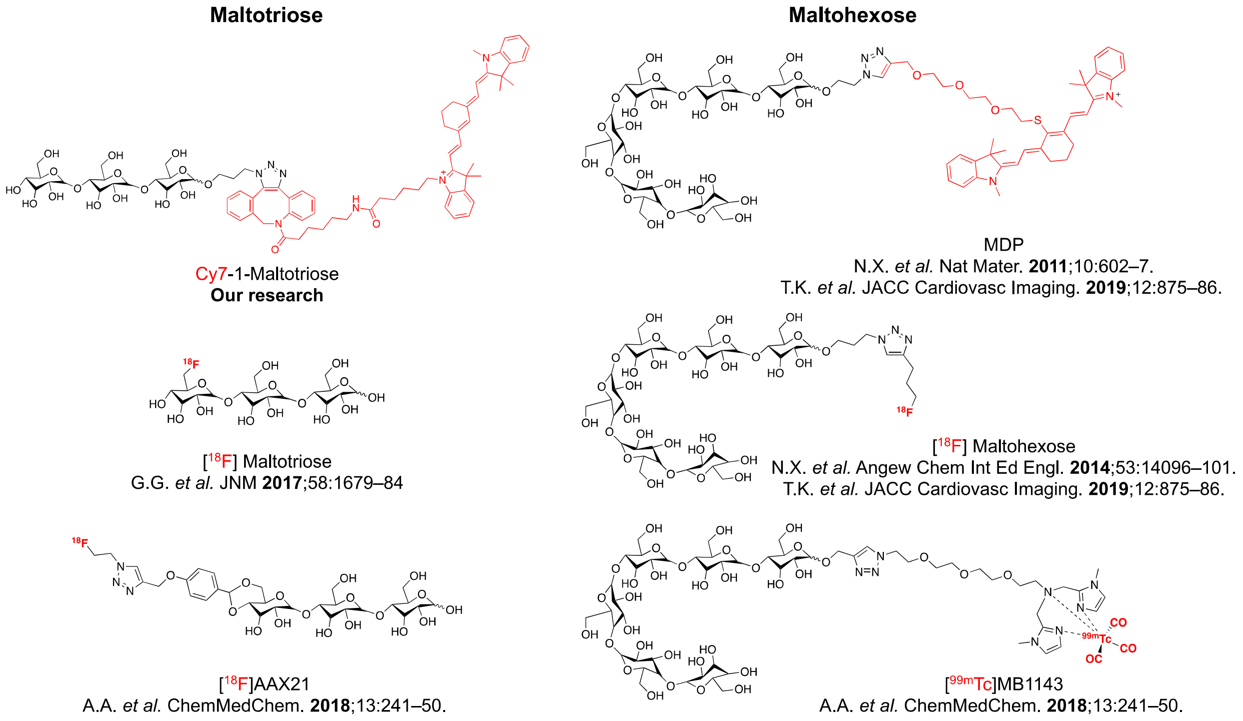

As we mentioned in the introduction, our group investigated the use of maltotriose as a scaffold for a PET-based bacterial infection imaging agent. Our reports here not only highlight a completely different applications of maltotriose (PA and FL imaging), but also differs in site of functionalization of the dye which is shown to be critical to its binding capacity (see Table 1*-[^{18}F] Maltotriose). It would not be obvious that the site of functionalization and the size of the functional group attached would be as critical as we found it to be.

- As for the animal models, we believe that it is important to assess new imaging agents in already well-established infection models to allow proper evaluation as well as head-to-head

comparison to other mentioned imaging agents. Hence, further exploring other infection murine models will be redundant since these *in vivo* models will still differ from humans.

- We mistakenly missed adding the Takemiya paper [2] which is from the same group we referenced previously in the discussion and combines the use of their imaging agent developed previously, for PET [5] and Optical imaging[1], into one paper and is now added to the revised references.
- We have also moved the paragraph from the discussion to Introduction to mention maltodextrin based agents earlier in the text.

"Murthy and coworkers developed a fluorescent [30] and an ^{18}F-labeled [31] derivative of maltohexose at the anomeric carbon and showed its effectiveness in fluorescence and PET imaging of bacterial infections in rats respectively [32]. Recently, Pang and coworkers developed theranostic nanoparticles loaded with purpurin 18 and targeted to bacteria by surface functionalization to maltohexose [33]. These particles showed great potential in treating bacteria using sonodynamic therapy and assessed the specificity of their particles to infection site using FLI imaging and showed an example of PAI using their particles."

In summary, our current research is the first example of the use of a maltotriose-based imaging agent for both fluorescence and photoacoustic imaging of bacterial infections. We also show solid evidence highlighting the superiority of this maltotriose-based imaging agent in direct comparison to the maltohexose agent developed by other groups and showcase better suitability of our scaffold for clinical translation. In agreement with the other reviewers of this paper, we believe the application of this maltotriose-based imaging agent is highly novel and the data presented is important to the field.

Reviewer #2

Summary of response:

We thank the reviewer for his/her thoughtful comments and have added new data and discussion to the manuscript to address his/her concerns.

Reviewer #2 (Remarks to the Author):

The authors reported a new fluorescent derivative of maltotriose as a bacterial infection imaging agent. They demonstrated its performance using both fluorescence and photoacoustic imaging. Some technical issues regarding photoacoustic imaging should be addressed.

1. It would be helpful if the authors could provide the absorption spectrum of the developed imaging agent. What is the estimated molar concentration of the imaging agent in the infected tissues *in vivo*?

- Absorption and Emission spectra show no change in the fluorescent properties of the Cy7 dye upon conjugation to either maltotriose or maltohexose (Figure S 5**c**, λ_{max}= 750 and 780nm for absorption and emission respectively).

- It is very challenging to estimate the concentration of the probe in the infected muscle *in vivo* since the signal is affected by many factors such as tissue interference, clearance, decomposition overtime and accumulation. But looking at the average fluorescence in the infected muscle from one of the *in vivo* FLI studies (Average radiance efficiency = $1.86 \times 10^8 \pm 3.40 \times 10^7$) and using the calibration curve (Figure S7**b**-FLI; Slope: $y= 1e^8x + 7e^7$ where y: FLI signal and x: number of nmol) the estimate amount of probe in the infection is around 1.16 ± 0.34 nmols without taking into account signals artifacts from the skin and body.

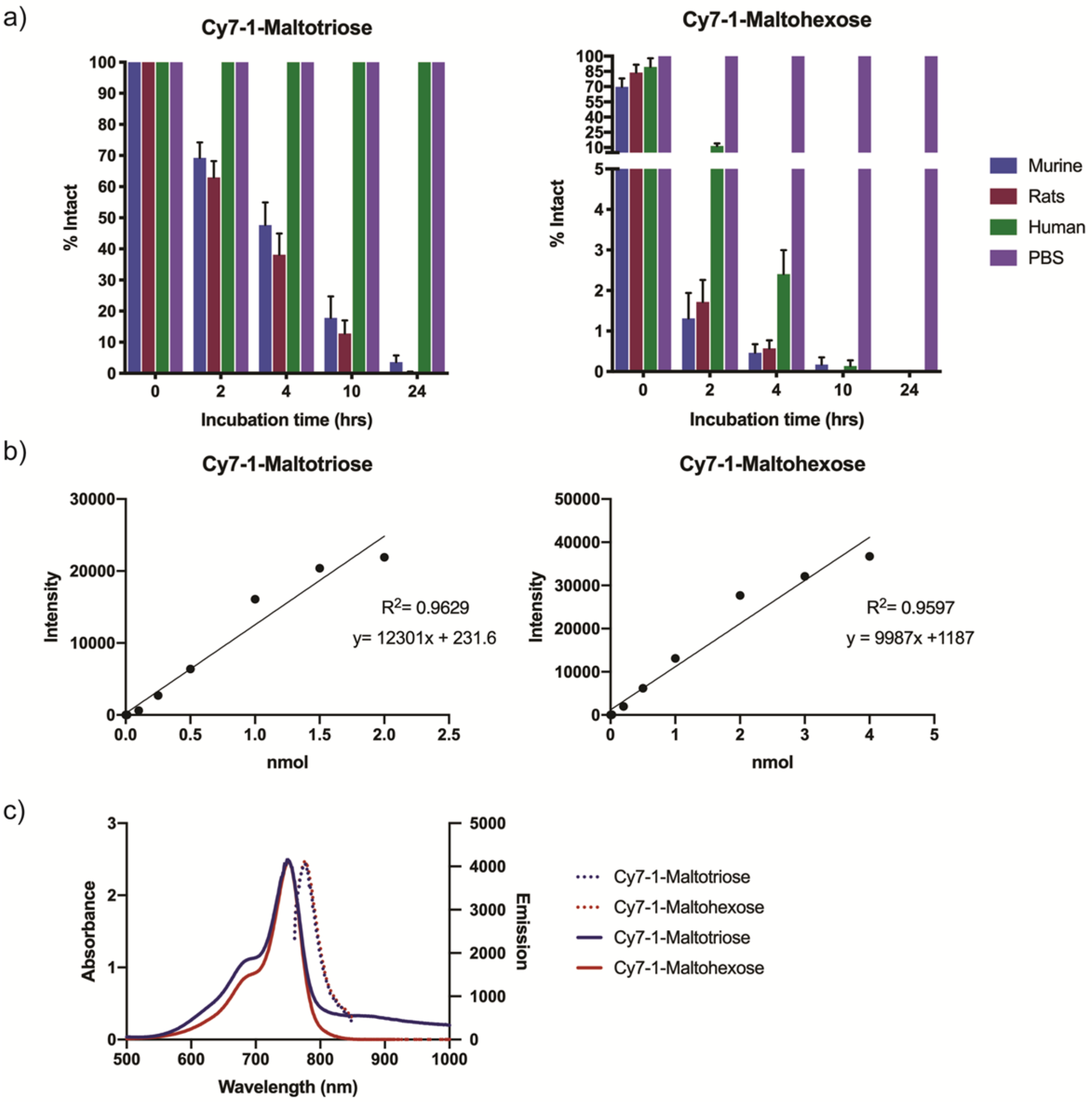

Figure S 5. In vitro characterization of Cy7-1-maltotriose and Cy7-1-maltohexose. **a)** Murine (n=6), rats (n=4), and human (n=6) plasma and PBS (n=4) stability assessment after incubation at 37ºC for 0, 2, 4, 10 and 24 hrs. At the different time points samples were analyzed on analytical HPLC. Data presented as area under the peak representing the compound of interest over area of all peaks observed in the HPLC trace when monitoring at 750nm. **b)** HPLC detection limit of both Cy7-1-maltotriose (left) and Cy7-1-maltohexose (right). **c)** Absorption (solid line) and emission (dotted line) spectra of Cy7-1-maltotriose (navy) and Cy7-1-maltohexose (maroon).

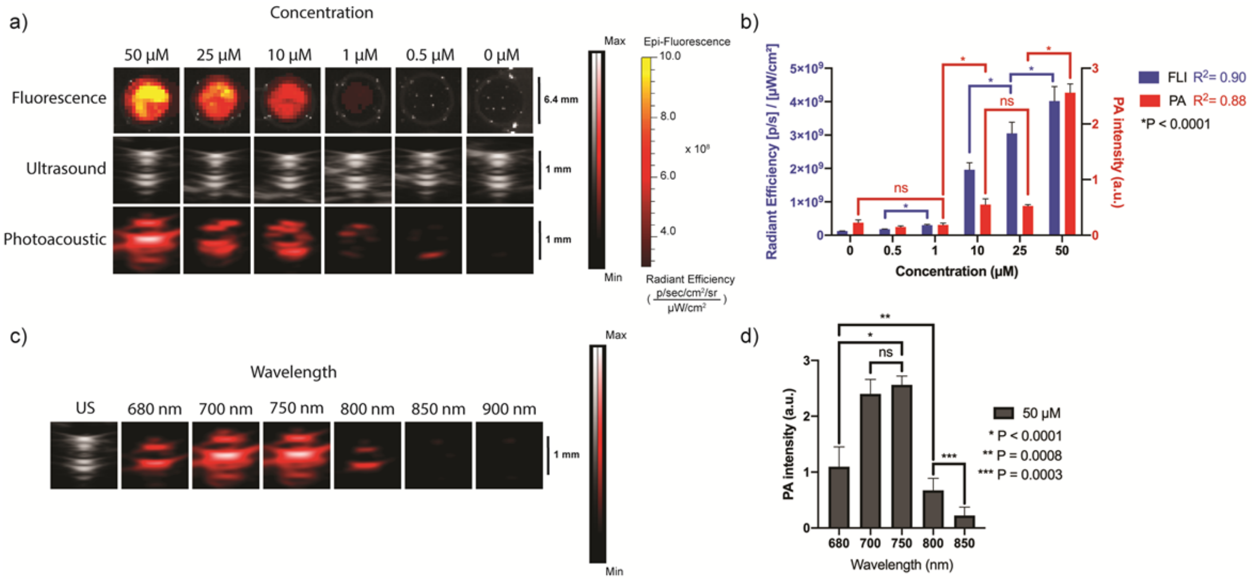

Figure S 7. In vitro characterization of Cy7-1-Maltotriose. **a)** Fluorescence, ultrasound and photoacoustic images of a phantom containing different concentrations of Cy7-1-maltotriose. Evident reduction in fluorescence and PA signal was observed with decrease in concentration. **b)** Bar plot showing linear correlation between Fluorescence (blue) or Photoacoustic (red) signal and the concentration of the agent. **c)** Photoacoustic imaging of a tube phantom containing 50µM solution of Cy7-1-maltotriose at different excitation wavelengths. **d)** Bar plot representation of the quantified PA signal when exciting at different wavelengths. No significant difference between excitation at 700nm and 750nm was observed and that range resulted in the highest PA signal intensity (n= 4). Statistical analysis was performed using one-way ANOVA.

2. Although hemoglobin has a smaller molar absorption coefficient in the near-infrared light region than visible light region, its high concentration in living tissues can still lead to strong photoacoustic signals. Have the authors compared the photoacoustic signals from the imaging agents (at a similar concentration for in vivo cases) with whole blood at the imaging wavelength of 700 nm?

- Looking at the absorption spectra in Figure S5**c** we find overlap with some intrinsic signals such as deoxygenated whole-blood[7]. We also found that our probe produces significantly higher PA intensity at 680, 700, 750 and 800 nm than background and negligible signal beyond 850nm excitation (Figure S7**d**). In our study, we are able to significantly differentiate between the PA signal in infected site compared to control images such as uninfected site, or imaging before injection of the probe (Figure 3**d** and 4**d**) which should include any intrinsic signals beyond that produced from the imaging agent.

- We believe that our imaging strategy was sufficient to evaluate our probe and acknowledge in the discussion (see below) the limitations as well as further developments necessary in the imaging agent, system and imaging parameters for proper distinction and quantification of the molecular signal.

"Furthermore, possessing more red-shifted as well as distinctive absorbance characteristics from intrinsic signals (such as hemoglobin and melanin) will further improve imaging depth and signal quantification respectively."

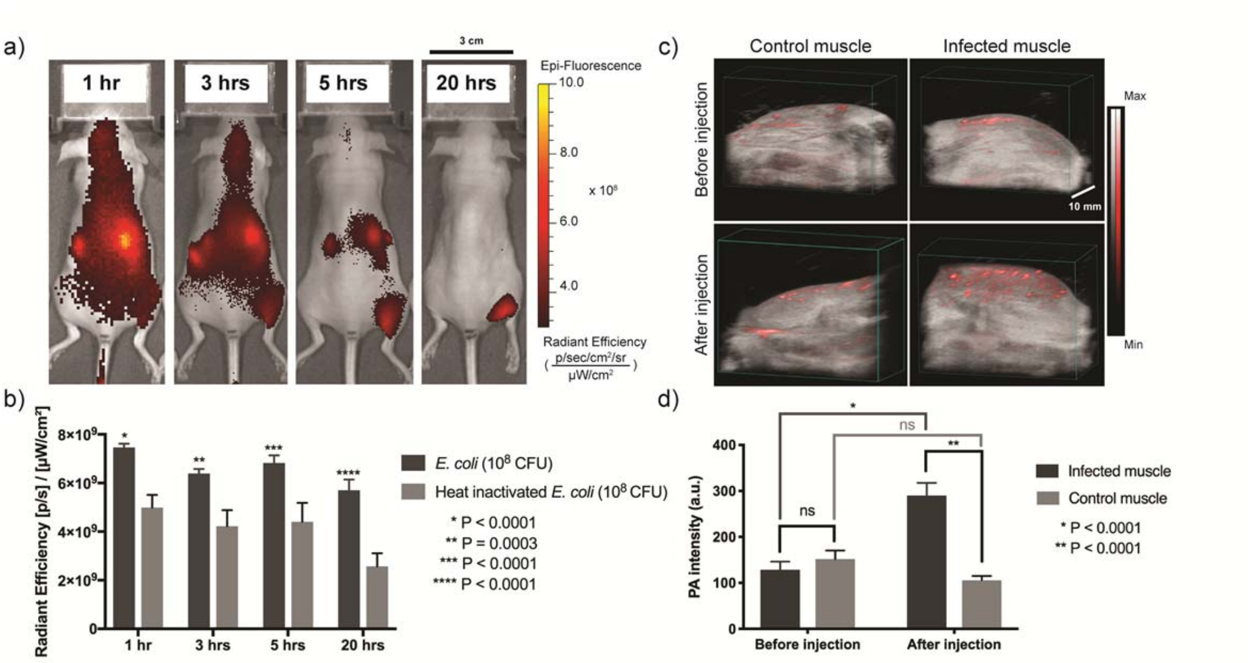

Figure 3. In vivo validation of Cy7-1-maltotriose in an E. coli-induced myositis murine model. **a)** Fluorescence imaging shows accumulation of Cy7-1-maltotriose in *E. coli*-infected thigh muscle as early as 1 hr post systemic injection (right thigh muscle). No evident accumulation of the agent in thigh muscle injected with 10^8 CFUs of heat inactivated *E. coli* (left thigh muscle). **b)** Bar-plot representation of quantified fluorescence signal in right and left thigh muscle overtime. As early as 1 hr post injection, a significantly higher fluorescence signal was found in muscle infected with *E. coli* (right thigh) compared to muscle infected with heat-inactivated *E. coli* (left thigh) (n=4). **c)** 3D rendered photoacoustic image overlaid on ultrasound image of a mouse's left (control) and right (infected) thigh muscle before and 20 hrs post injection of Cy7-1-maltotriose. Qualitatively, the image of the infected thigh muscle (bottom right) shows higher photoacoustic signal relative to image of the same muscle acquired before probe injection (top right) and image of the control thigh muscle (bottom left). **d)** Bar plot representation of the quantified photoacoustic signal intensity in the photoacoustic images acquired before and 20 hrs after injection of Cy7-1-maltotriose. The quantified signal of the infected thigh muscle was significantly higher post injection of the probe compared to that before injection (n=4 and 5 respectively, *P = 0.0001). In addition, the post injection signal in the infected thigh muscle was

significantly higher than that of the control muscle (n=4, P = 0.0001). Statistical analysis was performed using one-way ANOVA. ns: no statistically significant difference (P > 0.05).

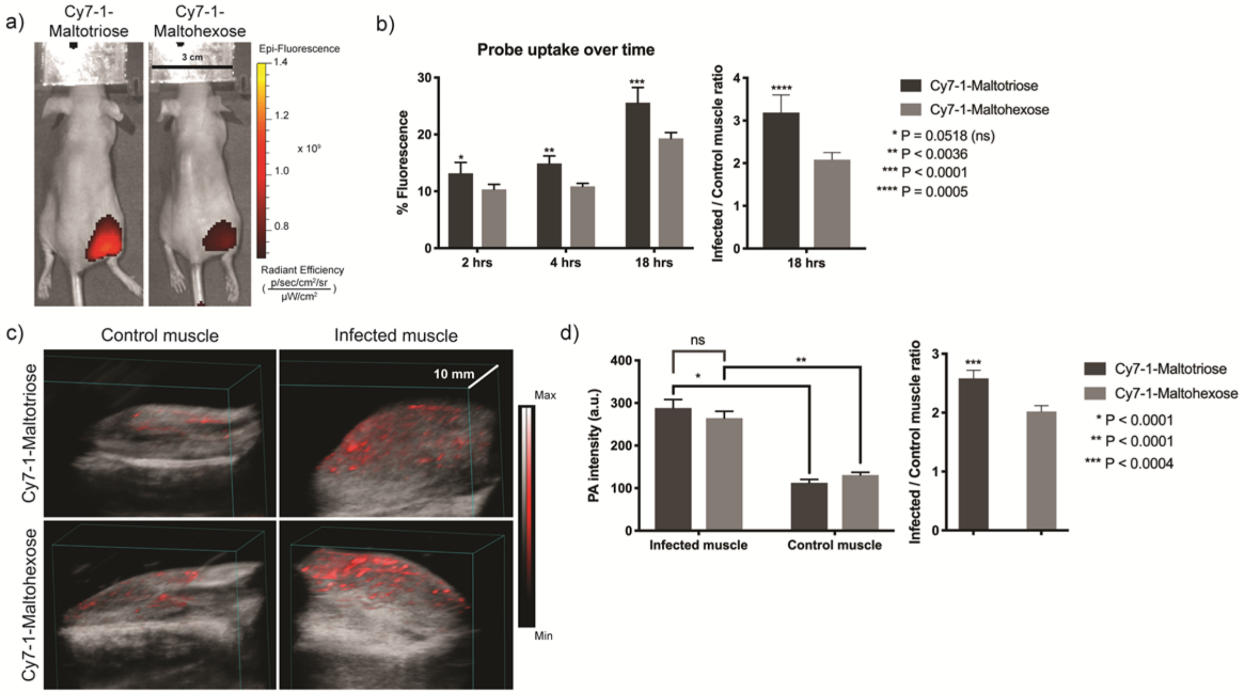

Figure 4. In vivo comparison between Cy7-1-Maltotriose and Cy7-1-Maltohexose in an E. coli-induced myositis murine model. **a)** *In vivo* fluorescence images at 18 hrs post injection of either Cy7-1-maltotriose (left) or Cy7-1-maltohexose (right) showing accumulation of both probes in the right thigh muscle (*E. coli*). Qualitatively higher fluorescence signal in the infected muscle (right thigh) when injecting Cy7-1-maltotriose (left) compared to Cy7-1-maltohexose (right) is observed. **b)** Bar plot representation of % Fluorescence in the infected muscle compared to whole body (left) and ratio of fluorescence in the infected thigh versus control thigh (right). Starting at 4 and 18 hrs post injection, significantly higher fluorescence signal in the infected muscle (right thigh) when injecting Cy7-1-maltotriose was quantified compared to injecting Cy7-1-maltohexose (n=6, P < 0.0036 and n=4, P < 0.0001 respectively). **c)** 3D rendered photoacoustic image overlaid on ultrasound image of a mouse 21 hrs post injection of Cy7-1-maltotriose (top) and Cy7-1-maltohexose (bottom). Images show Infected (right thigh) and control (left thigh) muscle. Evident PA signal is observed in the infected thigh muscle when injecting either compounds while minimal signal is observed in the control thigh muscle. **d)** Bar plot representation of the quantified photoacoustic signal intensity in the photoacoustic images acquired 21 hrs after injection of Cy7-1-maltotriose and Cy7-1-maltohexose. The quantified signal shows significantly higher PA signal in the infected muscle compared to control muscle when injecting either compounds (left). No significant difference in PA signal was observed between the two compounds (P = 0.139). Significantly higher infected over control PA signal ratio was observed when injecting Cy7-1-maltotriose (n= 6) compared to Cy7-1-maltohexose (n= 3) (P< 0.0004) (right). Statistical analysis was performed using one-way ANOVA. ns: no statistically significant difference (P > 0.05).

3. It seems that the optoacoustic signals of blood (or other endogenous chromophores) are not negligible (Fig. 3c, before injection). The detected optoacoustic signals (post-injection) are the integrated signals from both imaging agents and the endogenous chromophores. It is better to add at least one more imaging wavelength for this experiment; then the authors can quantitatively analyze the contributions from the imaging agents and blood.

Please refer to comment 2. We also added in the discussion:

"Furthermore, several hurdles related to accurate signal quantification (such as differentiating intrinsic signal from imaging agent, reduced accuracy with increased depth) need to be addressed to conduct molecular PAI of disease [16]. Such limitations can be somewhat mitigated by proper choice of the photoacoustic dye (as mentioned above), choosing the best excitation wavelengths that can assist in differentiating the signal (eg. background subtraction) and the use of models for fluence correction[16]."

4. It will be more readable if the authors can add scale bars to both the fluorescence and photoacoustic images.

Per request scale bars were added to all images

Reviewer #3

Summary of response:

We thank the reviewer for his/her positive comments regarding the writing and construction of the manuscript and appreciate their acknowledgement on the benefits of this application in bacterial infection imaging and diagnosis. In response to the thoughtful comments by the reviewer, we have added new data and discussion to the manuscript to address his/her concerns.

Reviewer #3 (Remarks to the Author):

Drug-resistance to antibiotics is a rapidly growing problem, and early detection of this problem is fundamentally important. With this in mind, this paper is highly relevant, and the topic could be of interest to many readers. The authors report a fluorescent derivative of maltodextrin, Cy7-1-maltotriose for fluorescent and optoacoustic imaging and illustrate its application in the imaging of bacterial infections. The authors present good data, testing models included bacterial cells, tissue culture and mouse models. The aim is for a more local evaluation of infected sites. 18F and fluorescent analogs of maltose and maltotriose were previously shown to specifically image bacterial infections. Optoacoustic imaging presents an attractive feature over optical imaging modalities. The manuscript is well written and constructed thoughtfully. The design of dual modality sensor based on sugars for specifically imaging bacteria should usher new developments in this arena. However, the overall results are in part underdeveloped with respect to the optoacoustic bioimaging. Furthermore, the review of the literature is somewhat incomplete, since there are already a few optoacoustic imaging technologies for bacterial infections in vivo that exist, none of which were discussed or cited.

Specific comments include:

1. “To the best of our knowledge, this is the first demonstration of molecular PAI of bacterial infections in living mice. “ –Please see the examples of previous work listed below. The following works should have been acknowledged. Strong arguments for the advantages of Cy-7-1-maltotriose over the already published agents should be discussed.

- We have added all the relevant references to the introduction (see below)

"A number of FLI and / or PAI probes targeted to bacteria have been developed by using antibiotics (vancomycin [19,20] or teicoplanin [21],specific to gram positive bacteria), Concanavalin (targeting bacterial cell-surface mannose) [22], antibodies (targeting the immunodominant staphylococcal antigen A, specific to *S. aureus*) [23], boronic-acid (targeting bacterial cell-surface glycoproteins, specific to gram positive bacteria) [24], enzyme-activated nanoparticles (targeting gelatinase-expressing gram positive bacteria) [20] or through electrostatic and hydrophobic interactions (specific to gram positive bacteria)[25]. Preclinical evaluation of these probes showed promising results in FLI[19,22-24] or PAI [20,21] of some bacterial infections. Unfortunately, targeting bacterial cell wall potentially limits the amount of signaling agent taken up leading to lower sensitivity. In addition, strain-specific probes will have minimal clinical impact in imaging surgery and injury related bacterial infections since they usually occur from the presence of a variety of pathogenic bacteria. A few other examples rely on genetically encoding bacteria with reporters, such as photo-switchable chromoproteins [26,27] and violacein [28], have been reported. While these strategies allow noninvasive imaging of bacteria *in vivo* using photoacoustic imaging, the application of such platform would be limited to visualizing biochemistry, pathophysiological processes and gene expression profiles in living subjects as well as imaging tumor homing bacteria [29] and cannot be used for diagnosing bacterial infections."

- Below is a summary of each publication and advantages of our strategy

- Violacein as a genetically-controlled, enzymatically amplified and photobleaching-resistant chromophore for optoacoustic bacterial imaging", Scientific Reports 5,11048 (2015). [17]

This reference reports genetically encoding bacteria to express a chromophore detectable using photoacoustic imaging and further supports the utility of PAI in imaging bacteria *in vivo*. As added to the text, this strategy allows noninvasive monitoring of biochemical and physiological processes and cannot be used for bacterial infection diagnosis as it first requires genetically re-engineering the bacteria of interest.

- "In vivo photoacoustic difference-spectra imaging of bacteria using photoswitchable chromoproteins", J. of Biomedical Optics, 23(10), 106006 (2018).[16]

This report relies on genetically encoding bacteria to express photoswitchable chromoproteins. As mentioned above, such strategies are incredibly useful to monitor bacteria in vivo (tumor homing bacteria in this case) and highlight the utility of PAI in imaging. This tool

cannot be used for molecular imaging and diagnosis of bacterial infections as it first requires genetically re-engineering the bacteria of interest.

- Pathological□Condition□Driven Construction of Supramolecular Nanoassemblies for Bacterial Infection Detection, Advanced Materials, 2015, 28, issue 2, 254-262. [9]

This reference indeed reports photoacoustic imaging of bacterial infections. Unfortunately, this type of imaging agent is only specific to gram positive and gelatinase expressing bacteria. This limits its application in bacterial infection diagnosis since knowing the type of pathogen prior to imaging will be required. In addition, we believe that detection and diagnosis of bacterial infections by relying on antibiotics will not be sustainable (decrease in amount of effective antibiotics), nor generalizable (infections usually occur from multiple pathogens) and can further contribute to increasing antibiotic-resistant pathogens (due to exposing pathogens to low doses of the drug during imaging).

- "A purpurin-peptide derivative for selective killing of Gram-positive bacteria via insertion into cell membrane", J. Mater. Chem. B, 2016,4, 4855-4861. [14]

This theranostic agent relies on electrostatic and hydrophobic interactions to target bacteria. Unfortunately, this agent is only targeted to gram +ve bacteria (see discussion above) and no *in vivo* validation of this agent was reported.

- "A Gold/Silver Hybrid Nanoparticle for Treatment and Photoacoustic Imaging of Bacterial Infection", ACS Nano, 2018, 12 (6), pp 5615–5625. [19]

This paper reports gold/silver-based nanoparticles that can be used to kill bacteria upon etching. While the title of the paper states PAI of bacterial infections, this report only images the gold nanorods using PA and there is no report of actual imaging of bacteria. In addition, these particles and the etching material were subcutaneously injected into the wound and no specific targeting nor systemic injection were demonstrated. This report only highlights effectiveness of the particles in killing bacteria upon etching and highlights noninvasive and real-time dosimetry of Ag+ ions using PAI. This publication was not added to the references

- https://www.visualsonics.com/resource/featured-images/imaging-bacterial-infection [10]

While this study had a small number of replicates (n=2), it indeed shows a great example of PAI of bacterial infections in a very challenging prosthetic joint infection murine model. Unfortunately, this study also relies on antibiotics as a targeting moiety limiting it to only gram +ve bacteria (see prior discussion on the limitations of using antibiotics compared to metabolic

based agents). This study further highlights the importance and advantages of PAI in bacterial infection diagnosis.

- Bacteria-Responsive Nanoliposomes as Smart Sonotheranostics for Multidrug Resistant Bacterial Infections.[6]

This is a recent report of theranostic nanoparticles loaded with purpurin 18 and targeted to bacteria by surface functionalization to maltohexose. These particles showed great potential in treating bacteria using sonodynamic therapy and selective FLI to a variety of controls and showed one example of PAI of the infection site. In addition to the limitations of maltohexose compared to maltotriose discussed in our report (mainly stability and speed of uptake), this publication only shows thorough evaluation using FLI and an example of PAI (with no controls such as pre/post injection or infected vs control or pre/post treatment). Furthermore, the translation of nanoparticle based agents could potentially face increased hurdles compared to small molecule based probes.

2. The authors set out to synthesize a probe (Line 232) for optoacoustic imaging at greater depths. However, in Fig 3c. the scale bar is missing. At which depths were the authors able to image and still see differences in the signal? It is a challenge to see the signal differences in Fig. 3c between the control muscle and the infected muscle. Would a 3D volume reconstruction best present the data?

- Added a scale bar to all images (Figures 3, 4, 5 and 6).
- Imaging depth in this case was up to 20mm. In this study we did not evaluate how deep can we image the infection since this will significantly change in different mediums (murine vs. human), changing the dye, as well as changing the PAI system.
- Indeed, the 3D reconstruction seemed to show better distinction between infected and controls (Figures 3, 4 and 6). Quantitation of those images did not contradict quantification data obtained from the 2D images. Looking at the ratios of infected/control signals, we find similar ratios in PA to that in FLI (~2.5-3 fold for maltotriose and 2 fold for maltohexose).

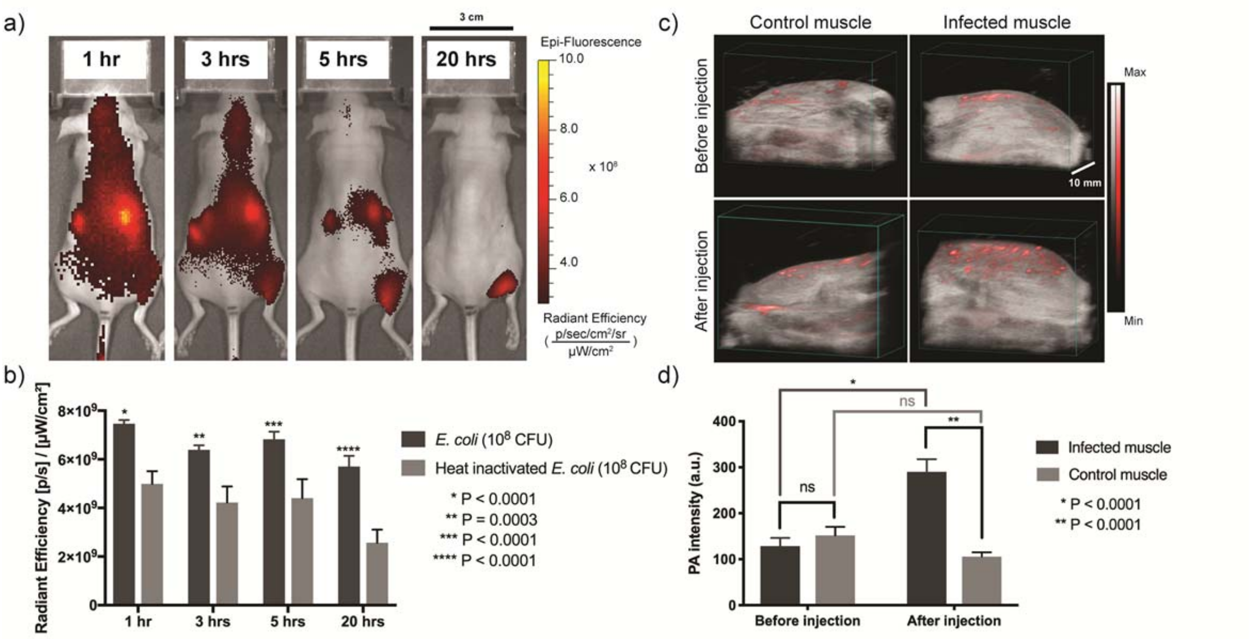

Figure 3. In vivo validation of Cy7-1-maltotriose in an E. coli-induced myositis murine model. **a)** Fluorescence imaging shows accumulation of Cy7-1-maltotriose in *E. coli*-infected thigh muscle as early as 1 hr post systemic injection (right thigh muscle). No evident accumulation of the agent in thigh muscle injected with 10^8 CFUs of heat inactivated *E. coli* (left thigh muscle). **b)** Bar-plot representation of quantified fluorescence signal in right and left thigh muscle overtime. As early as 1 hr post injection, a significantly higher fluorescence signal was found in muscle infected with *E. coli* (right thigh) compared to muscle infected with heat-inactivated *E. coli* (left thigh) (n=4). **c)** 3D rendered photoacoustic image overlaid on ultrasound image of a mouse's left (control) and right (infected) thigh muscle before and 20 hrs post injection of Cy7-1-maltotriose. Qualitatively, the image of the infected thigh muscle (bottom right) shows higher photoacoustic signal relative to image of the same muscle acquired before probe injection (top right) and image of the control thigh muscle (bottom left). **d)** Bar plot representation of the quantified photoacoustic signal intensity in the photoacoustic images acquired before and 20 hrs after injection of Cy7-1-maltotriose. The quantified signal of the infected thigh muscle was significantly higher post injection of the probe compared to that before injection (n=4 and 5 respectively, *P = 0.0001). In addition, the post injection signal in the infected thigh muscle was significantly higher than that of the control muscle (n=4, P = 0.0001). Statistical analysis was performed using one-way ANOVA. ns: no statistically significant difference (P > 0.05).

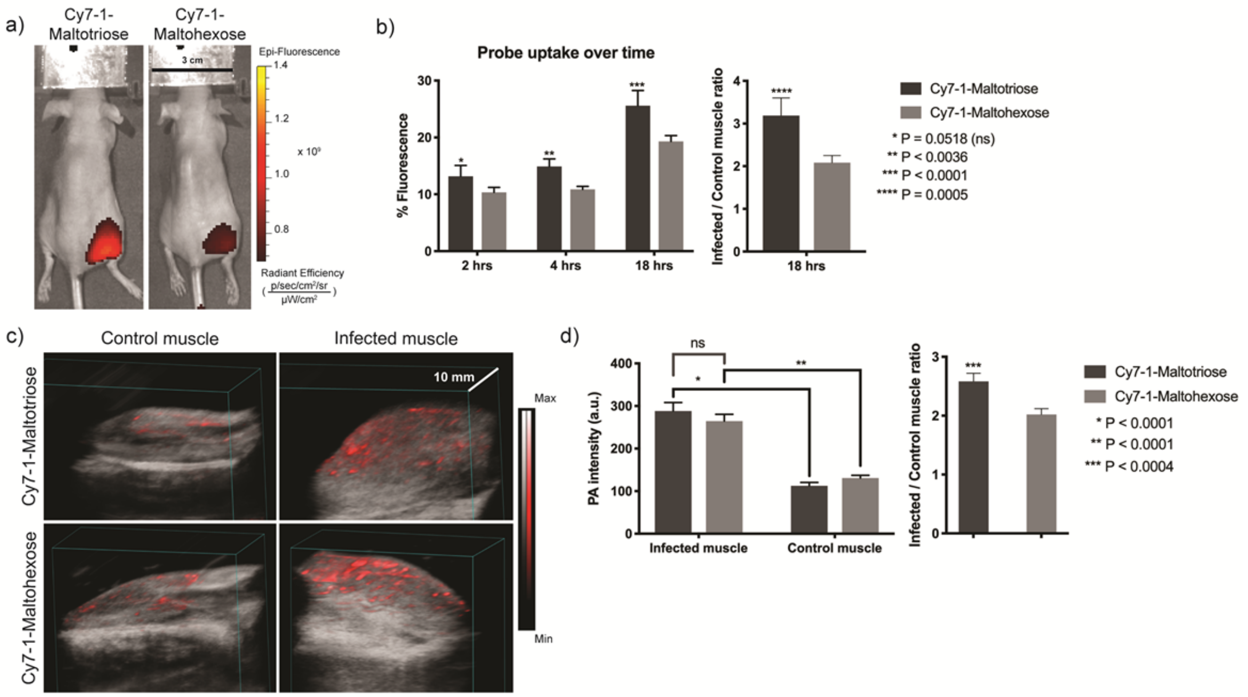

Figure 4. In vivo comparison between Cy7-1-maltotriose and Cy7-1-maltohexose in an E. coli-induced myositis murine model. **a)** *In vivo* fluorescence images at 18 hrs post injection of either Cy7-1-maltotriose (left) or Cy7-1-maltohexose (right) showing accumulation of both probes in the right thigh muscle (*E. coli*). Qualitatively higher fluorescence signal in the infected muscle (right thigh) when injecting Cy7-1-maltotriose (left) compared to Cy7-1-maltohexose (right) is observed. **b)** Bar plot representation of % Fluorescence in the infected muscle compared to whole body (left) and ratio of fluorescence in the infected thigh versus control thigh (right). Starting at 4 and 18 hrs post injection, significantly higher fluorescence signal in the infected muscle (right thigh) when injecting Cy7-1-maltotriose was quantified compared to injecting Cy7-1-maltohexose (n=6, $P < 0.0036$ and n=4, $P < 0.0001$ respectively). **c)** 3D rendered photoacoustic image overlaid on ultrasound image of a mouse 21 hrs post injection of Cy7-1-maltotriose (top) and Cy7-1-maltohexose (bottom). Images show Infected (right thigh) and control (left thigh) muscle. Evident PA signal is observed in the infected thigh muscle when injecting either compounds while minimal signal is observed in the control thigh muscle. **d)** Bar plot representation of the quantified photoacoustic signal intensity in the photoacoustic images acquired 21 hrs after injection of Cy7-1-maltotriose and Cy7-1-maltohexose. The quantified signal shows significantly higher PA signal in the infected muscle compared to control muscle when injecting either compounds (left). No significant difference in PA signal was observed between the two compounds ($P = 0.139$). Significantly higher infected over control PA signal ratio was observed when injecting Cy7-1-maltotriose (n= 6) compared to Cy7-1-maltohexose (n= 3) ($P< 0.0004$) (right). Statistical analysis was performed using one-way ANOVA. ns: no statistically significant difference ($P > 0.05$).

3. Optoacoustic imaging shown were carried out 20 h post injection of Cy7-1-maltotriose. Stability studies in serum and 2% DMSO/saline is highly recommended.

- This was a great suggestion and indeed showcased another important advantage of the maltotriose derivative compared to maltohexose. More specifically, it seems that the maltohexose derivative rapidly breaks down into smaller sugar entities within minutes of incubation in rat and murine serum (Figure S5**a**). This suggests that any data previously observed using the maltohexose derivative relies heavily on the internalization of the broken-down moieties and minimally on the actual maltohexose scaffold. Furthermore, in human plasma it seems that there is no sign of maltotriose decomposition overtime (also observed by Axer and coworkers), while ~10% of the maltohexose derivative stayed intact after 2 hrs, further highlighting the clinical translation potential of the maltotriose scaffold.

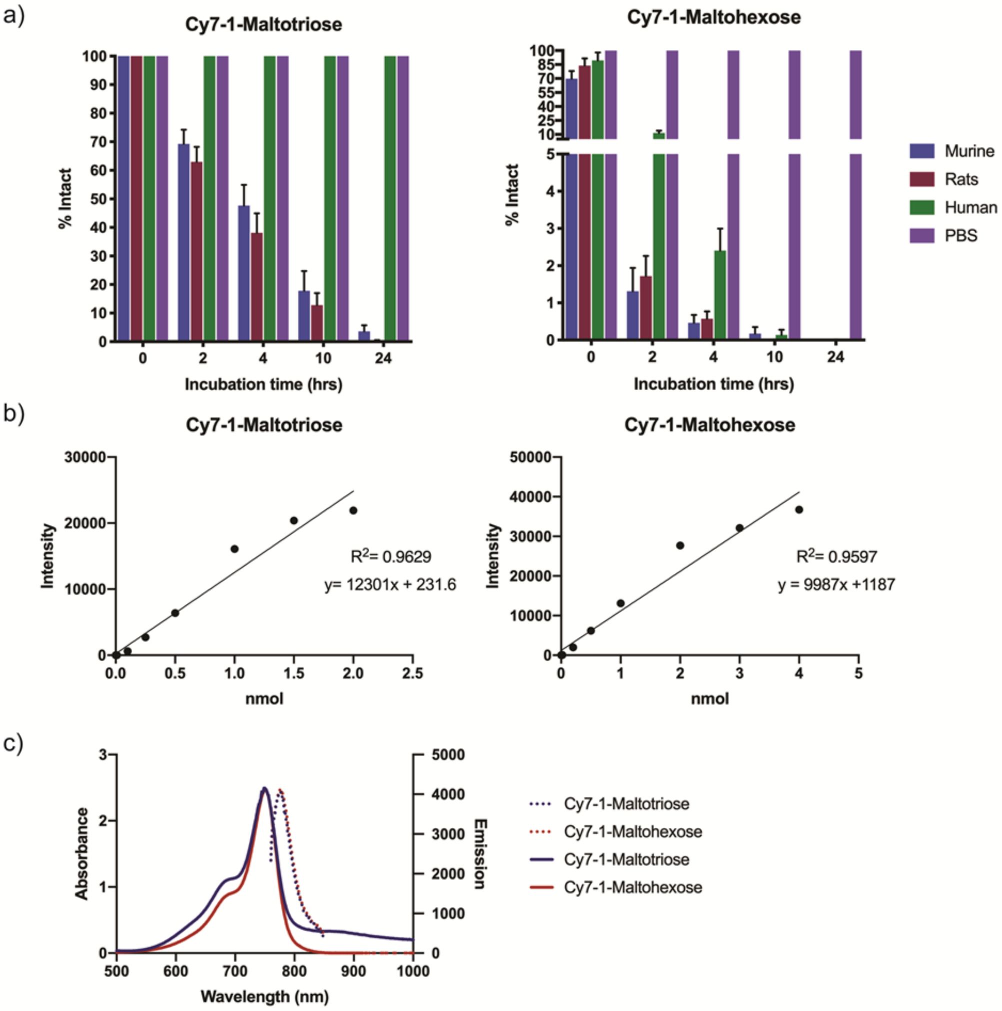

Figure S 5. In vitro characterization of Cy7-1-maltotriose and Cy7-1-maltohexose. **a)** Murine (n=6), rats (n=4), and human (n=6) plasma and PBS (n=4) stability assessment after incubation at 37ºC for 0, 2, 4, 10 and 24 hrs. At the different time points samples were analyzed on analytical HPLC. Data presented as area under the peak representing the compound of interest over area of all peaks observed in the HPLC trace when monitoring at 750nm. **b)** HPLC

detection limit of both Cy7-1-maltotriose (left) and Cy7-1-maltohexose (right). **c)** Absorption (solid line) and emission (dotted line) spectra of Cy7-1-maltotriose (navy) and Cy7-1-maltohexose (maroon).

4. "Fluorescence and Photoacoustic imaging…" is the title and features arguments for optoacoustic imaging throughout the entire manuscript (line 233, 344 etc.). The paper requires more optoacoustic validation of the probe. So far, in vivo data are only shown once in Fig. 3c. Fluorescence data shown in Fig. 4. is great, but parallel acquisition should also be performed in the optoacoustic system.

- We have added PA imaging on the wound infection model and replaced the CD1 mice with SKH-1 hairless mice to overcome issues of fur growth and interference with PA imaging which occurs on the wound after antibiotic treatment. In addition, we divided the study into two groups (treated and untreated) to show presence of infection in the untreated group (Figure 6).

- FLI, BLI and PAI showcased the effectiveness of treatment and showed significant differences in signal between the treated and untreated group.

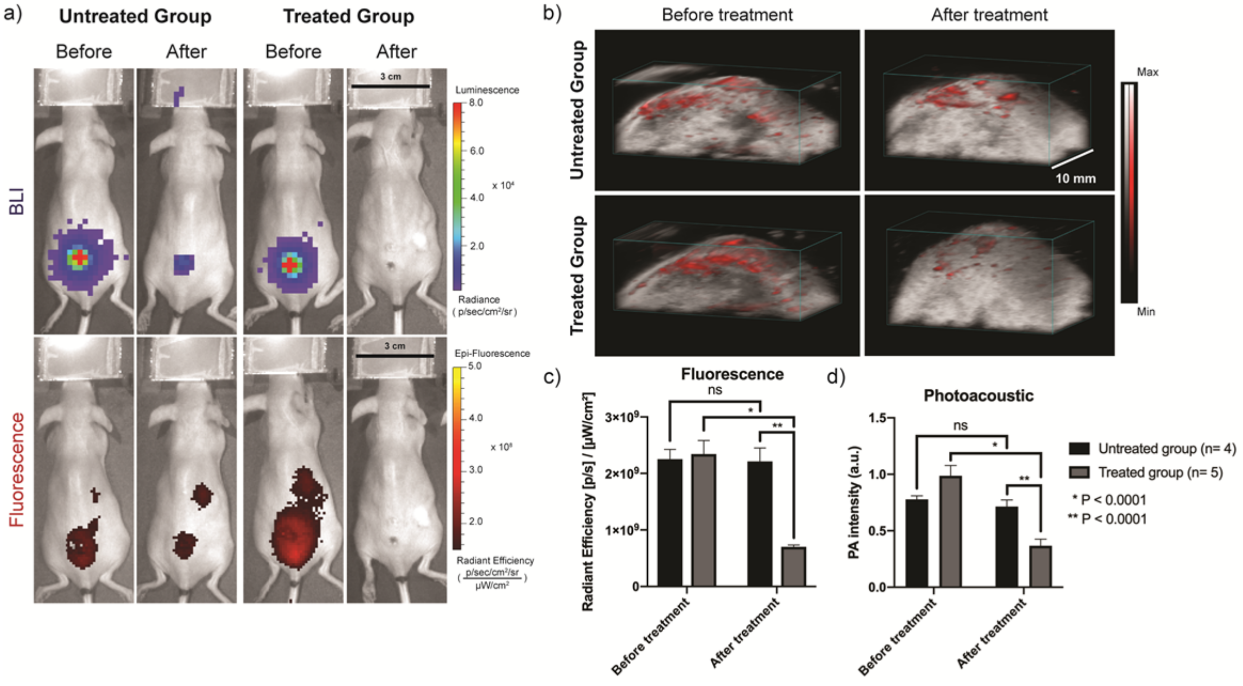

Figure 6. In vivo evaluation of Cy7-1-maltotriose in a S. aureus wound infection murine model. **a)** BLI and FLI images of mice with a wound infected with 10^6 CFUs of bioluminescent *S. aureus* 19 hrs post injection of Cy7-1-maltotriose without (Untreated Group) and with (Treated Group) treatment with vancomycin for 7 days. FLI (bottom-Before) shows accumulation of the probe in the *S. aureus* located by BLI (top-Before). In the untreated group (left panel) both BLI and FLI showcases presence of *S. aureus* infection in the wound after 7 days (Untreated Group – After). While in the treated group (right panel), complete disappearance

of *S. aureus* infection was observed in the FLI image and confirmed by BLI (Treated Group – After). **b)** 3D rendered PA image overlaid on US image of a mouse from Untreated (Top row) and Treated (Bottom row) groups before and after treatment. Images were acquired 20 hrs post injection of Cy7-1-maltotriose. Similar to observations in FLI, the Treated group showed lower PA signal post treatment (bottom right) than that before treatment (bottom left). **c)** Total *in vivo* fluorescence signal in wound infected with 10^6 CFUs of bioluminescent *S. aureus* 19 hrs after tail vein injection of Cy7-1-maltotriose. Significant reduction of FLI signal after treating the mice with vancomycin for 7 days in the treated group ($n = 5$, $P < 0.0001$) was observed, while no difference was observed in the untreated group before and after 7 days ($n=4$, $P > 0.99$). **d)** Bar plot representation of the quantified average PA signal intensity in the PA images acquired 20 hrs after injection of Cy7-1-maltotriose before and after antibiotic treatment. The quantified signal shows significant reduction in PA signal in the treated group after vancomycin treatment compared to before ($n=5$, $P < 0.0001$). No significant difference in PA signal in the untreated group was observed in the images acquired before and after treatment ($P= 0.54$). In addition, significantly higher PA signal was observed in the untreated group compared to the treated group after the treatment regimen ($P < 0.0001$). Statistical analysis was performed using one-way ANOVA. ns: no statistically significant difference ($P > 0.05$).

5. While the specificity of both agents was tested in vitro, this could also be carried out in vivo. Blocking studies are highly recommended in this case as a negative control. This would also further validate Fig. 3c. Does the fluorescence and optoacoustic data validate each other? It would be useful to repeat Fig 2b in an optoacoustic imaging setting.

- We have added another *in vivo* evaluation of the specificity of Cy7-1-maltotriose which also shows significantly higher uptake in *E. coli* infected muscle versus mutated *E. coli* (Figure S 8). We believe that with study and the previous reported controls we have validated the specificity of the imaging agent to maltodextrin transporter in bacteria.

- Looking at the ratios of infected/control signals, we find similar ratios in PA to that in FLI (~2.5-3 fold for maltotriose and 2 fold for maltohexose).

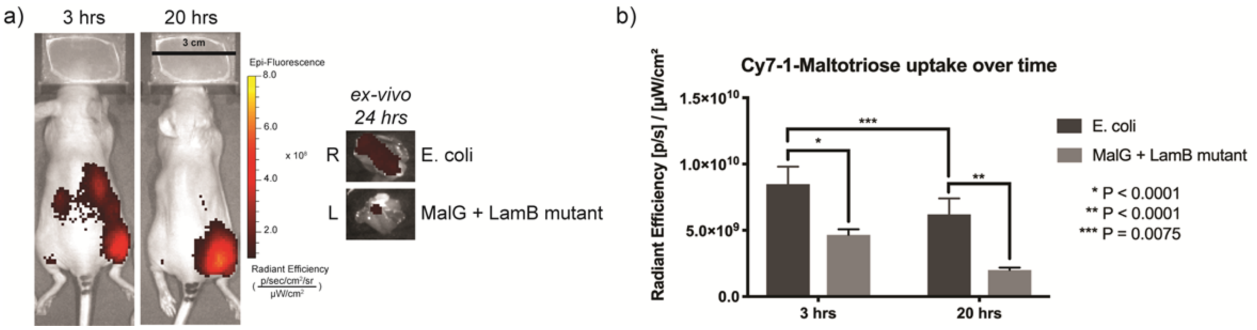

Figure S 8. In vivo validation of Cy7-1-maltotriose in an E. coli- and E.coli mutation-induced myositis murine model. **a)** Left: FLI shows accumulation of Cy7-1-maltotriose in E. coli-infected thigh muscle at 3 and 20 hrs post systemic injection (right thigh muscle). No evident accumulation of the agent in thigh muscle injected with 10^8 CFUs of E. coli MalG + LamB mutant (left thigh muscle). Right: Ex-vivo FLI of right and thigh muscle post excision. Image shows higher FLI signal in thigh muscle infected with E.coli compared to E.coli mutant. **b)** Bar-plot representation of quantified FLI signal in right and left thigh muscle at 3 and 20 hrs post probe injection. Significantly higher fluorescence signal was found in muscle infected with E. coli (right thigh) compared to muscle infected with E. coli mutant (left thigh) at both timepoints (n=5, P < 0.0001). Statistical analysis was performed using one-way ANOVA.

6. "Wounds infected with as low as 104 CFU were detectable by fluorescence imaging of Cy7-1-maltotriose." Which is great but what is the detectability in optoacoustic imaging system?

- While Cy7 provides PA properties, it is designed to be a fluorescent agent. Hence, we find the sensitivity of the agent in PA imaging is lower as seen in Figure S7**b**. In addition, we also observed in a pilot study that PA imaging of wounds infected with 10^6 CFU or more showed PA signal hinting that with this imaging system and Cy7 dye PAI can confidently detect wounds infected 10^6 CFU or more (Figure 2*).

- See below added discussion highlighting the limitation of this dye as well as potential solutions for future experiments

"While the Cy7 dye used in this imaging agent possess many great characteristics such as *in vivo* stability and optimum pharmacokinetics when bound to maltotriose, this dye is more suitable for fluorescence imaging than photoacoustic imaging. This could explain the higher sensitivity in detecting this probe using fluorescence imaging compared to photoacoustic imaging (Figure S7)"

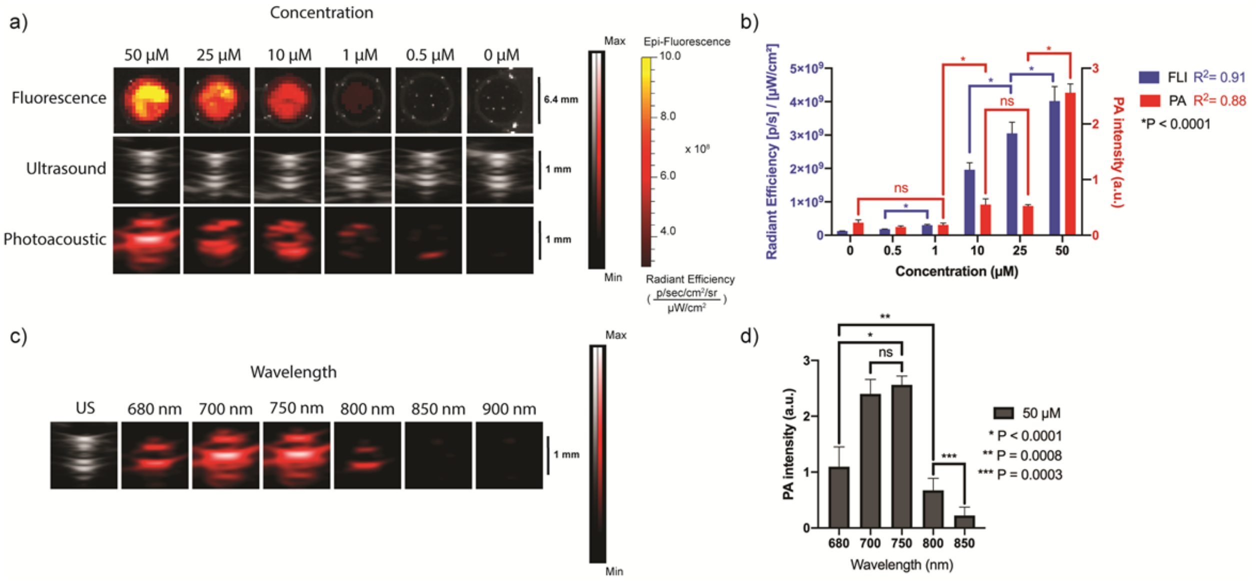

Figure S 7. In vitro characterization of Cy7-1-maltotriose. **a)** Fluorescence, ultrasound and photoacoustic images of a phantom containing different concentrations of Cy7-1-maltotriose. Evident reduction in Fluorescence and PA signal was observed with decrease in concentration. **b)** Bar plot showing linear correlation between Fluorescence (blue) or Photoacoustic (red) signal and the concentration of the agent. **c)** Photoacoustic imaging of a tube phantom containing 50µM solution of Cy7-1-maltotriose at different excitation wavelengths. **d)** Bar plot representation of the quantified PA signal when exciting at different wavelengths. No significant difference between excitation at 700nm and 750nm was observed and that range resulted in the highest PA signal intensity (n= 4). Statistical analysis was performed using one-way ANOVA.

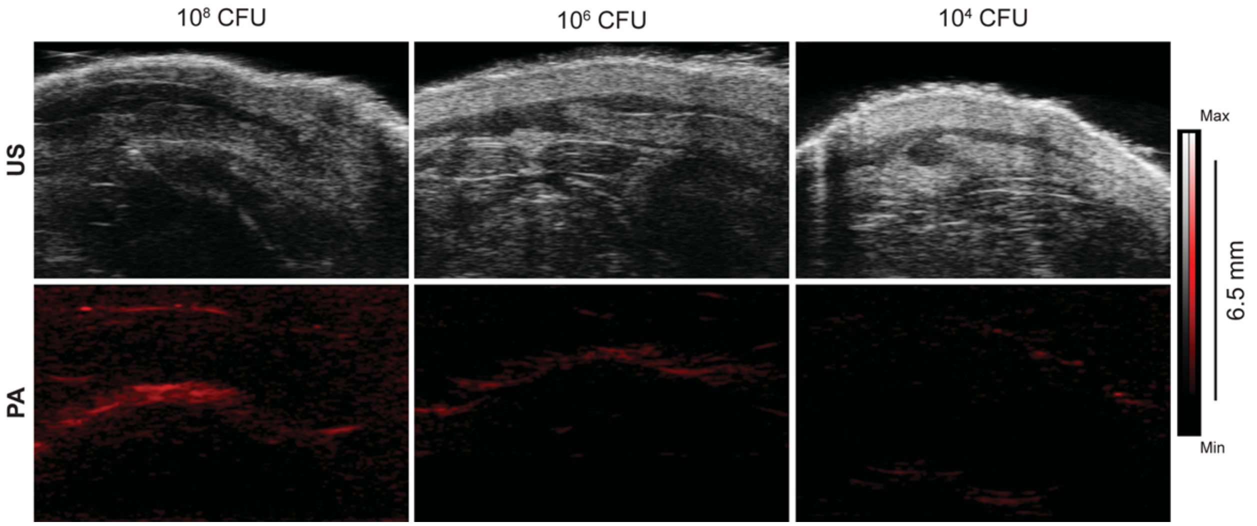

Figure 2*. Pilot 2D US and PA images in wound infected with different amount of CFU of S. aureus. Images qualitatively show more PA signal in wound infected with 10^8 then 10^6 CFU and almost no PA signal was detected in wound infected with 10^4 CFU. Images acquired 20 hrs post Cy7-1-maltotriose injection via the tail vein (5 nmol).

7. Line 37/38: “This will likely greatly enhance early detection and diagnosis of bacterial infections”- the authors should carefully reconsider or further discuss limitations of the sensor. Agents such as maltodextrins are metabolite based, its accumulation is very much dependent on the metabolic state of the bacteria. How close does the in vivo model of 108 bacteria injected mimic the real scenario for detectability?

- Added limitations of dye and PAI and possible means to overcome these hurdles

“While the Cy7 dye used in this imaging agent possess many great characteristics such as in vivo stability and optimum pharmacokinetics when bound to maltotriose, this dye is more suitable for fluorescence imaging than photoacoustic imaging. This could explain the higher sensitivity in detecting this probe using fluorescence imaging compared to photoacoustic imaging (Figure S7). In addition, this dye is not approved for clinical use. Hence, we are investigating attachment of a variety of clinically approved dyes as well as dyes more geared for PAI to further enhance its performance. More specifically, the dye needs to have great plasma stability, high photostability, low toxicity and ease of synthesis in a GMP facility. Furthermore, possessing more red-shifted as well as distinctive absorbance characteristics from intrinsic signals (such as hemoglobin and melanin) will further improve imaging depth and signal quantification respectively. The substitution of the dye may affect the distribution of the imaging agent and a thorough in vivo evaluation of the new probes will be necessary. We believe that we have identified the ideal scaffold for an infection imaging agent and with our established synthetic scheme we can quickly synthesize and clinically translate these new probes.

While PAI is growing to be a promising diagnostic tool, it still possesses several limitations which are being addressed. Namely, the limited field of view and imaging depth constricts its utility to localized imaging (no whole-body imaging). Similar to ultrasound imaging, this tool is highly dependent on operators experience and means to automate this technique will help overcome this issue and allow better and more accurate reproducibility of the images (e.g. Robotic handles and software tools to precisely delineate imaging plane for accurate reproducibility) [16]. Alternatively, 3D volume scanning should also assist in reducing the dependence of the operator’s experience. Furthermore, several hurdles related to accurate signal quantification (such as differentiating intrinsic signal from imaging agent, reduced accuracy with increased depth) need to be addressed to conduct molecular PAI of disease [16]. Such limitations can be somewhat mitigated by proper choice of the photoacoustic dye (as mentioned above),

choosing the best excitation wavelengths that can assist in differentiating the signal (eg. background subtraction) and the use of models for fluence correction[16]."

- In our studies, we found that inoculating 10^6-10^8 CFU of bacteria insures the development of the infection in the mouse model. In human wounds for example, reports state that a bacteria amount of around 10^4 CFU/gm of tissue or more is necessary to cause the infection [20].

8. Fig. 5c is missing a scale bar. It is unclear as to what amount/concentration/volume of Cy7-1-maltotriose was used for the experiment. 5 nmol? If that's the case, that's roughly 1 µM (assuming 200 µL i.v.) which does not seem very much in order to acquire decent optoacoustic imaging results. This would explain fig. 3c.

- Added scale bars to all images.
- The total amount injected in the studies was 5nmol (100µL injection of a 50µM solution). This amount seems to be very similar to ~70µM solution injected in another PAI study using ICG[10].
- For the revision antibiotic treatment study, 10nmol of compound was injected (200µL injection of a 50µM solution)
- We found that increasing the amount of injection beyond 10nmol only increased background signal and allowed for slightly longer imaging overtime (Figure 3*).

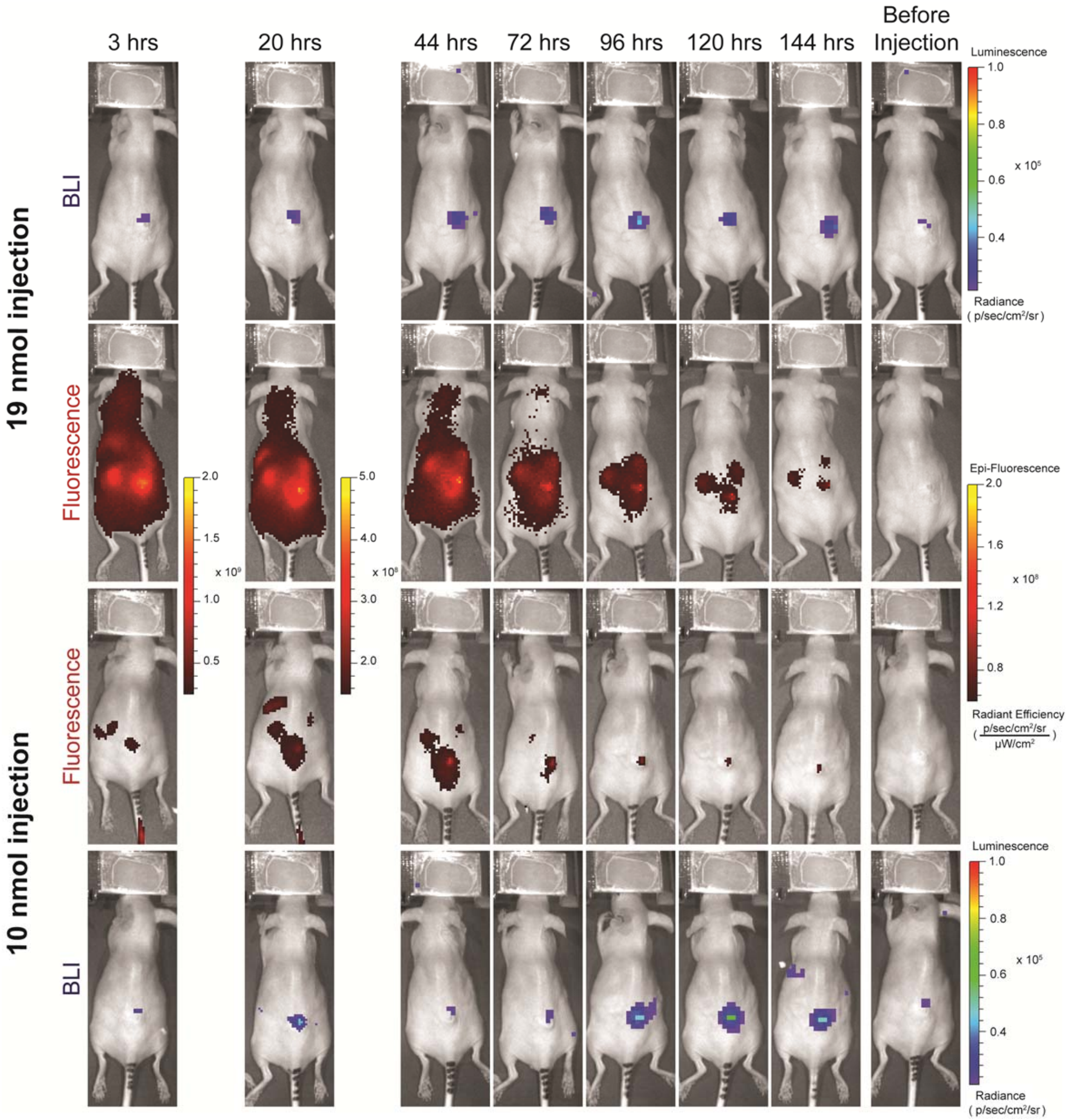

Figure 3*. In vivo evaluation of Cy7-1-maltotriose at different doses (19nmol (top) and 10nmol (bottom)) in a S. aureus-infected wound murine model.

9. The authors compared the fluorescence of Cy7-1-maltotriose and Cy7-1-maltohexose, the added value would be to show the corresponding optoacoustic data.

- PAI imaging data is added (Figure 4c).

- The PA intensity in the infected thigh using Cy7-1-maltotriose was slightly higher than that using maltohexose, but this was not significant (P= 0.139). When looking at the PA intensity

ratio of infected over control, significantly higher ratio using the maltotriose derivative versus maltohexose was observed (2.5 vs 2 respectively) (Figure 2). Some of those discrepancies are most likely due to the lower sensitivity of PAI to the Cy7 dye compared to FLI as well as lower replicates in this study (n= 3) in the case of Cy7-1-maltohexose.

- See above the added discussion on the limitations of this probe

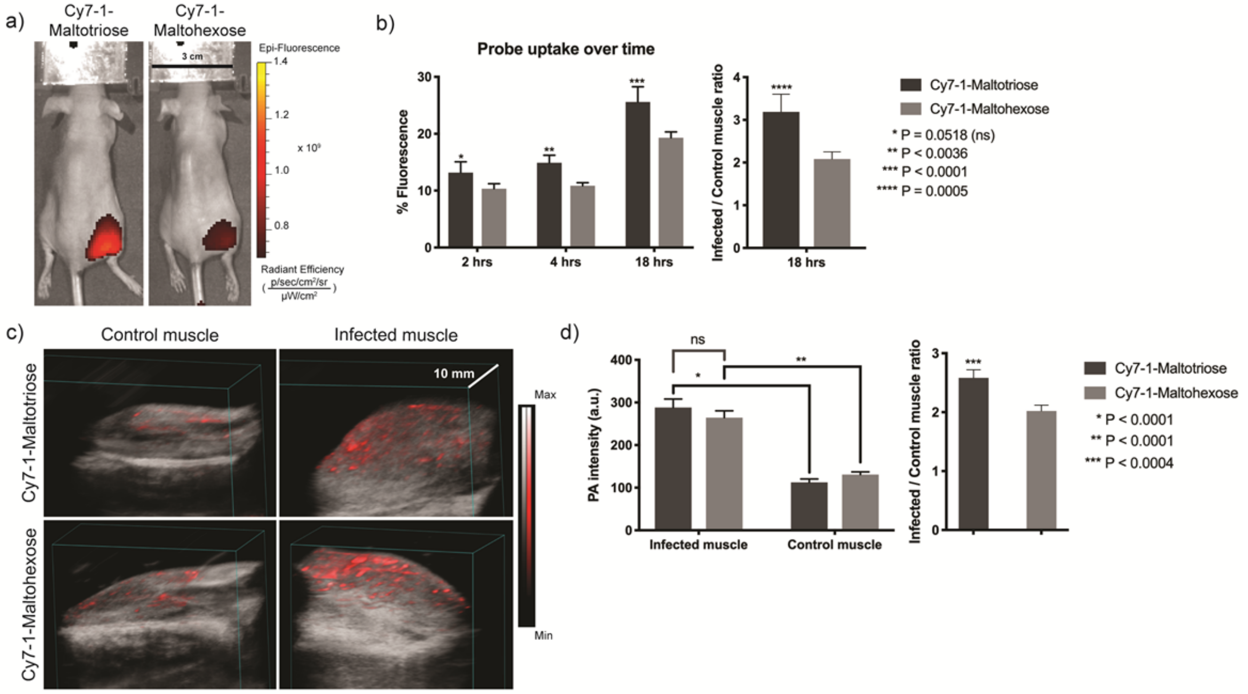

Figure 4. In vivo comparison between Cy7-1-maltotriose and Cy7-1-maltohexose in an E. coli-induced myositis murine model. **a)** *In vivo* fluorescence images at 18 hrs post injection of either Cy7-1-maltotriose (left) or Cy7-1-maltohexose (right) showing accumulation of both probes in the right thigh muscle (*E. coli*). Qualitatively higher fluorescence signal in the infected muscle (right thigh) when injecting Cy7-1-maltotriose (left) compared to Cy7-1-maltohexose (right) is observed. **b)** Bar plot representation of % Fluorescence in the infected muscle compared to whole body (left) and ratio of fluorescence in the infected thigh versus control thigh (right). Starting at 4 and 18 hrs post injection, significantly higher fluorescence signal in the infected muscle (right thigh) when injecting Cy7-1-maltotriose was quantified compared to injecting Cy7-1-maltohexose (n=6, $P < 0.0036$ and n=4, $P < 0.0001$ respectively). **c)** 3D rendered photoacoustic image overlaid on ultrasound image of a mouse 21 hrs post injection of Cy7-1-maltotriose (top) and Cy7-1-maltohexose (bottom). Images show Infected (right thigh) and control (left thigh) muscle. Evident PA signal is observed in the infected thigh muscle when injecting either compounds while minimal signal is observed in the control thigh muscle. **d)** Bar plot representation of the quantified photoacoustic signal intensity in the photoacoustic images acquired 21 hrs after injection of Cy7-1-maltotriose and Cy7-1-maltohexose. The quantified signal shows significantly higher PA signal in the infected muscle compared to control muscle when injecting either compounds (left). No significant difference in PA signal was observed between the two compounds ($P = 0.139$). Significantly higher infected over control PA signal

ratio was observed when injecting Cy7-1-maltotriose (n= 6) compared to Cy7-1-maltohexose (n= 3) (P< 0.0004) (right). Statistical analysis was performed using one-way ANOVA. ns: no statistically significant difference (P > 0.05).

10. Citation 21 and 22 need to be fixed.

Fixed

References

1. Ning, X. *et al.* Maltodextrin-based imaging probes detect bacteria in vivo with high sensitivity and specificity. *Nat Mater* **10,** 602–607 (2011).
2. Takemiya, K. *et al.* Novel PET and Near Infrared Imaging Probes for the Specific Detection of Bacterial Infections Associated With Cardiac Devices. *JACC Cardiovasc Imaging* (2018). doi:10.1016/j.jcmg.2018.02.011
3. Gowrishankar, G. *et al.* Specific Imaging of Bacterial Infection using 6″ -18F-Fluoromaltotriose: A Second Generation PET Tracer Targeting the Maltodextrin Transporter in Bacteria. *Journal of Nuclear Medicine* **58,** 1679–1684 (2017).
4. Axer, A. *et al.* Harnessing the Maltodextrin Transport Mechanism for Targeted Bacterial Imaging: Structural Requirements for Improved in vivo Stability in Tracer Design. *ChemMedChem* **13,** 241–250 (2018).
5. Ning, X. *et al.* PET Imaging of Bacterial Infections with Fluorine□18□Labeled Maltohexaose. *Angew. Chem. Int. Ed. Engl.* **53,** 14096–14101 (2014).
6. Pang, X. *et al.* Bacteria-Responsive Nanoliposomes as Smart Sonotheranostics for Multidrug Resistant Bacterial Infections. *ACS Nano* acsnano.8b09336 (2019). doi:10.1021/acsnano.8b09336
7. Reineck, P. & Gibson, B. C. Near□Infrared Fluorescent Nanomaterials for Bioimaging and Sensing. *Advanced Optical Materials* **5,** 1600446 (2017).
8. van Oosten, M. *et al.* Real-time in vivo imaging of invasive- and biomaterial-associated bacterial infections using fluorescently labelled vancomycin. *Nat Commun* **4,** 2584 (2013).
9. Li, L. L. *et al.* Pathological□Condition□Driven Construction of Supramolecular Nanoassemblies for Bacterial Infection Detection. *Adv. Mater.* **28,** 254–262 (2016).
10. Wang, Y. *et al.* Preclinical Evaluation of Photoacoustic Imaging as a Novel Noninvasive Approach to Detect an Orthopaedic Implant Infection. *J Am Acad Orthop Surg* **25 Suppl 1,** S7–S12 (2017).
11. Tang, E. N., Nair, A., Baker, D. W., Hu, W. & Zhou, J. In vivo imaging of infection using a bacteria-targeting optical nanoprobe. *J Biomed Nanotechnol* **10,** 856–863 (2014).
12. Romero Pastrana, F. *et al.* Noninvasive optical and nuclear imaging of Staphylococcus-specific infection with a human monoclonal antibody-based probe. *Virulence* **9,** 262–272 (2018).
13. Kwon, H.-Y. *et al.* Development of a Universal Fluorescent Probe for Gram-Positive Bacteria. *Angew. Chem. Int. Ed. Engl.* **58,** 8426–8431 (2019).
14. Zhou, J., Qi, G.-B. & Wang, H. A purpurin-peptide derivative for selective killing of Gram-positive bacteria via insertion into cell membrane. *Journal of Materials Chemistry B* **4,** 4855–4861 (2016).
15. Yao, J. *et al.* Multiscale photoacoustic tomography using reversibly switchable bacterial phytochrome as a near-infrared photochromic probe. *Nat. Methods* **13,** 67–73 (2015).
16. Chee, R. K. W., Li, Y., Zhang, W., Campbell, R. E. & Zemp, R. J. In vivo photoacoustic difference-spectra imaging of bacteria using photoswitchable chromoproteins. *J Biomed Opt* **23,** 106006 (2018).
17. Jiang, Y. *et al.* Violacein as a genetically-controlled, enzymatically amplified and photobleaching-resistant chromophore for optoacoustic bacterial imaging. *Sci Rep* **5,** 11048 (2015).
18. Peters, L. *et al.* Phototrophic purple bacteria as optoacoustic in vivo reporters of macrophage activity. *Nat Commun* **10,** 1191 (2019).
19. Kim, T., Zhang, Q., Li, J., Zhang, L. & Jokerst, J. V. A Gold/Silver Hybrid Nanoparticle for Treatment and Photoacoustic Imaging of Bacterial Infection. *ACS Nano* **12,** 5615–5625 (2018).
20. Bowler, P. G., Duerden, B. I. & Armstrong, D. G. Wound microbiology and associated approaches to wound management. *Clin. Microbiol. Rev.* **14,** 244–269 (2001).

Reviewers' comments:

Reviewer #2 (Remarks to the Author):

Although the authors have addressed some of the comments regarding optoacoustic imaging, the following points remain unclear or inaccurate.
1. As suggested previously, it would be more convincing, if the authors could conduct an in vitro experiment to compare the optoacoustic signals from the imaging agents (at a similar concentration for the in vivo cases) with whole blood at the imaging wavelength of 700 nm.
2. Although the authors tried to demonstrate that the optoacoustic signals are primarily from the imaging agents (Figs. 3 and 4), the results are unclear to me. More specifically, in Fig. 3c, the ultrasound images show different background structures before and after injection for the infected muscles. It means that the authors were imaging different sites before and after injection. As a result, the optoacoustic changes may not be due to the infection only. Similar issues also apply to Fig. 4c.
3. The optoacoustic images provided in the manuscript do not show many anatomical or vascular structures, which makes interpretation of the images difficult.
4. In Fig. S7b, it seems that the optoacoustic signal does not linearly increase with the concentration of the agent.

Reviewer #3 (Remarks to the Author):

NA

Point-by-Point Response to Reviewer Comments
Revision 2 Manuscript ID: NCOMMS-18-34556
Title: Fluorescence and Photoacoustic Imaging of Bacterial Infections, Towards a New Generation of Maltotriose-based Probes

Summary of Response:
We would like to thank you for the comments and suggestions. In response, we have conducted the *in vitro* whole blood PA measurements requested by reviewer 2 as well as addressed his/her remaining comments.

Reviewer #2

Reviewer #2 (Remarks to the Author):

Although the authors have addressed some of the comments regarding optoacoustic imaging, the following points remain unclear or inaccurate.
1. As suggested previously, it would be more convincing, if the authors could conduct an in vitro experiment to compare the optoacoustic signals from the imaging agents (at a similar concentration for the in vivo cases) with whole blood at the imaging wavelength of 700 nm.

Per request, we assessed the detection limit of the PA imaging system to Cy7-1-maltotriose in fresh mouse whole blood and observed similar results to that conducted previously in PBS. Interestingly, we again observed no significant difference in PA signal intensity between the 10 and 25 µM solutions. In addition, significantly higher PA signal was observed in the 10µM solution compared to 1µM or only whole blood (0µM solution). It does seem that the detection limit is around 10µM or higher under these conditions and could differ to better or worse with changes in atmosphere (*in vivo* vs *in vitro*), changes in dye, and imaging parameters.

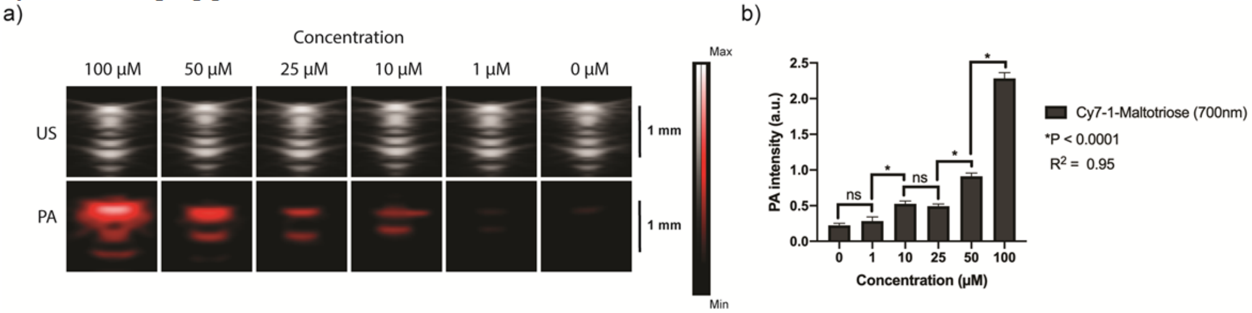

Figure S 8 In vitro PA characterization of Cy7-1-Maltotriose in murine whole blood. a) *Ultrasound and photoacoustic images of a phantom containing different concentrations of Cy7-1-maltotriose upon excitation at 700 nm.* ***b)*** *Quantified PA intensity of different concentrations of Cy7-1-maltotriose in fresh mouse whole blood (n= 6). ns= no statistically significant difference (P > 0.05). Bar graphs show mean and S.E.M. Statistical analysis was performed using one-way ANOVA.*

2. Although the authors tried to demonstrate that the optoacoustic signals are primarily from the imaging agents (Figs. 3 and 4), the results are unclear to me. More specifically, in Fig. 3c, the ultrasound images show different background structures before and after injection for the infected muscles. It means that the authors were imaging different sites before and after injection. As a result, the optoacoustic changes may not be due to the infection only. Similar issues also apply to Fig. 4c.

As we have mentioned in the discussion, one of the main limitations in US/PA imaging is the difficulty to reproduce the same plane of imaging during serial imaging sessions as well its user dependency. In addition, copious amount of research on the instrumentation and imaging parameter side are being investigated to better differentiate intrinsic PA signal from that of the imaging agent (also highlighted in the discussion). In our study, we ought to overcome some of these reproducibility as well as signal differentiation issues by: 1) insuring to compare the image of the same muscle before and after injection of the probe, 2) compare infected versus control muscle, 3) analyze the 3D images which encompasses the region of interest (thigh muscle) and 4) conduct parallel PA and fluorescence imaging studies. While all images were acquired under the same imaging parameters, the decision was to determine the optimal plane to obtain the best images ensuring that the 3D scan encompasses the whole thigh. In addition, all the mice imaged

using PA were also imaged by fluorescence and the observations from the PA imaging study corroborated well with that obtained from the fluorescence imaging study further confirming the effectiveness of the imaging strategy. Namely, the ratio of infected over control thigh muscle of Cy7-1-maltotriose was 2.58 ± 0.14 and 3.19 ± 0.41 while for Cy7-1-maltohexose was 2.02 ± 0.10 and 2.08 ± 0.17 for PA and FLI respectively.

Please refer to figures below which are added to the supporting information (Figure S9 and S10 respectively) which show the US, PA and overlay images as well as highlights key features such as the thigh, rubber pad placed under the thigh and in some cases the patella to better orient the viewer when looking at the images.

In addition, the PA images in Figure 3 and Figure 4 were replaced with the overlay image used in the supporting figures for consistency.

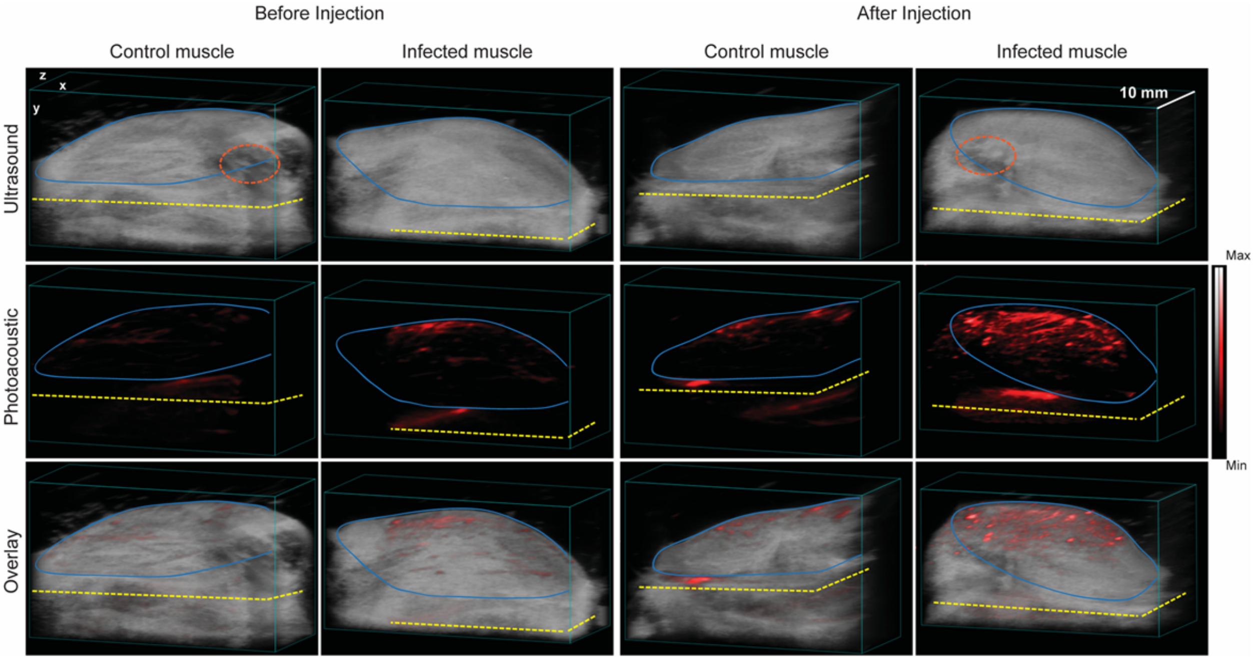

Figure S 9. *3D rendered ultrasound (top), photoacoustic (middle) and overlay (bottom) image of a mouse's left (control) and right (infected) thigh muscle before and 20 hrs post injection of Cy7-1-maltotriose. Orange: delineates the mouse patella (in xy plane), Blue: thigh muscle (Region of interest (ROI) in xy plane), Yellow: rubber padding placed under the thigh.*

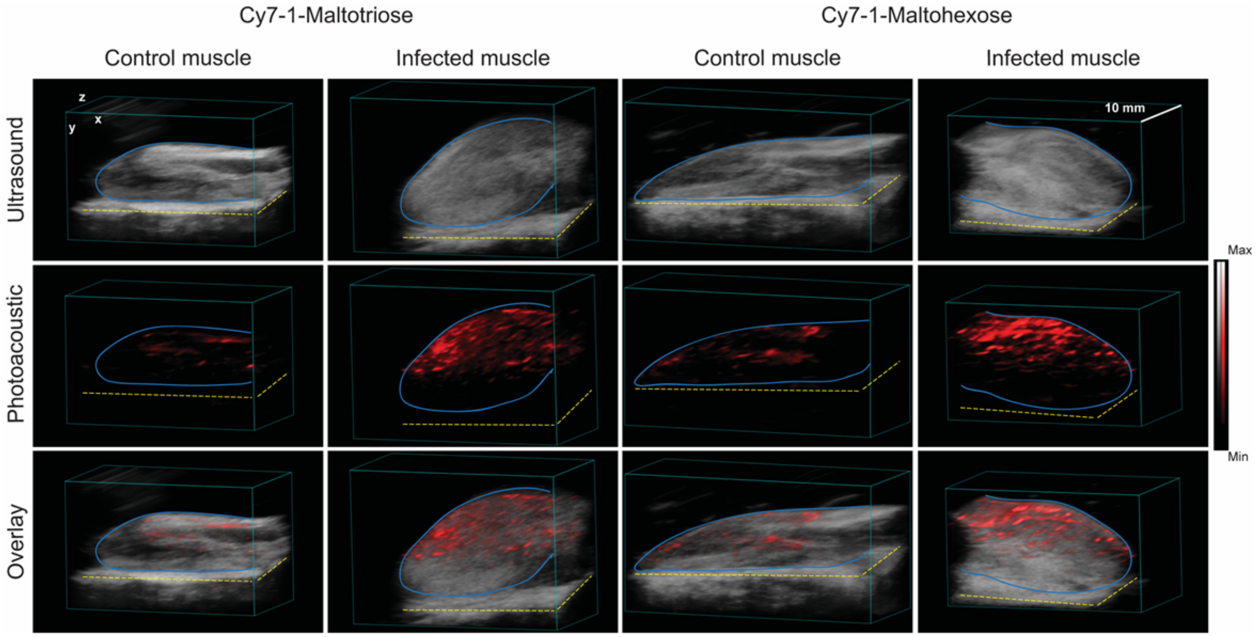

Figure S 10. 3D rendered ultrasound (top), photoacoustic (middle) and overlay (bottom) image of a mouse's left (control) and right (infected) thigh muscle 21 hrs post injection of Cy7-1-maltotriose (left panel) and Cy7-1-maltohexose (right panel). Blue: thigh muscle (Region of interest (ROI) in xy plane), Yellow: rubber padding placed under the thigh.

Section in the discussion:

" While the Cy7 dye used in this imaging agent possess many great characteristics such as *in vivo* stability and optimum pharmacokinetics when bound to maltotriose, this dye is more suitable for fluorescence imaging than photoacoustic imaging. This could explain the higher sensitivity in detecting this probe using fluorescence imaging compared to photoacoustic imaging (Figure S7). In addition, this dye is not approved for clinical use. Hence, we are investigating attachment of a variety of clinically approved dyes as well as dyes more optimal for PAI to further enhance its performance. More specifically, the dye needs to have great plasma stability, high photostability, low toxicity and ease of synthesis in a GMP facility. Furthermore, possessing more red-shifted as well as distinctive absorbance characteristics from intrinsic signals (such as hemoglobin and melanin) will further improve imaging depth and signal quantification respectively. The substitution of the dye may affect the distribution of the imaging agent and a thorough *in vivo* evaluation of the new probes will be necessary. We believe that we have identified the ideal scaffold for an infection imaging agent and with our established synthetic scheme we can quickly synthesize and clinically translate these new probes.

While PAI is growing to be a promising diagnostic tool, it still possesses several limitations which are being addressed. Namely, the limited field of view and imaging depth constricts its utility to localized imaging (no whole-body imaging). Similar to ultrasound imaging, this tool is highly dependent on operators experience and means to automate this technique will help overcome this issue and allow better and more accurate reproducibility of the images (e.g. Robotic handles and software tools to precisely delineate imaging plane for accurate reproducibility) [16]. Alternatively, 3D volume scanning should also assist in reducing the dependence of the operator's experience. Furthermore, several hurdles related to accurate signal quantification (such as differentiating intrinsic signal from imaging agent, reduced accuracy with increased depth) need to be addressed to conduct molecular PAI of disease [16]. Such limitations

can be somewhat mitigated by proper choice of the photoacoustic dye (as mentioned above), choosing the best excitation wavelengths that can assist in differentiating the signal (e.g., background subtraction) and the use of models for fluence correction[16]."

3. The optoacoustic images provided in the manuscript do not show many anatomical or vascular structures, which makes interpretation of the images difficult.

Please refer to the supporting figures above (Figure S9 and S10. As highlighted, the US images do show anatomical information (such as the thigh muscle, Patella in one example, the rubber pad placed under the thigh…). While there are some other sequences and image processing methods which could further distinguish the vascular structure from images, our region of interest here is the thigh muscle and enhancing these features is not the goal of this study.

4. In Fig. S7b, it seems that the optoacoustic signal does not linearly increase with the concentration of the agent.

The data obtained was fitted to a linear regression model and showed an R^2 value of 0.88, this data is from 4 different replicates. In addition, while the whole blood sample showed slightly lower PA intensity (probably due to the opaque nature of blood) similar data to that in PBS was observed and showed even a better fit to the linear regression model R^2= 0.95 (n=6). This improvement is probably due to adding another higher concentration. Below we show the linear regression fitting of the previous data (in revision 1, Figure S7a-b) in PBS as well as the new data in murine whole blood (Figure S8a-b).

a)

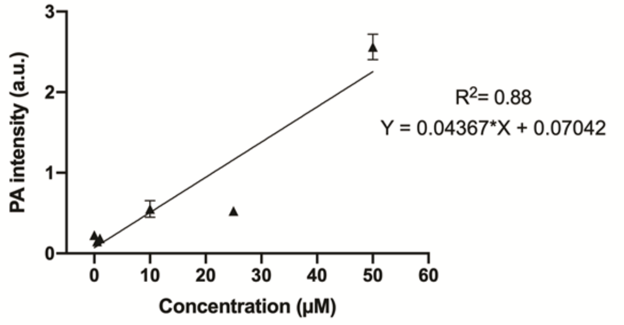

Goodness of Fit	
R square	0.8765
Sy.x	0.3646
Is slope significantly non-zero?	
F	28.39
DFn, DFd	1, 4
P value	0.0060
Deviation from zero?	Significant
Equation	Y = 0.04367*X + 0.07042

b)

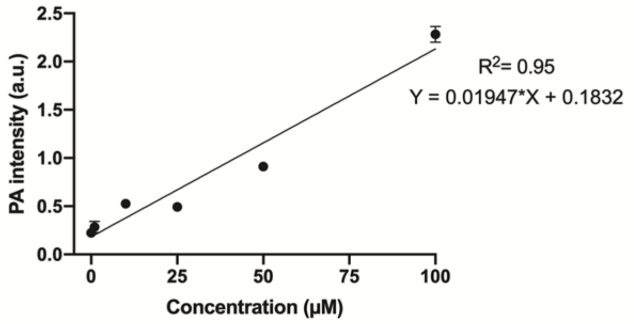

Goodness of Fit	
R square	0.9472
Sy.x	0.1668
Is slope significantly non-zero?	
F	610.1
DFn, DFd	1, 34
P value	<0.0001
Deviation from zero?	Significant
Equation	Y = 0.01947*X + 0.1832

*Figure x. Linear regression fitting of PAI signal intensity of different concentrations of Cy7-1-maltotriose in **a)** PBS and **b)** murine whole blood.*

Reviewers' comments:

Reviewer #2 (Remarks to the Author):

I recommend publication once the following technical issues are resolved.
1. Regarding the linearity measurement (response to comment 4), the PA amplitude from the whole blood sample is 2-3 times lower than that from the PBS sample at similar Cy7-1-maltotriose. The reason given by the authors is the opaque nature of blood. However, the optical penetration depth at 700 nm is greater than the thickness of the sample (~ 1 mm from Fig. S8).
2. The 3D US/PA images (before and after treatment) in Fig. 3c show different structures, which indicates the authors were comparing signals from different tissues. Thus, the experimental results presented in Fig. 3 may not validate the proposed model.

Point-by-Point Response to Reviewer Comments
Revision 3 Manuscript ID: NCOMMS-18-34556
Title: Fluorescence and Photoacoustic Imaging of Bacterial Infections, Towards a New Generation of Maltotriose-based Probes

Summary of Response:

We would like to thank you for your comments. In response, we have addressed reviewer 2 comments and provided some more explanation in the discussion.

Reviewer #2

Reviewer #2 (Remarks to the Author):
I recommend publication once the following technical issues are resolved.
1. Regarding the linearity measurement (response to comment 4), the PA amplitude from the whole blood sample is 2-3 times lower than that from the PBS sample at similar Cy7-1-maltotriose. The reason given by the authors is the opaque nature of blood. However, the optical penetration depth at 700 nm is greater than the thickness of the sample (~ 1 mm from Fig. S8).

We believe that the PA intensity changes observed in whole blood in comparison to a clear PBS solution are not due to interference in optical penetration depth but rather due to effects caused by the change of medium as well as presence of proteins, cells and other organisms which could interfere or absorb some of that energy. In addition, it is known that optical properties of dyes can change slightly in different solvents.
This experiment was conducted to ensure the ability of detecting Cy7-1-maltotriose in blood using PA imaging which is found to be the case for up to 10µM concentration and differences in PA intensity between PBS and whole blood does not interfere with any of our reported results.

2. The 3D US/PA images (before and after treatment) in Fig. 3c show different structures, which indicates the authors were comparing signals from different tissues. Thus, the experimental results presented in Fig. 3 may not validate the proposed model.

The ROI used to measure the PA signal was the thigh muscle and did seem to consistently show higher Fluorescence and PA signal intensity in the infected muscle post injection compared to non-infected muscle or infected muscle before injection. We do acknowledge that overtime there are many structural changes that occur at the imaging site weather due to infection, inflammation or tissue healing which can affect accurate quantitation of the PA signal. As we mentioned in our discussion regards some of the limitations of PA imaging, novel AI, multiplexing and robotic tools are necessary to help 1) better compare PA signals at the site of interest over time during sequential imaging (take into account some of the structural changes happening during disease progression or healing), 2) accurate quantitation of the signal 3) ability to re-image at the exact same plane.

We have further elaborated this in the discussion section of the limitations in PAI.

Addition to discussion:

"While PAI is growing to be a promising diagnostic tool, it still possesses several limitations which are being addressed. Namely, the limited field of view and imaging depth constricts its utility to localized imaging (no whole-body imaging). Similar to ultrasound imaging, this tool is highly dependent on operators experience and means to automate this technique will help overcome this issue and allow better and more accurate reproducibility of the images (e.g., Robotic handles and software tools to precisely delineate imaging plane for accurate reproducibility) [16]. Alternatively, 3D volume scanning should also assist in reducing the dependence of the operator's experience. Furthermore, several hurdles related to accurate signal quantification (such as differentiating intrinsic from imaging agent signal, reduced accuracy with increased depth and effects of disease progression or tissue healing on PA signal intensity) need to be addressed to conduct molecular PAI of disease [16]. Such limitations can be somewhat mitigated by proper choice of the photoacoustic dye (as mentioned above), choosing the best excitation wavelengths that can assist in differentiating the signal (e.g., background subtraction), the use of models for fluence correction[16] and other AI tools which can take into account any PA signal intensity changes occurring during structural changes cause by disease progression, inflammation and/or healing."

REVIEWERS' COMMENTS:

Reviewer #2 (Remarks to the Author):

At this point, I recommend acceptance with the following optional revisions.
1. Linearity will be important to the readers. Despite the reasons listed by the authors, I believe that a well-controlled in vitro measurement to clearly characterize the probes would be more convincing.
2. A couple of comments in the manuscript (starting from Line 420) are inaccurate for the following reasons. 1) Although the limited-view problem exists in linear-array based PA imaging, full-view PA imaging has been demonstrated (see the literature below). 2) Whole-body small-animal PA imaging has been shown by multiple groups as well.
a. Merčep, E., Herraiz, J.L., Deán-Ben, X.L. et al. Transmission–reflection optoacoustic ultrasound (TROPUS) computed tomography of small animals. Light Sci Appl 8, 18 (2019) doi:10.1038/s41377-019-0130-5
b. Li, L., Zhu, L., Ma, C. et al. Single-impulse panoramic photoacoustic computed tomography of small-animal whole-body dynamics at high spatiotemporal resolution. Nat Biomed Eng 1, 0071 (2017) doi:10.1038/s41551-017-0071

Point-by-Point Response to Reviewer Comments
Revision 4 Manuscript ID: NCOMMS-18-34556
Title: Maltotriose-based Probes for Fluorescence and Photoacoustic Imaging of Bacterial Infections

Summary of Response:

We would like to thank you for your comments. In response, we have addressed reviewer 2 comments.

Reviewer #2

Reviewer #2 (Remarks to the Author):
At this point, I recommend acceptance with the following optional revisions.
1. Linearity will be important to the readers. Despite the reasons listed by the authors, I believe that a well-controlled in vitro measurement to clearly characterize the probes would be more convincing.
We added the linearity assessment to the supporting information (Supplementary Figure 10)

2. A couple of comments in the manuscript (starting from Line 420) are inaccurate for the following reasons. 1) Although the limited-view problem exists in linear-array based PA imaging, full-view PA imaging has been demonstrated (see the literature below). 2) Whole-body small-animal PA imaging has been shown by multiple groups as well.
a. Merčep, E., Herraiz, J.L., Deán-Ben, X.L. et al. Transmission–reflection optoacoustic ultrasound (TROPUS) computed tomography of small animals. Light Sci Appl 8, 18 (2019) doi:10.1038/s41377-019-0130-5
b. Li, L., Zhu, L., Ma, C. et al. Single-impulse panoramic photoacoustic computed tomography of small-animal whole-body dynamics at high spatiotemporal resolution. Nat Biomed Eng 1, 0071 (2017) doi:10.1038/s41551-017-0071

The focus in the discussion is to address the current and possible limitations in **clinical PA imaging (**as opposed to small animals where full view full body PA can be achieved) and how to overcome some of those issues in the future. To overcome this confusion, we have added several statements in the discussion to distinguish that the limitations discussed are in human imaging as well as included a statement about the developed small animal whole-body PA imaging systems (provided by the reviewer) which could improve the **preclinical evaluation** of the novel PA probes.
